# Osimertinib plus consolidative radiotherapy for advanced *EGFR* mutant non–small cell lung cancer: a multicentre, single-arm, phase 2 trial

**DOI:** 10.1016/j.eclinm.2025.103435

**Published:** 2025-08-26

**Authors:** Sagus Sampath, Sawsan Rashdan, Puneeth Iyengar, Townes A. Mickel, Song Zhang, Chul Ahn, Ang Gao, Jonathan E. Dowell, Yuanyuan Zhang, Kenneth D. Westover, Suzanne M. Cole, Arya Amini, Adam Rock, Erminia Massarelli, Marianna Koczywas, David E. Gerber

**Affiliations:** a Department of Radiation Oncology, City of Hope National Medical Center, Duarte, CA, USA; b Division of Hematology-Oncology, University of Texas Southwestern Medical Center, Dallas, TX, USA; c Harold C. Simmons Comprehensive Cancer Center, University of Texas Southwestern Medical Center, Dallas, TX, USA; d Department of Radiation Oncology, University of Texas Southwestern Medical Center, Dallas, TX, USA; e Peter O'Donnell Jr. School of Public Health, University of Texas Southwestern Medical Center, Dallas, TX, USA; f Department of Medical Oncology, City of Hope National Medical Center, Duarte, CA, USA

**Keywords:** Consolidation, *EGFR* mutant non-small cell lung cancer, Pneumonitis, Stereotactic radiation therapy

## Abstract

**Background:**

Despite high response rates to epidermal growth factor receptor (EGFR) inhibitors, patients with advanced *EGFR* mutant non-small cell lung cancer (NSCLC) generally experience disease progression within 2 years. We evaluated whether consolidative radiation therapy (RT) to residual sites of disease at the time of expected best response to EGFR inhibition prolongs disease control.

**Methods:**

This multicentre, single-arm phase 2 trial was conducted at two sites in the USA. Eligible patients (aged ≥18 years) had advanced *EGFR* mutant (exon 19 or 21) NSCLC not restricted by number, site, or size of metastases; ECOG 0–2; and no prior treatment with EGFR or immune checkpoint inhibitors. Patients with stable or responding disease after 8 weeks of osimertinib 80 mg orally daily received radiation therapy (RT) to persisting lesions, followed by continued osimertinib until progression or intolerance. The primary endpoint was progression-free survival (PFS) in all participants who received at least one dose of osimertinib, assessed radiographically every 8 weeks. Secondary endpoints were toxicity, duration on osimertinib, and overall survival (OS). This trial is registered with Clinicaltrials.gov, NCT03667820.

**Findings:**

Between Oct 15, 2018, and July 1, 2021, 42 patients (32 female, 10 male) were enrolled and initiated osimertinib, of whom 32 (76%) received consolidative RT, primarily stereotactic RT. The most common reasons RT was not administered were insufficient residual disease (10%) and inadequate response (5%). At a median follow-up of 35.7 months, median PFS was 32.3 months (95% CI, 21.9–51.7), median OS was 45 months (95% CI, 39.3–56.4), and median duration of osimertinib was 32.4 months. Osimertinib-related toxicities, including skin, nail, and gastrointestinal events, occurred at expected rates and were almost always grade 1–2. Two patients (5%) developed pneumonitis, including one grade 4 event.

**Interpretation:**

These findings show osimertinib plus consolidative RT was well tolerated and demonstrates promising efficacy in patients with advanced *EGFR* mutant NSCLC. Because this approach may be less complex and less toxic than multi-agent targeted therapy regimens for this population, the results of ongoing randomised trials testing similar strategies are awaited.

**Funding:**

10.13039/100004325AstraZeneca and the Biostatistics Shared Resource, UT Southwestern Harold C. Simmons Comprehensive Cancer Center.

**Research in context**

**Evidence before this study**

We searched PubMed for articles published, in any language, between database inception and April 10, 2025, using the terms (“epidermal growth factor receptor” OR “EGFR”) AND (“lung cancer” OR “lung adenocarcinoma” OR “non-small cell lung cancer”) AND (“advanced” OR “metastatic”) AND (“radiation”), yielding a total of 63 articles. Despite high initial response rates to epidermal growth factor receptor (EGFR) inhibitors, patients with advanced *EGFR* mutant non-small cell lung cancer (NSCLC) generally experience disease progression within 2 years. To improve outcomes and prolong disease control, clinical trials have incorporated local therapy such as radiation therapy (RT) up front or at the time of oligoprogression. However, few studies have examined the role of consolidative RT at the time of expected best response to EGFR inhibition.

**Added value of this study**

To the best of our knowledge, this is the first study to evaluate the efficacy and tolerability of consolidation radiation therapy (RT) administered at time of expected best response (8–10 weeks) to the third-generation EGFR inhibitor osimertinib in patients with advanced *EGFR* mutant non-small cell lung cancer (NSCLC) regardless of the number, size, or sites of initial metastases. The primary endpoint of progression-free survival (PFS) was met, with median PFS of 32 months compared to a historical control of 19 months for osimertinib alone. Osimertinib-related toxicities occurred at expect rates and severity, and only 5% of patients developed pneumonitis.

**Implications of all the available evidence**

The available evidence and the present study demonstrate that incorporating RT into late-generation EGFR inhibitor treatment regimens appears safe and potentially efficacious in a broad and generalisable patient population with advanced *EGFR* mutant NSCLC. Because emerging multi-agent regimens for this clinical scenario may add considerable complexity and toxicity beyond those of single-agent EGFR inhibition, our findings provide evidence supporting the comparatively straightforward regimen of EGFR inhibition plus consolidation RT. Results from randomised trials of this strategy (e.g., NORTHSTAR, NCT03410043) are awaited.

## Introduction

Despite frequent, rapid, and profound responses to epidermal growth factor receptor (EGFR) inhibitors, patients with advanced *EGFR* mutant NSCLC inevitably develop resistance within months to years. Multiple strategies to prolong the clinical benefit of EGFR inhibitors have been explored. Regimens combining EGFR inhibitors with antiangiogenic agents or platinum-based chemotherapy have demonstrated improved outcomes compared to single-agent EGFR inhibition.^1^, ^2^, ^3^, ^4^ Treatment with amivantamab (an EGFR-MET bispecific antibody) and lazertinib (a third-generation EGFR inhibitor) also results in improved PFS and OS compared to osimertinib monotherapy.^5^^,^^6^ Benefits of these multi-agent treatments come at a price, however, as they may increase complexity, cost, and toxicity.

Radiation therapy (RT) has become a widely employed strategy to prolong disease control with molecularly targeted therapies, with clinicians delivering stereotactic or other RT techniques to progressing sites while continuing the original systemic treatment. Promising efficacy coupled with convenience and favourable safety profiles led to trials incorporating broader NSCLC populations and to earlier use of RT, as a consolidative rather than rescue therapy. Phase 2 trials have demonstrated an improvement in progression-free survival (PFS) and overall survival (OS) for patients with oligometastatic NSCLC receiving consolidation RT following an induction period of first-line systemic therapy, although representation of *EGFR* mutant cases was limited.^7^^,^^8^ A randomised trial in *EGFR* mutant NSCLC also demonstrated improved outcomes,^9^ but this study employed first-generation EGFR inhibitors that feature substantially different efficacy and toxicity than the third-generation drugs now considered standard of care.

Here, we evaluated the third-generation EGFR inhibitor osimertinib plus consolidative RT administered at the time of anticipated best response in advanced *EGFR* mutant NSCLC. Because osimertinib exhibits frequent, profound responses, and “oligometastatic” may be a challenging concept to implement in routine clinical practice, we did not restrict enrolment according to number or sites of metastases.

## Methods

### Study design and participants

This multicentre, single-arm, phase 2 trial (NCT03667820) was approved by the institutional review boards of all participating centres (UTSW IRB # STU 122017-017): UT Southwestern Medical Center (Dallas, Texas, USA) and City of Hope Cancer Center (Duarte, California, USA). Enrolled patients provided written informed consent prior to undergoing any study-related procedures. No randomisation or masking/blinding were involved.

Eligible patients had previously untreated advanced (AJCC 8th edition stage IV) NSCLC harbouring an *EGFR* sensitising mutation (exon 19 deletion or exon 21 L858R), regardless of the number of metastases. All patients underwent dedicated brain magnetic resonance imaging (MRI) unless contraindicated. Additional eligibility criteria included ECOG performance status 0–2, adequate bone marrow and organ function. Prior palliative RT was allowed; however, a minimum of one non-radiated baseline lesion was required at time of enrolment. Patients with asymptomatic or previously treated brain metastases were eligible. A complete list of eligibility criteria can be found in the full protocol (available in Supplement 1). Patient sex was recorded from the electronic health record.

### Study treatment

Patients received osimertinib at the standard dose and schedule of 80 mg orally daily. Supportive care for dermatologic and gastrointestinal toxicities was provided as needed. Safety labs were monitored throughout treatment. Osimertinib dosing could be interrupted, reduced, or discontinued for toxicities, as stipulated in the study protocol (Supplement 1). Patients underwent re-staging computed tomography (CT) and/or positron emission tomography (PET)/CT imaging (per treating physician discretion) ± brain MRI (for patients with known brain metastases) following 8–10 weeks of osimertinib, a time-point selected to achieve RT delivery 10–12 weeks after initiation of systemic therapy, similar to other NSCLC trials of consolidative RT.^7^^,^^8^ Patients with stable disease (SD), partial (PR) or complete response (CR) (based on RECIST 1.1 criteria) remained on study and were referred for consolidation RT.

At that point, the treating radiation oncologist reviewed available imaging studies to determine appropriate RT targets, reviewing the plan with the Radiation Oncology study co-chair prior to treatment. Up to six sites or organs could receive RT, with intent to treat all sites of residual disease. Irradiated sites needed to be localisable on CT simulation and meet normal tissue dose constraints.

Although up to 10 brain metastases with maximal size ≤4 cm could be treated with RT, treating radiation oncologists were counselled to approach this anatomic site distinctly. With osimertinib alone, the median time to best intracranial response exceeds 3 months; the intracranial complete response rate of osimertinib exceeds 75%; and the intracranial lesion-level local failure rate is only 5% at 2 years.^10^^,^^11^ Conversely, up to 40% of patients may develop eventual central nervous system (CNS) progression.^12^ Accordingly, consolidative RT was generally not recommended for responding brain metastases.

To limit the risk of pulmonary complications, osimertinib was withheld from 3 days prior to thoracic RT until 3 days after RT completion. For non-thoracic targets, osimertinib could be continued or withheld during RT based on treating clinician discretion.

Following RT, patients underwent imaging studies (CT ± PET-CT) every 2 months for 1 year, then every 3 months thereafter. For patients with baseline brain metastases, brain imaging was performed at similar intervals. For those without brain metastases, it was performed yearly. Patients with CR who did not receive RT remained on study and were followed similarly. Toxicity was measured using NCI Common Terminology Criteria for Adverse Events (CTCAE) version 4.0 (later updated to 5.0). Osimertinib was continued until intolerance or disease progression not amenable to additional RT.

### RT technique

Patients could receive stereotactic RT over 1, 3, or 5 fractions, or hypofractionated RT over 15 fractions, regimens modelled off the NRG LU-002 trial.^13^ Supplemental Table S1 lists the prescription doses by organ site. Common doses were 24 Gy (1 fraction), 27–30 Gy (3 fractions), and 35 Gy (5 fractions). The permitted RT plans generally featured lower doses than those typically administered when stereotactic RT is used alone as definitive treatment for early-stage NSCLC (e.g., 54 Gy in three 18-Gy fractions in RTOG 0618^14^). The rationale for this approach reflected the concomitant administration of systemic therapy, which may itself convey anti-tumour efficacy and/or enhance toxicity. Such “sub-ablative” regimens in the advanced NSCLC setting have shown excellent tolerance, high rates of local control, and clear clinical benefit when combined with systemic therapy (PFS HR 0.30; 95% CI, 0.11–0.82; *P* = 0.01) in a prior randomised phase 2 trial.^8^

Based on the 8–10 week imaging study, up to six sites/organs could receive RT. Bone metastases needed to have persistent lytic components. Given the small number of participating sites, we did not perform centralised radiation plan review. Additional RT was allowed at the time of progression if deemed appropriate and feasible by the treating clinicians.

Radiation equipment, technique, and quality assurance were standardised across centres. RT was delivered with Varian (Varian Inc, Palo Alto, CA) linear accelerators with on-board cone beam CT imaging and pre-treatment image guidance for localisation prior to each fraction. Radiation delivery consisted of volumetric modulated arc therapy (VMAT) technique with micro-multi-leaf collimation and respiratory management when applicable.

### Statistical analysis

The primary endpoint of the trial was PFS, defined as the time from osimertinib initiation until the time of objective progression (as defined by the investigator according to RECIST 1.1) or death. Secondary endpoints included duration of response, objective response rate (ORR), OS, time to subsequent RT or death, time to osimertinib discontinuation or death, safety, and tolerability. Data was locked for analysis on January 31, 2024.

We calculated that a sample size of 42 patients would achieve 80% power to detect the difference between the null hypothesis (median PFS of 19 months, based on the FLAURA trial of first-line osimertinib monotherapy for advanced *EGFR* mutant NSCLC^15^) and the alternative hypothesis (median PFS of 30 months) at a one-sided significance level of 0.1. We assumed that all enrolled patients would receive consolidative RT, an accrual period of 36 months, and a follow-up period of 12 months. The sample size was estimated using the Southwest Oncology Group (SWOG) sample size calculator (https://www.swogstat.org/stat/public/one_survival.htm). We used the Kaplan–Meier method to estimate PFS, duration on osimertinib, and OS. We summarised descriptive summary statistics using medians, quartiles, and percentages. Response and adverse event rates were estimated using the exact binomial method. All outcomes and analyses were prespecified. No sensitivity or post-hoc analyses were performed.

### Role of the funding source

The study funder was not involved in study design, data collection, data analysis, data interpretation, or report writing.

## Results

**Fig. 1:**
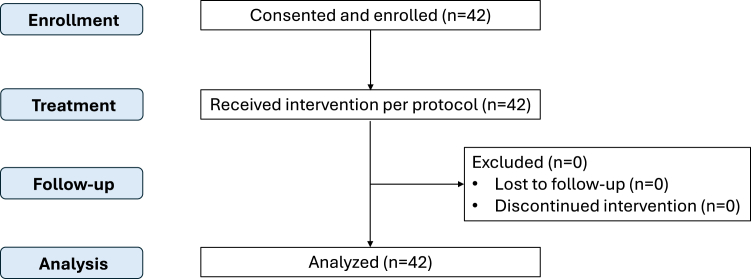
CONSORT flow diagram.

Between Oct 15, 2018, and July 1, 2021, 42 patients (UT Southwestern, n = 25; City of Hope, n = 17) were enrolled. The trial CONSORT flow diagram is shown in Fig. 1. 27 patients (64%) had two or more sites of metastatic disease, most commonly bone (62%) and brain (50%). Additional patient characteristics are shown in Table 1.

**Table 1:** Baseline characteristics of enrolled patients.

Characteristic	Mean (±SD) or n (%) N = 42
Age (years)	65 ± 12.6
Sex	
Male	10 (24)
Female	32 (76)
Race-ethnicity	
Caucasian non-hispanic	23 (55)
Asian/Asian-American	17 (40)
Caucasian hispanic	2 (4)
Smoking status	
Never	28 (67)
Former	14 (33)
ECOG performance status	
0	20 (48)
1	22 (52)
*EGFR* Mutation	
Exon 19	21 (50)
Exon 21	21 (50)
Number of metastatic organs^a^	
1	15 (36)
2	12 (29)
3	7 (17)
4	1 (2)
≥5	7 (17)
Number of metastatic lesions at diagnosis^a^	
1	6 (14)
2–3	6 (14)
4–5	3 (7)
6–10	6 (14)
>10	21 (50)
Sites of metastatic disease^a^^,^^b^	
Bone	26 (62)
Brain	21 (50)
Lung (excluding primary)	17 (40)
Pleural effusion/nodules	11 (24)
Liver	6 (14)
Adrenal	1 (2)
Palliative RT delivered prior to trial enrolment	
None	33 (79)
Bone	6 (14)
Brain	4 (10)

RT, radiation therapy; SD, standard deviation.

a Reported on a per-patient basis.

b Percentages add up to >100 because individual patients may have had multiple sites of metastases.

All enrolled patients started osimertinib and continued osimertinib until the initial imaging time-point (8–10 weeks); 32 patients (76%) received consolidative RT (Table 2). For the 10 patients who did not receive RT, the most common reason was insufficient residual disease, followed by inadequate response. One patient did not proceed with RT to the lung due to onset of cough and lung radiographic changes overlapping with a pre-trial course of palliative RT to the spine. Sites irradiated most frequently (in order of decreasing frequency) were lung, bone, and thoracic regional lymph nodes. No patients received consolidative RT to the brain. RT was delivered to 1–2 sites in 28 cases (67%). Details of RT plans are listed in Supplemental Table S1. For lung lesions, 34 or 35 Gy in 5 fractions was the most used regimen, followed by 30 Gy in 3 fractions. Bone (including spine) was most commonly treated with 30 Gy/3 fractions. Overall, 28 patients (88%) received only stereotactic (five or fewer fractions) RT regimens at consolidation, with the remainder being treated with hypofractionated (>5 fractions) regimens. Although osimertinib interruption was mandated only during thoracic RT, treating clinicians chose to withhold osimertinib during all RT regimens.

**Table 2:** Consolidative RT details for all enrolled patients.

Characteristic	N (%)
RT administered	
Yes	32 (76)
No	10 (24)
Reason RT not administered	
Insufficient residual disease	5 (12)
Inadequate response	2 (5)
Functional decline	2 (5)
Toxicity	1 (5)
N/A (RT administered)	32 (76)
Number of lesions irradiated	
1	18 (43)
2	10 (24)
3	2 (5)
4	2 (5)
N/A (RT not administered)	10 (24)
Sites irradiated^a^	
Lung primary	25 (60)
Bone	13 (31)
Lung primary and regional lymph nodes	6 (14)
Lung (metastasis)	3 (7)
Adrenal	1 (2)
Liver	1 (2)
None (RT not administered)	10 (24)
Osimertinib held during RT	
Yes	32
No	0
N/A (RT not administered)	10

N/A, not applicable; RT, radiation therapy.

a Percentages add up to >100 because individual patients may have had multiple sites irradiated.

**Fig. 2:**
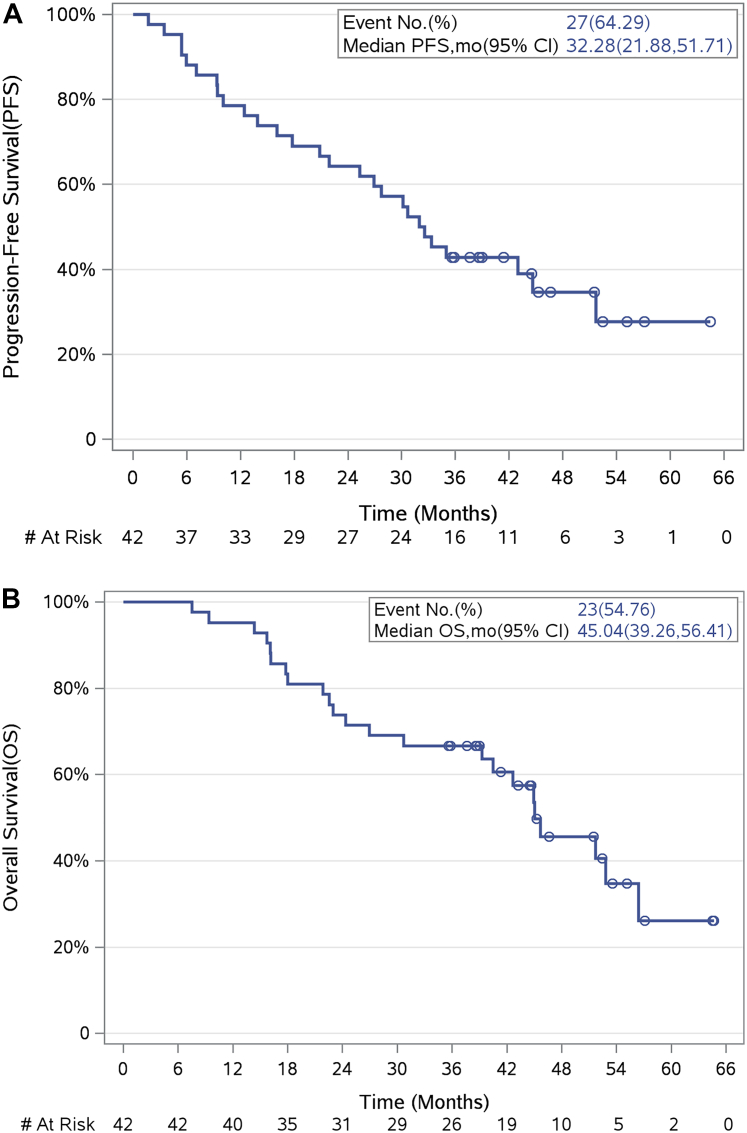
Progression-free survival (PFS) **(A)**, and overall survival (OS) **(B)**.

**Fig. 3:**
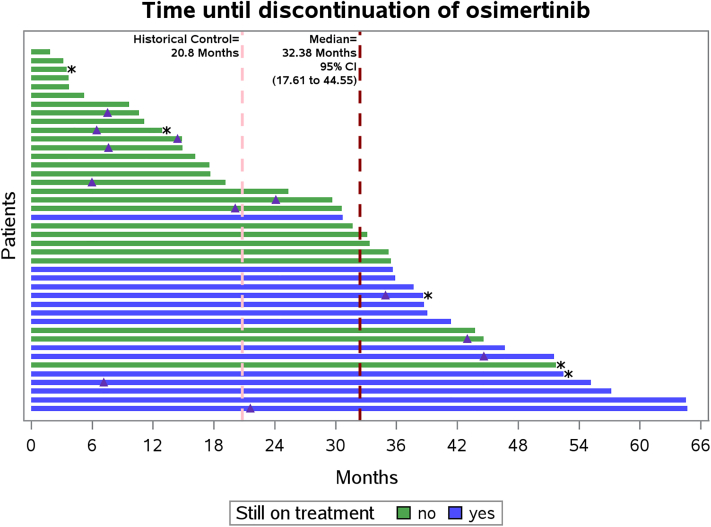
Duration of osimertinib therapy. *Green*, patients who have discontinued study treatment. *Blue*, patients who remain on study treatment. Asterisk denotes those patients with insufficient residual disease at 8 weeks who did not receive consolidative radiation therapy (RT). *Red and pink* dashed lines, respectively, indicate median duration of osimertinib in the present study, and in the FLAURA trial of first-line osimertinib monotherapy. *Purple* triangles indicate RT at time of oligoprogression.

After a median follow-up of 35.7 months, median PFS was 32.3 months (95% CI 21.9–51.7 months) (Fig. 2A). At progression, 12 patients (23%) received RT to a total of 15 sites (primarily bone and lung; see Supplemental Table S1), of whom 10 patients (83%) received only stereotactic RT regimens. Median PFS following salvage RT was 5.0 months (range 3–51 months). Out of seven patients who had local progression in a previously irradiated site, only two were treated with stereotactic doses. Median OS was 45 months (95% CI 39.3–56.4 months) (Fig. 2B). Median duration of osimertinib was 32.4 months (95% CI 17.6–44.6 months), accounting for censoring using the Kaplan–Meier method (Fig. 3), with oligo-progressive patients who received RT showing benefit. At the time of analysis, 15 patients (36%) remained on study receiving osimertinib, including four who had received RT at oligoprogression and accounting for all censored cases.

In general, the study regimen of osimertinib plus consolidative RT was well tolerated (Table 3). Characteristic osimertinib-related toxicities (almost all grade 1–2), including skin, nail, and gastrointestinal events, occurred at expected rates. Two patients developed pneumonitis, including one high-grade event. This case featured pleural disease at baseline and good response to osimertinib after 8–10 weeks. At consolidation, stereotactic RT was administered to a 1 cm left upper lobe lesion, with osimertinib held per protocol. The patient developed grade 4 pneumonitis 32 days after RT was completed. Osimertinib was discontinued. Given the small size of the treated lung lesion and the low rate of pneumonitis seen with osimertinib, the event was considered possibly related to both RT and osimertinib. On dosimetric analysis, the gross tumour volume measured 3 cc; the total lung volume receiving 20 Gy or higher (V20) was 1%, and the V5 was 6.2%. No other radiation-associated grade ≥3 events were observed. No late adverse events attributable to radiation, such as pulmonary fibrosis, oesophageal stenosis, cerebral radionecrosis, or bone fracture, were observed.

**Table 3:** Treatment-emergent adverse events occurring in ≥10% of patients.

Adverse event	n (%)	
	Any grade	Grade ≥3
Fatigue	27 (63)	1 (2)
Diarrhea	25 (58)	1 (2)
Cough/dry cough/productive cough	23 (53)	
Dry skin	23 (53)	
Arm/shoulder pain	15 (35)	3 (7)
Headache	15 (35)	
Pruritis	15 (35)	
Neutrophil decrease	14 (33)	
WBC decrease	14 (33)	
Acneiform rash	13 (30)	
Nausea	13 (30)	
Lymphocyte decrease	12 (28)	3 (7)
Abdominal pain	11 (26)	
Anorexia	11 (26)	
Constipation	11 (26)	
Paronychia/Nail Infection	11 (26)	1 (2)
Dry mouth	10 (23)	
Dysgeusia	10 (23)	
Vomiting	10 (23)	
ALT/AST increase	9 (21)	3 (7)
Dyspnea	9 (21)	
Fever	9 (21)	
Platelets decrease	9 (21)	
Epistaxis	8 (19)	
Hypoglycemia	8 (19)	
Alopecia	7 (16)	
Chills	7 (16)	
Hyponatremia	7 (16)	1 (2)
Creatinine increase	6 (14)	
Hypermagnesemia	6 (14)	
Hypertension	6 (14)	3 (7)
Nasal Congestion	6 (14)	
Palpitations	6 (14)	
Urinary Tract Infection	6 (14)	
Anemia	5 (12)	
Dysphagia	5 (12)	
Insomnia	5 (12)	
Hypocalcemia	4 (9)	1 (2)
Hypokalemia	4 (9)	1 (2)
Catheter-related infection	2 (5)	1 (2)
Neck pain	2 (5)	1 (2)
Pneumonitis	2 (5)	1 (2)
Intracranial hemorrhage	1 (2)	1 (2)
Muscle Weakness	1 (2)	1 (2)
Syncope	1 (2)	1 (2)

## Discussion

This single-arm phase 2 trial evaluated the impact of adding early RT as a consolidation strategy to residual sites of disease in advanced *EGFR* mutant NSCLC treated with osimertinib. Recognising the potential for early and profound responses to osimertinib, as well as the challenge of defining an oligometastatic phenotype, the trial placed no restrictions on the number or location of disease sites at diagnosis. As a result, about one-fifth of patients had ≥4 sites of metastatic disease at baseline, almost two-thirds had bone metastases, and half had brain metastases. Although the trial permitted both stereotactic and hypofractionated RT regimens, only about 10% of patients receiving RT received hypofractionated treatment, in all cases to the thorax.

Recognising the clear limitations of cross-trial comparisons, the regimen of osimertinib with early consolidative RT appeared to demonstrate favourable efficacy. In the present trial, the median PFS exceeded 30 months and median OS exceeded 45 months. In the FLAURA trial of first-line osimertinib monotherapy, median PFS was 19 months (95% CI 15.2–21.4 months) and median OS was 39 months (95% CI 34.5–41.8 months).^15^ Our results also compare favourably with intensified first-line regimens, such as osimertinib plus consolidation platinum-pemetrexed chemotherapy (median PFS 26 months),^4^ osimertinib plus bevacizumab (median PFS 22 months),^16^ and amivantamab plus lazertinib (median PFS 24 months).^6^

The present trial joins a growing group of trials examining EGFR inhibition combined with RT for patients with advanced EGFR mutant NSCLC (Table 4). The randomised SINDAS trial took a different approach, delivering RT up front rather than after the initiation of systemic therapy. The trial excluded patients with brain metastases and limited eligibility to 5 or fewer discrete sites of disease.^9^ With a median PFS of 20 months (one year shorter than that in the present trial), potential reasons for this difference include (1) the type of systemic therapy (1st vs 3rd generation EGFR inhibitor) and (2) the timing of RT. Specifically, up-front RT may need to address a greater burden of disease compared to consolidative RT; it also results in delaying the initiation of systemic therapy, which theoretically might have greater implications than treatment interruption. In a smaller randomised phase II trial featuring aspects of both SINDAS (first-generation EGFR inhibitor) and the current study (consolidative RT after 2 months of systemic therapy), the addition of RT to EGFR inhibitor therapy resulted in a doubling of PFS, from 9 to 18 months.^17^ A phase 3 trial showed improved PFS and OS when conventionally fractionated (60 Gy RT to the lung primary and regional nodes) was added to the first-generation EGFR inhibitor icotinib.^19^ However, 12% of patients in the combination arm developed grade ≥3 toxicity (mainly oesophagitis and pneumonitis), which could reflect the relatively large radiation volumes.

**Table 4:** Prospective phase 2/3 trials of first-line EGFR inhibitor therapy and RT in advanced NSCLC.

Study	N	RT timing (weeks after starting EGFR inhibitor)	RT dosing	EGFR inhibitor (n, %)	Baseline disease burden n (%)		Brain meta-stases n (%)	Median follow-up (mos)	PFS (mos)	OS (mos)
					≤5 sites	>5 sites				
SINDAS trial, Wang et al.,^9^ randomised phase 2	68 (arm B)	Pre-EGFR inhibitor	25–40 Gy/5 fractions	1st generation gefinitib (32, 47) erlotinib (30, 44) icotinib (6, 9)	68 (100)	0 (0)	0 (0)	23.6	20.2	25.5
Peng et al.,^17^ randomised phase 2	30 (arm B)	12 weeks	30–50 Gy/5 fractions	1st generation gefinitib (NR) erlotinib (NR) icotinib (NR)	31 (100)	0 (0)	6 (20)	29.4	17.6	33.6
LUNG-SORT trial, Zhou et al.,^18^ single arm phase 2	61	Median 3.7 months (IQR, 1.0–5.9 months)	27–45 Gy/3 fractions, 50 Gy/5 fractions	3rd generation osimertinib (49, 80) almonertinib (12, 20)	37 (61)	24 (39)	22 (36)	21.1	29.9	Not reached
Sun et al.,^19^ randomised phase 3	59 (arm B)	<2 weeks	60 Gy/30 fractions	1st generation icotinib (100)	25 (42)	24 (58)	24 (41)	27.5	17.1	34.4
Current study, single arm phase 2	42	10–12 weeks	1,3,5, and 15-fraction regimens (per NRG LU-002)^10^	3rd generation osimertinib (42, 100)	15 (34)	27 (68)	21 (50)	35.7	32.3	45

EGFR, epidermal growth factor receptor; IQR, interquartile range; NR, not reported; OS, overall survival; PFS, progression-free survival; RT, radiation therapy.

A recent single-arm phase 2 trial (LUNG-SORT) incorporated consolidative RT to residual disease sites after initiation of third-line EGFR inhibitor therapy (80% osimertinib).^18^ A key difference between LUNG-SORT and the present study is the timing and nature of patient enrolment. In LUNG-SORT, patients were enrolled after initiation of systemic therapy and were required to have oligo-residual disease (≤5 extracranial lesions; ≤3 intracranial lesions). Reflecting these design considerations, patients enrolled in the current study (which did not restrict eligibility according to number, size, or site of metastases) tended to have a greater burden of disease (based on number of metastatic sites) than those in LUNG-SORT. Furthermore, the current trial provides an estimate of the proportion of advanced *EGFR* mutant NSCLC cases starting EGFR inhibitor for whom consolidative RT appears feasible (approximately three-fourths) and the main reason it is not administered (insufficient residual disease). Although the median timing of RT in LUNG-SORT (3.7 months after EGFR inhibitor start) was similar to that in the present study, there was a wide range of RT timing (IQR, 1–6 months) in LUNG-SORT, which could have implications on RT planning and toxicities. Another key difference between the two studies is the approach to brain metastases. In LUNG-SORT, patients with up to three residual brain metastases at the 4-week time-point were designated as having cranial oligoresidual disease and received RT to all brain lesions. Conversely, in the present trial consolidative cranial RT was specifically discouraged and was not administered to any patients. It is difficult to determine whether one approach is superior. In LUNG-SORT, four of 26 patients with progression had intracranial progression. In the present study, three of 12 patients with progression had intracranial progression. The current study also features 70% longer median follow up (36 months vs 21 months) than LUNG-SORT, thereby providing further evidence of the feasibility and potential benefit of osimertinib plus consolidative RT for advanced *EGFR* mutant NSCLC.

Hypothetically, the relatively late RT timing in this trial may occur after development of early resistance and subclinical, micrometastatic progression (thereby missing a window to eradicate resistant clones), although clinical response and minimal residual disease assessment patterns in this patient population suggest that those events generally occur months later.^15^^,^^20^ Further studies are needed to determine optimal RT timing in this patient population.

In the present study, osimertinib plus consolidation RT conveyed few safety concerns. Under 15% of patients experienced dysphagia, with all instances being low-grade events. Critically, rates of pneumonitis were under 10%, even though more than half of patients received RT to the lung. Potential explanations for this favourable toxicity profile, which differs considerably from other published experience, include patient selection, RT timing and dose selection, avoidance of concomitant systemic therapy administration, and differences in study population susceptibility to both EGFR TKI-associated and radiation pneumonitis. In two retrospective studies of concurrent osimertinib and thoracic RT, more than half of patients experienced grade ≥2 pneumonitis.^21^^,^^22^ As retrospective reports, these studies may have included patients who were generally less fit and/or had worse pulmonary function than patients eligible and enrolled in prospective clinical trials. Furthermore, RT was generally administered at the time of progression and simultaneously with ongoing osimertinib. In a phase 3 trial of first-generation EGFR inhibitor icotinib with concurrent thoracic RT (60 Gy in 30 daily fractions, started within 14 days of icotinib), over 30% of patients had pneumonitis (5% grade ≥3).^19^ In that study, early RT (which could be associated with larger tumour volumes and greater exposure of normal tissues), and continuation of EGFR inhibitor therapy throughout RT may have contributed to higher rates of pneumonitis. Even greater pulmonary toxicity was observed in a study of concurrent osimertinib and intensity-modulated thoracic RT, with grade ≥2 pneumonitis exceeding 60%.^22^ Similarly, EGFR inhibition was continued throughout RT in the phase 2 LUNG-SORT trial, in which stereotactic RT was initiated over a broad range (IQR, 1–6 months after EGFR inhibitor was started) and almost one-third of patients had pneumonitis (although only 2% had grade ≥3, similar to the current study).^18^ In addition to withholding osimertinib during thoracic RT and receiving RT at a later time-point (and therefore potentially to smaller tumour volumes), patients in the current study tended to receive lower RT doses (modelled after the NRG LU-002 trial) than in the other trials listed in Table 4. Notably, the most used regimen in this study was 35 Gy in 5 fractions, indicating clinician preference to minimise risk of delayed toxicities such as pneumonitis, bronchial stenosis, and oesophageal stenosis. Although this regimen has a lower biological effective dose (BED) than the 1- and 3-fraction options, we considered this an efficacious option given that the RT on the current trial addressed a lower tumour volume at 8 weeks, compared to higher tumour volumes that would be expected at earlier timepoints. Lastly, population characteristics may also contribute to differences in pneumonitis rates. Many of the trials combining RT and EGFR inhibition were conducted exclusively in Asia, whereas fewer than half of patients in the current study were Asian. Multiple studies have identified substantially higher rates of EGFR TKI-related pneumonitis and radiation pneumonitis in Asian populations.^23^^,^^24^ Reasons for this discrepancy remain unknown and represent a key area for future investigation.

Limitations of the current study include the absence of centralised radiation plan review; lack of correlative endpoints; single-arm, non-randomised design; and limited number of centres. As previously stated, prior to treatment planning, the selection of radiation targets was finalised after the treating clinician had obtained approval from the radiation study chair, largely driven by disease that was localisable on CT imaging. However, the choice of fractionation regimen was left to the discretion of the treating physician, generally guided by an overarching principle to limit toxicity to adjacent normal tissues based on lesion location. We acknowledge that a lack of standardised dose prescription and target selection guidelines (e.g. based on pre-treatment lesion size or organ location) may limit the replicability of our results. However, the enrolment of patients regardless of the number and sites of metastases, selection of sites for consolidative RT after osimertinib initiation, and flexibility of RT planning may render the present trial closer to a real-world experience that may enhance generalisability. Given the overall small sample size, and the more limited cohort of patients who received consolidative RT (n = 32), we were unable to perform subgroup analysis to identify case characteristics associated with prolonged disease control. Although we had originally intended to perform translational studies using blood and tissue at the time of disease progression, due to funding limitations and lack of participation in these protocol procedures (a common challenge in lung cancer clinical trials^25^^,^^26^), we were not able to conduct these analyses. Additionally, at the time of trial design, activation, and enrollment (2018–21), serial assessment of tumour burden and genomic features with technologies such as circulating tumour DNA were not widely available. While exploratory in nature, such information may provide early insights into optimal RT timing, resistance mechanisms, and how the strategy of RT + EGFR-TKI might fit into the growing number of initial and subsequent treatment options for patients with advanced *EGFR* mutant NSCLC.

This study's single-arm design limits the ability to draw definitive causal inferences regarding the benefit of consolidative radiotherapy. Without a control group, the influence of confounding variables cannot be fully accounted for, and the observed outcomes may reflect underlying patient or disease characteristics rather than the effect of the intervention itself. Additionally, while the FLAURA trial provides a potential reference point, direct comparisons are not appropriate due to substantial differences in baseline characteristics, treatment settings, and follow-up durations. Although propensity score matching represents an established method for reducing confounding bias in observational studies by balancing covariates and improving comparability, we did not perform such an analysis due to the lack of access to individual patient-level data from external cohorts (such as FLAURA) and the heterogeneity of efficacy assessment in institutional patient cohorts treated off protocol. We therefore recognise that our findings should be interpreted as preliminary and hypothesis-generating. To strengthen the evidence base, ongoing randomised controlled trials such as NORTHSTAR (NCT03410043), in which patients with advanced *EGFR* mutant NSCLC are randomised to osimertinib with or without local consolidative therapy (RT ± surgery), will be essential. The importance of such confirmatory trials was reiterated by the negative results of the NRG LU-002 study of consolidative RT (in which *EGFR* mutant NSCLC was excluded).^13^

In conclusion, this phase 2 trial took a real-world, potentially generalisable approach to the incorporation of consolidative RT and osimertinib therapy for advanced *EGFR* mutant NSCLC by not restricting enrolment according to sites or number of metastases, permitting a range of RT plans, delivering RT several weeks after systemic therapy initiation, and encouraging observation of non-progressing brain metastases. The regimen was both well-tolerated, with notably low rates of pulmonary toxicity, and potentially effective, with PFS exceeding 30 months. These findings are especially timely given ongoing shifts in the treatment of *EGFR* mutant NSCLC, with combination MET and EGFR inhibition (osimertinib plus lazertinib) demonstrating improved PFS and OS compared to osimertinib alone. However, this newer regimen features both greater complexity (infusional as well as oral therapy, required venous thromboembolism prophylaxis) and toxicity (principally infusion reactions, more frequent and severe dermatologic effects, and edema) than does osimertinib monotherapy, which remains among preferred regimens in expert guidelines.^27^ Compared to this multi-agent regimen, osimertinib plus consolidative RT is arguably both straightforward and well-tolerated. If the favourable outcomes in the present study are replicated in ongoing randomised trials, osimertinib and consolidative RT could present a reasonable alternative to other intensified first-line regimens for this population.

## Contributors

SR, PI, DEG: conceptualisation; SS, SR, SZ, CA, AG: data curation; SS, SR, PI, TAM, JED, YZ, KDW, SMC, AA, AR, EM, MK, and DEG: investigation; SZ, CA, AG: formal analysis and visualisation; SS and DEG: project administration; DEG: funding acquisition; SS, SR, and DEG: writing (original manuscript); all authors: writing (review and editing). SS and DEG accessed and verified the underlying data.

## Data sharing statement

Selected deidentified participant data and the redacted protocol are available from the corresponding author on reasonable request.

## Declaration of interests

PI reports grants or contracts from Incyte (to institution); and consulting fees from Johnson & Johnson, AstraZeneca, Pfizer, and Novocure. JED reports leadership or fiduciary role in other board for Takeda, Catalyst, and Regeneron. YZ reports grants or contracts from the UT Lung Specialized Program of Research Excellence (SPORE), the UT Southwestern President Research Council, the UT Southwestern Clinical and Translational Science Award (NIH CTSA KL2 scholar award), American Society of Clinical Oncology, American Cancer Society, and AstraZeneca (all to institution). KDW reports grants or contracts from Elekta; consulting fees from AstraZeneca, Sanofi, and Amgen; patents planned, issued, or pending for KRAS inhibitors, SRC inhibitors, and stereotactic body frame; leadership or fiduciary role in other board for the National Comprehensive Cancer Network (lung cancer screening); stock or stock options in Vibliome, Vellorum, and Stabilix; and serving as co-founder of Stabilix. SMC reports grants or contracts from the U.S. Food and Drug Administration (FDA) and Merck (both to institution); and leadership or fiduciary role in other board for the American Society of Clinical Oncology (ASCO) and Eastern Cooperative Oncology Group-American College of Radiology Imaging Network (ECOG-ACRIN). AR reports payment or honoraria from Omni Oncology and Oncohost; and stock or stock options in Merck, Cigna, and Bristol Myers Squibb. EM reports consulting fees from Abbvie, Bristol Myers Squibb, Merck, AstraZeneca, Sanofi, Daiichi Sankyo, and Johnson & Johnson; support for attending meetings and/or travel from Johnson & Johnson; and participation on a data safety monitoring board for Abbvie, Bristol Myers Squibb, Merck, AstraZeneca, Sanofi, Daiichi Sankyo, and Johnson & Johnson. DEG reports grants or contracts from AstraZeneca, BerGenBio, Karyopharm, and Novocure (all to institution); royalties from Oxford University Press; consulting fees from Abbvie, AstraZeneca, Catalyst Pharmaceuticals, Daiichi Sankyo, Elevation Oncology, Janssen Scientific Affairs, Jazz Pharmaceuticals, Regeneron Pharmaceuticals, and Sanofi; U.S. patents planned, issued, or pending (11,747,345; 17/045,482, 18/504,868, 63/386,387, 63/382,972, and 63/382,257); participation on a data and safety monitoring board for Daiichi Sankyo; leadership or fiduciary role in other board for OncoSeer Diagnostics, Inc. (co-founder and Chief Medical Officer); and stock shares in Gilead. All other authors declare no competing interests.

## Appendix A Supplementary data

Supplemental Table S1

Full protocol

SBRT + EGFR_ECM Cover Letter
