# Supplementary material for: Osimertinib plus consolidative radiotherapy for advanced EGFR mutant non–small cell lung cancer: a multicentre, single-arm, phase 2 trial
Source: eClinicalMedicine. 2025 Aug 26;87:103435. doi: 10.1016/j.eclinm.2025.103435 (PMC12496182; doi:10.1016/j.eclinm.2025.103435)
Supplement: Supplemental Table S1 [file mmc1.docx]

**Supplemental Table 1.** RT regimens at consolidation and at first progression.

| **Radiation sites and Dose (Gy)/total fractions** | **Total count (N)** |
| --- | --- |
| *Consolidation* | |
| RT regimen (Total dose (Gy)/number of fractions)  Thorax  35/5  34/5  30/3  45/15  24/1  30/5  other  Bone  30/3  25/5  34/5  27/3  35/5  other  Liver, 30/3  Adrenal, 35/5 | 31  7  5  5  4  4  3  3  13  4  2  2  2  1  2  1  1 |
| *First progression* | |
| Bone  20/1*  24/6  30/5  Lung  24/1  Mediastinum/hilum  40/5  45/15  Brain  25/5  27/3  24/1 | 6  4  1  1  2  2  2  1  1  3  1  1  1 |
| Supraclavicular nodes, 35/5  Liver, 60/5 | 1  1 |

*single patient was treated to 4 sites
