# Supplementary material for: Osimertinib plus consolidative radiotherapy for advanced EGFR mutant non–small cell lung cancer: a multicentre, single-arm, phase 2 trial
Source: eClinicalMedicine. 2025 Aug 26;87:103435. doi: 10.1016/j.eclinm.2025.103435 (PMC12496182; doi:10.1016/j.eclinm.2025.103435)
Supplement: Full protocol [file mmc2.zip › Full protocol.docm]

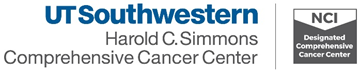


SCCC-02518

Phase II trial of Osimertinib in combination with Stereotactic Ablative Radiation (SABR) in *EGFR* mutant advanced non-small cell lung cancer (NSCLC)

**Principal investigator: Medical oncology co-chair**

Sawsan Rashdan, MD David Gerber, MD

UT Southwestern Medical Center-Oncology UT Southwestern Medical Center-Oncology

5323 Harry Hines Boulevard, Dallas, TX 75390 5323 Harry Hines Boulevard, Dallas, TX 75390

Telephone: (214) 648-4180.Fax: (214) 648-1955 Telephone: (214) 648-4180.Fax: (214) 648-1955

Email: Sawsan.rashdan@utsouthwestern.edu Email: [David.Gerber@UTSouthwestern.edu](mailto:David.Gerber@UTSouthwestern.edu)

**Radiation Oncology co-chair**

Puneeth Iyengar, MD

UT Southwestern Medical Center-Oncology

5323 Harry Hines Boulevard, Dallas, TX 75390

Telephone: (214) 648-4180.Fax: (214) 648-1955

Email: [Puneeth.Iyengar@UTSouthwestern.edu](mailto:Puneeth.Iyengar@UTSouthwestern.edu)

**Biostatistician:**

Chul Ahn, PhD

University of Texas, Southwestern Medical Center

Department of Clinical Science

5323 Harry Hines Boulevard, Dallas, TX 75390

Phone:(214) 648-7438. Fax: (214) 648-5120

Email: [Hong.Zhu@UTSouthwestern.edu](mailto:Hong.Zhu@UTSouthwestern.edu)

**Study Drug:** Osimertinib

**IND: 139456**

**Funding Source**: Astrazeneca

NCT Number: 03667820

**Protocol Version 1.0 09 JAN 2018**

**2.0 31 JUL 2018**

**3.0 07 JAN 2019**

**4.0 01 OCT 2019**

**5.0 19 MAY 2020**

**6.0 03 NOV 2021**

**UT Southwestern Medical Center (UTSW)**

**Harold C. Simmons Cancer Center**

**Attn: Clinical Research Office**

**5323 Harry Hines Blvd. MC 9179**

**Dallas, Texas 75390-9179**

**Signature Page**

The signature below constitutes the approval of this protocol and the attachments, and provides the necessary assurances that this trial will be conducted according to all stipulations of the protocol, including all statements regarding confidentiality, and according to local legal and regulatory requirements and applicable U.S. federal regulations and ICH guidelines.

Phase II trial of Osimertinib in combination with Stereotactic Ablative Radiation (SABR) in *EGFR* mutant advanced non-small cell lung cancer (NSCLC)

**Protocol Version6.0 03 NOV 2021**

**Principal Investigator (PI) Name: _____________________________**

**PI Signature: _____________________________**

**Date: ____________________**

TABLE OF CONTENTS

LIST OF ABBREVIATIONS……………………………………………………………………………………..1

[STUDY SUMMARY 3](#_Toc18673579)

[Protocol Schema 4](#_Toc18673580)

[1 Background and Rationale 4](#_Toc18673581)

[1.1 Overview of lung cancer pathology, epidemiology and current treatment 4](#_Toc18673582)

[1.1.1 Epidemiology of lung cancer targetable molecular alterations 4](#_Toc18673583)

[1.1.2 Treatment for *EGFR* mutant (EGFRm) NSCLC 4](#_Toc18673584)

[1.1.3 Combination of targeted therapy with stereotactic radiation 5](#_Toc18673585)

[1.1.4 Patterns of failure in non-small cell lung cancer 6](#_Toc18673586)

[1.1.5 Concept 6](#_Toc18673587)

[1.2 Introduction to investigational treatment(s) and other study treatment(s) 6](#_Toc18673588)

[1.2.1 Overview of osimertinib (osimertinib): 6](#_Toc18673589)

[1.2.2 Rationale of continuing systemic therapy after oligoprogression 9](#_Toc18673590)

[1.2.3 Rationale of adding *s*tereotactic ablative radiation therapy after targeted therapy 10](#_Toc18673591)

[1.3 Study rationale and purpose 11](#_Toc18673592)

[1.4 Rationale for the protocol design 11](#_Toc18673593)

[1.5 Rationale for dose and regimen selection 11](#_Toc18673594)

[2 Hypothesis and Study Objectives 11](#_Toc18673595)

[2.1 Hypothesis 11](#_Toc18673596)

[2.2 Objectives: 11](#_Toc18673597)

[2.3 Description of protocol design 13](#_Toc18673598)

[2.3.1 Treatment phase and duration of treatment 13](#_Toc18673599)

[2.3.2 Definition of end of the Study 14](#_Toc18673600)

[3 Subject eligibility 14](#_Toc18673605)

[3.1 Patient population 14](#_Toc18673606)

[3.2 Inclusion criteria 14](#_Toc18673607)

[3.3 Exclusion criteria 15](#_Toc18673608)

[4 Treatment Plan 18](#_Toc18673610)

[4.1 Treatment Dosage and Administration 18](#_Toc18673611)

[Patients who have difficulty swallowing solids: 18](#_Toc18673612)

[4.2 Labeling 19](#_Toc18673613)

[4.3 Storage: 19](#_Toc18673614)

[4.4 Treatment duration 19](#_Toc18673615)

[4.5 Dose escalation guidelines 19](#_Toc18673616)

[4.6 Dose modifications 19](#_Toc18673617)

[4.7 Dose Interventions 20](#_Toc18673618)

[4.7.1 Pulmonary Symptoms 20](#_Toc18673619)

[4.7.2 QTc Prolongation 20](#_Toc18673620)

[4.7.3 Keratitis 20](#_Toc18673621)

[4.7.4 Changes in cardiac contractility 20](#_Toc18673622)

[4.7.4 Liver abnormalities 21](#_Toc18673623)

[4.8 Concomitant medications 21](#_Toc18673624)

[4.9 Patient numbering and treatment assignment 21](#_Toc18673625)

[4.9.1 Patient numbering 21](#_Toc18673626)

[4.9.2 Treatment assignment 21](#_Toc18673627)

[4.10 Study drug compliance 22](#_Toc18673630)

[5 Visit schedule and assessments 22](#_Toc18673631)

[5.1 Screening/Baseline Procedures 22](#_Toc18673632)

[5.1.1 Informed Consent 22](#_Toc18673633)

[5.1.2 Medical history 22](#_Toc18673634)

[5.1.3 Demographics 22](#_Toc18673635)

[5.1.4 Review subject eligibility criteria 22](#_Toc18673636)

[5.1.5 Review previous and concomitant medications 23](#_Toc18673637)

[5.1.6 Physical exam including vital signs, height and weight 23](#_Toc18673638)

[5.1.7 Performance status 23](#_Toc18673639)

[5.1.8 Adverse event assessment 23](#_Toc18673640)

[5.1.9 Hematology 23](#_Toc18673641)

[5.1.10 Serum chemistries and coagulation 23](#_Toc18673642)

[5.1.11 Urinalysis 23](#_Toc18673643)

[5.1.12 ECG 23](#_Toc18673644)

[5.1.13 Echocardiogram: 23](#_Toc18673649)

[5.1.14 Pregnancy test (for females of child bearing potential) 24](#_Toc18673650)

[5.1.15 Tumor assessment 24](#_Toc18673651)

[5.1.16 Follow-up after Treatment 24](#_Toc18673652)

[5.2 Protocol flow and visit schedule 24](#_Toc18673653)

[5.2.1 Screening/Baseline 28](#_Toc18673654)

[5.2.2 Treatment period 29](#_Toc18673655)

[5.2.3 End of treatment visit including study completion and premature withdrawal 29](#_Toc18673656)

[5.3 Assessment types 30](#_Toc18673657)

[5.3.1 Efficacy assessments 30](#_Toc18673658)

[5.3.2 Safety and tolerability assessments 31](#_Toc18673659)

[6 Measurement of Effect 33](#_Toc18673664)

[6.1 Antitumor Effect 33](#_Toc18673665)

[6.1.1 Definitions 33](#_Toc18673666)

[6.1.2 Disease Parameters 33](#_Toc18673667)

[6.1.3 Methods for Evaluation of Measurable Disease 34](#_Toc18673668)

[6.1.4 Response Criteria 35](#_Toc18673669)

[6.1.5 Duration of Response 36](#_Toc18673671)

[6.1.6 Overall Survival 37](#_Toc18673675)

[6.1.7 Progression-Free Survival 37](#_Toc18673676)

[6.1.8 Time to Subsequent SABR 37](#_Toc18673677)

[6.1.9 Time until discontinuation of osimertinib or Death 37](#_Toc18673678)

[7 Adverse events 37](#_Toc18673679)

[7.1 Definition of adverse events 37](#_Toc18673680)

[7.2 Definitions of serious adverse event 37](#_Toc18673681)

[7.3 Contraindications: 38](#_Toc18673682)

[7.4 Special Warnings and Precautions for Use: 38](#_Toc18673683)

[7.4.1 Interstitial Lung Disease/Pneumonitis: 38](#_Toc18673684)

[7.4.2 QTc Interval Prolongation: 38](#_Toc18673685)

[7.4.3 Cardiomyopathy 38](#_Toc18673686)

[7.4.4 Embryo-Fetal Toxicity: 39](#_Toc18673687)

[7.5 Adverse Reactions 39](#_Toc18673688)

[Table 7.6.1, Adverse Reactions (>10% for all NCI CTCAE* Grades or >2% for Grades 3-4) in AURA1 and AURA2 39](#_Toc18673689)

[Table 7.6.2, Common Laboratory Abnormalities (>20% for all NCI CTCAE Grades) in AURA1 and AURA2. 40](#_Toc18673690)

[7.6 Adverse Event Monitoring 41](#_Toc18673691)

[Unanticipated Problems Involving Risks to Subjects or Others (UPIRSOs) 42](#_Toc18673692)

[7.6.1 Disease progression 43](#_Toc18673693)

[7.6.2 New cancers 43](#_Toc18673694)

[7.6.3 Lack of efficacy 43](#_Toc18673695)

[7.6.4 Deaths 43](#_Toc18673696)

[7.7 Reporting adverse events 43](#_Toc18673697)

[7.7.1 Unanticipated Problems Involving Risks to Subjects or Others (UPIRSOs) 46](#_Toc18673698)

[7.7.2 Steps to Determine If an Adverse Event Requires Expedited Reporting 46](#_Toc18673699)

[7.7.3 Adverse events based on signs and symptoms 47](#_Toc18673700)

[7.7.4 Adverse events based on examinations and tests 47](#_Toc18673701)

[7.8 Warnings and precautions 47](#_Toc18673702)

[8 Investigational product and other Treatments 47](#_Toc18673703)

[8.1 Identity of investigational product(s) 47](#_Toc18673704)

[8.1.1 Agent: 47](#_Toc18673705)

[8.1.2 Return and Retention of Study Drug 48](#_Toc18673706)

[8.1.3 Study Drug compliance 48](#_Toc18673707)

[8.2 Therapy: Stereotactic Ablation Body Radiation (SABR) 48](#_Toc18673708)

[8.2.1 SABR DOSE AND TECHNIQUES 48](#_Toc18673709)

[Technical Factors 49](#_Toc18673710)

[8.2.2 49](#_Toc18673711)

[8.2.3 Dose Verification at Treatment 49](#_Toc18673712)

[8.2.4 Treatment Platforms 49](#_Toc18673713)

[*8.2.5* Simulation/Image Guidance 50](#_Toc18673714)

[*8.3* Treatment Planning/Target volumes 50](#_Toc18673715)

[*8.3.1* Image Acquisition 50](#_Toc18673716)

[*8.3.2* Target Volumes 50](#_Toc18673717)

[*8.4* Dosimetry 51](#_Toc18673718)

[*8.4.1* 3D-Conformal Planning 51](#_Toc18673719)

[*8.4.2* Intensity Modulated Radiation Therapy (IMRT) 51](#_Toc18673720)

[*8.4.3* Dose Calculations 51](#_Toc18673721)

[8.4.4 Normal Tissue Dose Constraints 52](#_Toc18673722)

[8.5 Intracranial Stereotactic Radiosurgery Dose (SRS) and Technique 55](#_Toc18673723)

[8.5.1 SRS Dose 55](#_Toc18673724)

[8.5.2 SRS Technique, Pre-SRS Image acquisition and target planning with Conformality Index 55](#_Toc18673725)

[CI = Rx Isodose Vol / (Target Vol) 56](#_Toc18673726)

[8.5.3 Normal Tissue Dose Constraints 56](#_Toc18673727)

[8.6 Radiation Therapy Quality Assurance 56](#_Toc18673728)

[9 STATISTICAL CONSIDERATIONS 56](#_Toc18673729)

[9.1 Study Design/Study Endpoints 56](#_Toc18673730)

[9.2 Sample Size and Accrual 57](#_Toc18673731)

[9.3 Data Analyses Plans 57](#_Toc18673732)

[9.4 Analysis sets 58](#_Toc18673733)

[9.4.1 Full Analysis Set 58](#_Toc18673734)

[9.4.2 Safety Set 58](#_Toc18673735)

[9.4.3 Patient demographics/other baseline characteristics 58](#_Toc18673736)

[9.4.4 Treatments (osimertinib treatment, concomitant therapies, compliance) 58](#_Toc18673737)

[9.4.5 Handling of missing values/censoring/discontinuations 58](#_Toc18673738)

[9.4.6 Safety objectives 58](#_Toc18673739)

[9.4.7 Analysis set and grouping for the analyses 58](#_Toc18673740)

[9.4.8 Adverse events (AEs) 58](#_Toc18673741)

[9.4.9 Other safety data 59](#_Toc18673742)

[9.4.10 Treatment phase and duration of treatment 59](#_Toc18673743)

[9.5 Definition of end of the Study 59](#_Toc18673744)

[9.6 Early Termination 59](#_Toc18673745)

[9.7 Data confidentiality 59](#_Toc18673746)

[10 Ethical considerations and administrative procedures 59](#_Toc18673747)

[10.1 Regulatory and ethical compliance 59](#_Toc18673748)

[10.2 Informed consent procedures 60](#_Toc18673749)

[10.3 Discontinuation of the study 60](#_Toc18673750)

[10.4 Publication of the study and results 60](#_Toc18673751)

[10.4.1 Communication and Publication of Clinical Trial Results 60](#_Toc18673752)

[10.5 Study documentation, record keeping and retention of documents 60](#_Toc18673753)

[10.6 Confidentiality of study documents and patient records 60](#_Toc18673754)

[10.7 Audits and inspections 60](#_Toc18673755)

[11 STUDY MANAGEMENT 60](#_Toc18673756)

[11.1 Conflict of Interest (COI) 60](#_Toc18673757)

[11.2 Institutional Review Board (IRB) Approval and Consent 60](#_Toc18673758)

[11.3 Registration Procedures 61](#_Toc18673759)

[11.4 Data Management and Monitoring/Auditing 62](#_Toc18673760)

[11.5 Adherence to the Protocol 63](#_Toc18673762)

[11.6 Amendments to the Protocol 65](#_Toc18673781)

[11.7 Record Retention 65](#_Toc18673782)

[11.8 Obligations of Investigators 65](#_Toc18673783)

[11.8.1 Source data 65](#_Toc18673784)

[11.8.2 Study agreements 66](#_Toc18673785)

[12 References (available upon request) 67](#_Toc18673786)

[13 Appendices 69](#_Toc18673787)

LIST OF ABBREVIATIONS

AE Adverse Event

*ALK* Anaplastic lymphoma kinase

ALT Alanine aminotransferase/glutamic pyruvic transaminase/GPT

ALC Absolute Lymphocyte Count

ASCO American Society of Clinical Oncology

AST Aspartate aminotransferase/glutamic oxaloacetic transaminase/GOT

BUN Blood Urea Nitrogen

BCRP multidrug transporter ABCG2

b.i.d. bis in diem/twice a day

CBC Complete Blood Count

CBR Clinical benefit rate

CMP Comprehensive Metabolic Panel

CNS Central nervous system

CR Complete Response

CT Computed Tomography

CTCAE Common Terminology Criteria for Adverse Events

CrCl creatinine clearance

CRO Contract Research Organization

DLT Dose Limiting Toxicity

DOT Disease Oriented Team

DSMB Data and Safety Monitoring Board

DSMC Data and Safety Monitoring Committee

DoR Duration of response

DS&E Drug Safety and Epidemiology

ECG Electrocardiogram

ECOG Eastern Cooperative Oncology Group

EIAED enzyme inducing anti-epileptic medication

EGFR epidermal growth factor receptor

EGFRm EGFR mutated

EOT End of treatment

FAS Full Analysis Set

FDA Food and Drug Administration

GCP Good Clinical Practice

GI Gastric intestinal

GTV Gross Tumor Volume

hERG human Ether-à-go-go-Related Gene

IUD intrauterine device

IUS intrauterine system

ICH International Conference on Harmonization

IEC Independent Ethics Committee

IHC immunohistochemistry

IIT Investigator-Initiated Trial

IRB Institutional Review Board

LLN Lower limit of normal

LLNA local lymph node assay

MRI Magnetic Resonance Imaging

MUGA Multigated acquisition

MTD Maximum Tolerated Dose

NCI National Cancer Institute

NOS Not otherwise specified

NSCLC non-small cell lung cancer

o.d. omnia die/once a day

ORR Objective response rate

OS Overall survival

OTC over-the counter

QD once daily

pCR Pathologic Complete Response

PD Progressive disease

PET Positron Emission Tomography

p.o. per os/by mouth/orally

PFS Progression free survival

PHI Protected Health Information

PK Pharmacokinetics

PPI proton pump inhibitors

PR Partial response

Racc accumulation ratio

RAP The Report and Analysis Plan (RAP) is a regulatory document which provides evidence of preplanned analyses

RDE Recommended dose for expansion

REB Research Ethics Board

RECIST Response Evaluation Criteria in Solid Tumors

RR Response Rate

SABR Stereotactic Ablative Radiation

SAE Serious Adverse Event

SCCC Simmons Comprehensive Cancer Center

SD Stable DiseaseSLD Sum of the Longest Diameter

SOP Standard Operating Procedure

TDI time-dependent inhibition

TKI tyrosine kinase inhibitors

ULN upper limit of normal

UV Ultraviolet radiation

VATS Video-assisted thoracic surgery

VEGF vascular endothelial growth factor

WBC White Blood Cells

WHO World Health Organization

# STUDY SUMMARY

| Title | Phase II trial of Osimertinib in combination with Stereotactic Ablative Radiation (SABR) in *EGFR* mutant advanced non-small cell lung cancer (NSCLC) |
| --- | --- |
| Short Title | Osimertinib and SABR in *EGFR* mutant NSCLC |
| Protocol Number | SCCC-02518 |
| Phase | II |
| Methodology | Open label, single arm |
| Study Duration | 4 years |
| Study Center(s) | Multicenter |
| Objectives | Primary endpoint: To determine if osimertinib and SABR can increase PFS in patients with *EGFR*m NSCLC.  Secondary endpoints: Safety/toxicity, duration of response (DoR), objective response rate (ORR), overall survival (OS), time to subsequent SABR or death, time to osimertinib discontinuation or death.  Exploratory endpoints: predictive and pharmacodynamic biomarkers, molecular mechanisms of resistance |
| Number of Subjects | 43 |
| Diagnosis and Main Inclusion Criteria | 1. EGFR mutant (exon 19 and 21) advanced NSCLC 2. No prior treatment with EGFR inhibitor therapy 3. Adequate bone marrow, renal, hepatic, function 4. Performance status ECOG 0-2 |
| Study Product(s), Dose, Route, Regimen | Osimertinib 80 mg orally daily |
| Duration of administration | Until progression or intolerance of therapy. |
| Reference therapy | Expansion cohort of the AURA trial presented at ESMO LBA1_PR, 2016 (S. Ramalingam) |
| Statistical Methodology | Kaplan Meier Methods will be used to estimate progression-free survival and overall survival. A sample size of 42 patients achieves 80% power to detect the difference between the null hypothesis of median PFS of 19.3 months and the alternative hypothesis of median PFS of 30 months at one-sided significance level of 0.1. We assume the patient accrual period of 36 months and the follow-up period of 12 months, and assume that the survival time follows an exponential distribution. |

Protocol schema

Induction Therapy

Radiation Therapy

Continuation Therapy

Osimertinib

80 mg Daily

Eligibility

- EGFR mutant advanced NSCLC
- No prior treatment with EGFR inhibitor therapy
- Adequate bone marrow, renal, hepatic, function
- Performance status ECOG 0-2

Reimage at

8 Weeks

SABR to persisting Lesions

Continue Osimertinib until progression or intolerance of therapy

**additional SABR to progressive lesions will be allowed after initial radiation therapy if clinically indicated*

# Background and Rationale

## Overview of lung cancer pathology, epidemiology and current treatment

### Epidemiology of lung cancer targetable molecular alterations

Lung cancer is the most common cancer worldwide. There are over 1.5 million cases annually, leading to more than 1 million deaths every year [1]. In the US, 160,000 deaths occur from lung cancer annually [2].

Lung cancer is classified broadly into small cell lung cancer and non-small cell lung cancer (NSCLC). NSCLC is further classified into squamous cell cancer, adenocarcinoma, large cell, and NSCLC NOS. Treatment for each classification varies, as well as their clinical behavior and molecular/genetic profile.

Non-squamous NSCLC, in particular adenocarcinoima, has been found to harbor mutations that can be targeted with specific anti-cancer agents. These have dramatically improved the outlook for the minority of patients with tumors that do harbor an actionable mutation. These drugs are much better tolerated than traditional chemotherapies, with fewer side effects and also easier to take. However, the duration of response to these targeted therapies is limited by the development of clinical resistance.

### Treatment for *EGFR* mutant (EGFRm) NSCLC

Patients with lung adenocarcinoma now routinely have molecular/genetic testing performed on their tumor. EGFRm NSCLC accounts for 10%–15% of NSCLC patients in Europe and the United States, it is more frequently observed in women and non-smokers. In Asia, however, the incidence of EGFR mutations ranged from 22-62%. Although this was more common in non-smokers, the incidence in smokers was still high at 37%. [3]. Due to the large number of lung cancer cases annually, this small percentage in North America represents thousands of patients every year in the US. *EGFR* mutations are rarely found in squamous cell cancer.

| Most of the *EGFR* mutations have been found in between exon 18 and exon 21. The most common mutations are in-frame deletions in exon 19, and the L858R point mutation in exon 21. Together, the axon 19 and 21 mutations account for about 90% of all EGFRms [4] |
| --- |
|  |

EGFR TKIs has dramatically transformed the treatment of EGFR mutated lung cancer with an objective response rate (ORR) of ~70%–80% and a median progression-free survival (PFS) of 10–12 months. [5]

To date, there are three generations of TKIs. Erlotinib and gefitinib are the first-generation agents; afatinib, dacomitinib, and neratinib are the second-generation agents; and osimertinib, rociletinib, HM61713, and ASP8273 are the recently developed third-generation agents.

Osimertinib is FDA approved for patients with advanced *EGFR* mutant, T790M mutation positive NSCLC, whose disease has progressed on or after EGFR TKI therapy. It has also showed significant activity when used as a first-line treatment for patients with *EGF*R mutant NSCLC. A Phase I/II, open-label, multicenter study recently reported by Ramalingam et al[6], patients were treated with either osimertinib 80 mg orally once daily (n=30) or 160 mg orally once daily (n=30). Objective response rate was 77% (80-mg dosing, 67%; 160-mg dosing: 87%). PFS was 19.3 months with the 160 mg dosing and was not reached with the 80 mg dosing. It is expected that osimertinib will outperform first generation EGFR inhibitors in the currently ongoing phase III trial in which it is compared with erlotinib and gefitinib in the first-line setting (Clinicaltrials.gov NCT02296125)

Despite these encouraging results, it is clear that—as the case for all other molecularly targeted therapies for lung cancer—patients treated with osimertinib inevitably progress. In some instances of so-termed oligo-progression, patients may be successfully treated with local therapy (most commonly stereotactic radiation) and continue to receive the same systemic therapy for several more months. This approach is most effective for intracranial progression, which is more likely to represent pharmacologic limitation (ie, drug delivery) than molecular evolution[7, 8]. Indeed, secondary resistance mutations to osimertinib have already been identified [9].

### Combination of targeted therapy with stereotactic radiation

Stereotactic ablative body radiation (SABR) is a technique of delivering precisely targeted radiation to tumors while minimizing the dose given to adjacent normal tissue. Large doses of radiation are delivered in a small of number of treatments. Due to the radio-biological response to this type of radiation, there can be dramatic tumor responses leading to the ablative terminology.

SABR has an increasing role in advanced NSCLC, as demonstrated by a Phase II trial of targeted therapy with SABR [10]. This trial was conducted at UT Southwestern and enrolled 24 patients with advanced NSCLC that had progressed on standard chemotherapy. These patients were treated with the erlotinib plus stereotactic ablative radiation to sites of visible disease. As a historical control, in a previous trial, patients with advanced NSCLC who had progressed after one line of chemotherapy and were then treated with erlotinib had a progression free survival (PFS) of 2.3 months and overall survival (OS) of 6.7 months [11]. In our trial, combining SABR with erlotinib trial prolonged the median PFS to 14.7 months and median OS to 20.7 months.


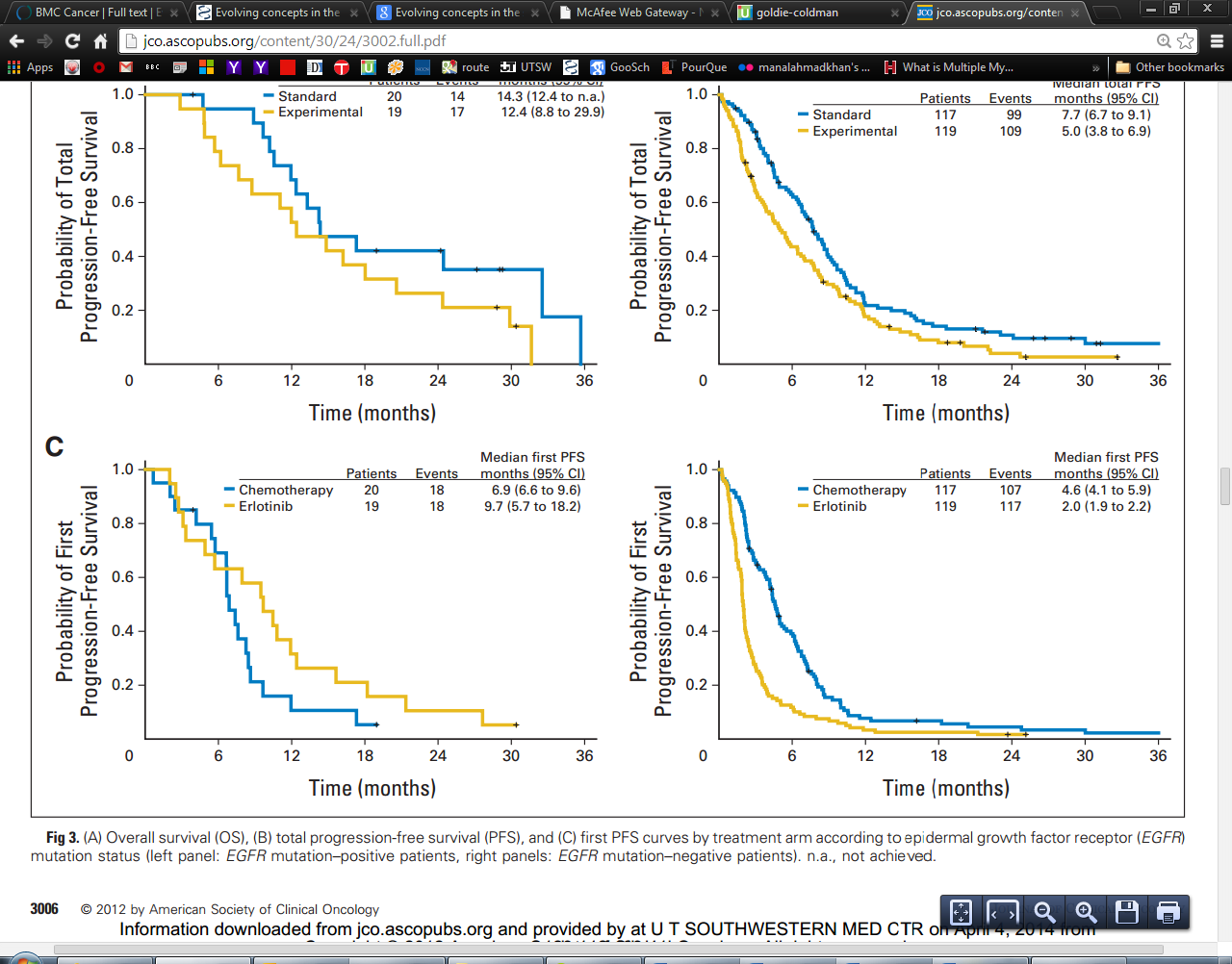


**B**

**A**

**Fig 3.** Kaplan-Meier curves for Progression Free-Survival.

Panel A: Patients with *EGFR* mutations, PFS with erlotinib=9.7 months. Panel B: Patients with *EGFR* wild-type, PFS with erlotinib =2 months.

This combination of SABR with targeted therapy greatly prolonged the duration of time that targeted therapy was beneficial for patients. The combination was well tolerated, and completion of study enrollment in a period of several months demonstrated our site’s ability to carry out such trials efficiently and successfully.

Accordingly, we propose to administer local therapy in conjunction with osimertinib not at the time of progression—when radiographically evident changes likely represent only a fraction of biological disease worsening—but at the time of anticipated best response. Specifically, we believe that tumor sites that remain radiographically evident after 8-10 weeks of osimertinib monotherapy may harbor weakly sensitive or resistant clones. This is suggested by (1) the observation that disease progression usually occurs at sites of known disease; and (2) the Goldie-Coldman hypothesis of tumor resistance, [12] which suggests that any detectable tumor will contain some resistant clones. According to this hypothesis, the probability of resistance developing in a tumor depends on (a) the number of cancer cells and (b) the mutation rate of the constituent cells [13]. Therefore, targeting known and visible disease may delay progression that requires a change in systemic therapy.

### Patterns of failure in non-small cell lung cancer

Reasons for discontinuing anti-cancer therapy include ineffective control of existing lesions or the development of new lesions. The patterns of failure in non-small lung cancer were evaluated in 64 patients with advanced non-small lung cancer [14]. 64% of patients in this study demonstrated progression in already known sites of disease. This suggests that enhanced control of existing lesions will result in prolongation of the benefit from systemic therapy.

### Concept

The proposed study is designed to prolong the clinical benefit of EGFR targeted therapy in patients with EGFR mutant lung cancer. This will evaluate the combination of two well-tolerated therapies, osimertinib and SABR. This dual therapy is expected to be more effective than either alone in destroying sensitive tumor cells and also in preventing the development of resistant cells.

Based on current studies, patients derive benefit from first generation EGFR inhibitors for less than a year before they need to be switched to another treatment, usually osimertinib if the tumor harbors the *EGFR* T790M mutation. Osimertinib alone is active therapy. However, resistance and progression will develop as with all EGFR TKIs. Tumors that remain after 8 weeks of osimertinib monotherapy may harbor weakly sensitive or resistant clones. While on systemic therapy, progression usually occurs at sites of known disease. Therefore, targeting known and visible disease may delay progression that requires a change in systemic therapy.

This study will treat lesions that persist after 8 weeks of osimertinib with stereotactic ablative radiation. Limited progression after that period will also be considered for repeat SABR if possible, rather than discontinuing the osimertinib. This accurately reflects current clinical practice and recommendations from expert groups such as the NCCN, which recommend continuing targeted therapy after progression in some cases.

## Introduction to investigational treatment(s) and other study treatment(s)

### Overview of osimertinib (osimertinib):

Osimertinib is an oral, potent, irreversible EGFR TKI selective for sensitizing (EGFRm) and T790M resistance mutations with a significant selectivity margin against wild-type *EGFR*. As a result, osimertinib can effectively block EGFR signaling both in *EGFR* single mutant cells with activating *EGFR* mutations and in double mutant cells bearing the resistance T790M mutation. Osimertinib is currently FDA approved as a treatment option in Patients with advanced T790M positive NSCLC who have previously failed an EGFR-TKI. It is under investigation in patients with advanced EGFRm NSCLC who are treatment naïve; and In combination with novel agents for patients with EGFR TKI resistant NSCLC.

#### Non-clinical experience

##### **Pharmacology**

Osimertinib is a kinase inhibitor for oral administration. The molecular formula for osimertinib mesylate is C28H33N7O2•CH4O3S, and the molecular weight is 596 g/mol. The chemical name is N-(2-{2-dimethylaminoethyl-methylamino}-4-methoxy-5-{[4-(1-methylindol-3-yl)pyrimidin-2yl]amino}phenyl)prop-2-enamide mesylate salt. Osimertinib binds irreversibly, via the C797 amino acid covalent bond, and potently inhibits EGFR phosphorylation to certain mutant forms of EGFR (L858R, exon 19 deletion, and double mutants containing T790M). But it is less potent at inhibiting phosphorylation of EGFR in wild-type cell lines. IC50 (50% inhibiting concentration) values for exon 19 deletion and L858R/T790M are 12.92 nM and 11.44 nM, respectively, while IC50 for wild-type EGFR is ~493.8 nM. [16]

This inhibits the downstream pathways such as RAS/ RAF/MAPK and PI3K/AKT, that regulate DNA synthesis and proliferation. There are two main products of the metabolism of osimertinib: AZ5104 and AZ7550. AZ7550 has similar profile to the parent molecule, while AZ5104 exhibits less selectivity against WT EGFR compared with osimertinib[16]

The tablets contain 40 or 80 mg of osimertinib, equivalent to 47.7 and 95.4 mg of osimertinib mesylate, respectively. Inactive ingredients in the tablet core are mannitol, microcrystalline cellulose, low-substituted hydroxpropyl cellulose and sodium stearyl fumarate. The tablet coating consists of polyvinyl alcohol, titanium dioxide, macrogol 3350, talc, ferric oxide yellow, ferric oxide red and ferric oxide black.

##### **Pharmacodynamics:**

In vitro studies showed that osimertinib potently inhibits cellular growth in both EGFRm+ and EGFRm+ /T790M mutant cell line, with 9 folds lower activity against wild-type EGFR lines. It also inhibits the activity of ERBB2, ERBB3, ERBBR4, ACK1, and BLK at clinically relevant concentrations. In vivo studies showed that osimertinib caused significant and sustained tumor shrinkage in tumor xenograft and transgenic mouse models harboring activating EGFR mutations and EGFR T790M after 14 days of 2.5 mg/kg/day dose.[17]

Osimertinib showed higher activity in patients with detectable plasma levels of EGFR T790M DNA compared with patients without detectable plasma (85% vs 33%) when used as the second-line treatment for patients with EGFRm+ NSCLC.[18]

##### **Pharmacokinetics**

The area under the plasma concentration-time curve (AUC) and maximal plasma concentration (Cmax) of osimertinib increased dose proportionally over 20 to 240 mg dose range (i.e., 0.25 to 3 times the recommended dosage) after oral administration and exhibited linear pharmacokinetics (PK). Administration of osimertinib orally once daily resulted in approximately 3-fold accumulation with steady state exposures achieved after 15 days of dosing. At steady state, the Cmax to Cmin (minimal concentration) ratio was 1.6-fold.

##### Absorption The median time to Cmax of osimertinib was 6 hours (range 3-24 hours). Following administration of a 20 mg osimertinib tablets with a high-fat, high-calorie meal (containing approximately 58 grams of fat and 1000 calories), the Cmax and AUC of osimertinib increased by 14% and 19% respectively, compared to fasting conditions.

##### Distribution The mean volume of distribution at steady-state (Vss/F) of osimertinib was 986 L. Plasma protein binding of osimertinib is likely high based on its physiochemical properties.

##### Elimination Osimertinib plasma concentrations decreased with time and a population estimated mean half-life of osimertinib was 48 hours, and oral clearance (CL/F) was 14.2 (L/h).

##### Metabolism The main metabolic pathways of osimertinib were oxidation (predominantly CYP3A) and dealkylation in vitro. Two pharmacologically active metabolites (AZ7550 and AZ5104) have been identified in the plasma after osimertinib oral administration. The geometric mean exposure (AUC) of each metabolite (AZ5104 and AZ7550) was approximately 10% of the exposure of osimertinib at steady-state.

##### Excretion Osimertinib is primarily eliminated in the feces (68%) and to a lesser extent in the urine (14%). Unchanged osimertinib accounted for approximately 2% of the elimination.

##### Specific Populations No clinically significant differences in the pharmacokinetics of osimertinib were observed based on age, sex, ethnicity, body weight, smoking status, mild (CLcr 60-89 mL/min) or moderate (CLcr 30-59 mL/min) renal impairment, or mild hepatic impairment (total bilirubin <ULN and AST between 1 to 1.5x ULN or total bilirubin between 1.0 to 1.5 times ULN and any AST ). There are no data on the pharmacokinetics of osimertinib in patients with severe renal impairment (CLcr less than 30 mL/min) or with moderate to severe hepatic impairment (moderate: total bilirubin between 1.5 to 3.0 times ULN and any AST, and severe: total bilirubin between 3.0-10 times ULN and any AST).

##### **PK drug-drug interactions**

As CYP3A4/5 is the principal P450 protein responsible for the metabolism of osimertinib, AZ5104 and AZ7550, co-administration with a potent CYP3A4 inhibitor or inducer may affect their exposure. Osimertinib, AZ5104 and AZ7550 are substrates for P-gp and BCRP but are not substrates for OATP1B1 and OATP1B3.

No restrictions on co-adminstration of osimertinib with CYP3A4 inhibitors or substrates are required; however, it is recommended that potent inducers of CYP3A4be only co-administered with osimertinib if no other alternative exists and patients be closely monitored for reduction in efficacy.

The likelihood of a clinically meaningful DDI with osimertinib and drug transporters suggests that an 80 mg dose of osimertinib may result in a DDI via inhibition of intestinal BCRP. Also, co-administering osimertinib with a P-Glycoprotein (Pgp) substrate increased the exposure of the substrate compared with administering it alone. It is recommended that patients taking concomitant medications where the disposition is dependent upon breast cancer resistance protein (BCRP) and/or P-glycoprotien (P-gp) which have a narrow therapeutic index (eg, rosuvastatin) should be closely monitored for signs of changed tolerability as a result of increased exposure of the concomitant medication whilst receiving osimertinib.

##### **Safety pharmacology and toxicology**

The toxicology of osimertinib has been evaluated in repeat dose oral toxicity studies of up to 6 months in rats and 9 months in dogs (1 month studies included an assessment of recovery), genetic toxicology studies, reproductive toxicology studies (assessing male and female fertility and effects on embryofetal development and early post-natal survival/growth in rats) and in an in vitro phototoxicity assay. Lung findings identified in the 3 month rat study were further characterised by electron microscopy.

The nonclinical toxicology profile of osimertinib largely reflects the pharmacological action of this compound. Osimertinib is a potent inhibitor of the mutant forms of the EGFR, but also shows less potent inhibition of the wild-type EGFR and other members of the ErbB receptor family (eg.HER2), which are expressed in normal tissues (Yano et al 2003; Wieduwilt and Moasser, 2008). Osimertinib has demonstrated a margin of selectivity over these closely related receptors in in vitro pharmacology models; however, it is not unexpected that toxicities associated with inhibition of these receptors would be seen when doses up to the maximum tolerated are evaluated in the toxicology studies.

The principal histopathological findings observed in the repeat dose toxicity studies comprised atrophic, inflammatory and/or degenerative changes affecting the epithelia of the eye (cornea), GI tract (including tongue), skin and female reproductive tract with secondary changes in the spleen, bone marrow and lymph nodes. Similar findings have been reported in animal studies with other EGFR TKIs including gefitinib, erlotinib, afatinib (a pan irreversible inhibitor of EGFR, HER2 and HER4) and lapatinib (dual EGFR and HER2 selective inhibitor). There were additional histopathological findings in the male reproductive tract, male mammary gland, lymph nodes, Harderian gland, lung, bone marrow, thymus, lacrimal gland, tarsal gland, kidney, liver and adrenal gland. These target organ findings are briefly discussed below:

#### Clinical efficacy safety and tolerability:

Please refer to the osimertinib Investigator’s Brochure (IB) for details of the clinical programme for osimertinib.

In the phase 1 dose escalation study of osimertinib (D5160C00001, AURA), no dose-limiting toxicities were reported in any of the dose escalation cohorts (20, 40, 80, 160, and 240mg) and a non-tolerated dose has not been defined. Based on the totality of the safety, pharmacokinetic and preliminary efficacy data, 80 mg once daily was selected as the recommended phase II dose.

Data from the ongoing phase I AURA study (D5160C00001) in patients with T790M positive NSCLC who were previously treated with EGFR TKI, have achieved promising efficacy with osimertinib; 54.2% 95% CI (40.8%, 67.3%) of subjects achieved a response, 91.5% 95% CI (81.3%, 97.2%) achieved disease control, medium duration of response of 12.4 months 95% CI (8.3, NC) and median PFS based on 38% maturity of data was 13.5 months 95% CI (8.3, NC), as assessed by blinded independent central review[15].  Promising evidence of efficacy has also observed in patients with EGFRm treatment naïve NSCLC treated with osimertinib as first line EGFR TKI[6] .

Across the clinical development programme, osimertinib has demonstrated a manageable safety profile [15]

### Rationale of continuing systemic therapy after oligoprogression

This continuation of erlotinib beyond progression is recommended by the NCCN guidelines for the following benefits; delayed symptoms, reduction in tumor size and FDG avidity on PET [19, 20]. In a Japanese study, 64 out of 186 EGFR mutated patients treated with an EGFR TKI with radiological PD on first- or second-line treatment with an EGFR TKI either continued TKI or chemotherapy upon PD. Survival advantage (32.2 months versus 23.0 months) was achieved with the continuation of EGFR TKI treatment versus the switch to chemotherapy, respectively [21-23] These data suggest that for a carefully selected group of patients, continuing *EGFR* inhibitors even in the setting of limited progressive disease results in clinically meaningful benefit.

However the optimal therapy to combine with targeted therapy beyond progression is ill-defined as adding systemic chemotherapy is often too toxic.

The decision to continue osimertinib at the time of oligoprogression will be at the discretion of treating oncologist in line with standard clinical practice. If, in the opinion of the treating oncologist, the patient would benefit from continued osimertinib this will be allowed even after documented oligoprogression.

### Rationale of adding *s*tereotactic ablative radiation therapy after targeted therapy

Stereotactic ablative body radiation (SABR) is a technique of delivering precisely targeted radiation to tumors while minimizing the dose given to adjacent normal tissue. Large doses of radiation are delivered in a small of number of treatments. Due to the radio-biological response to this type of radiation, there can be dramatic tumor responses leading to the ablative terminology.

In this study, SABR will be added as a cytoreductive agent after major cancer cell killing is achieved with osimertinib administered for 8 weeks. Following the Goldie-Coldman hypothesis, it seems plausible that the resulting cancer debulking will then result in a smaller chance of resistant clones developing according to the Goldie-Coldman hypothesis. Accordingly, this approach may prolong the overall duration of osimertinib efficacy, delaying the development of clinically significant resistance that necessitates a switch from osimertinib to another systemic therapy.

In the trial, subsequent post-SABR limited progression will be considered for repeat SABR if clinically feasible, with ongoing osimertinib administration. This strategy accurately reflects current clinical practice and recommendations from the national authorities such as the NCCN, which recommend continuing targeted therapy after progression in some cases.

*At study entry:*

Patients on this trial who have lesions requiring radiation prior to initiation of systemic therapy in the view of the treating physician will be allowed to get such radiation. This will not limit their trial participation, though the lesion will be noted as having been radiated.

*After 8 weeks of osimertinib monotherapy:*

Lesions that persist after osimertinib monotherapy will receive stereotactic radiation. These include lesions that may have progressed during osimertinib monotherapy alone. As long as no change in systemic therapy is indicated, these patients will continue on the trial. Up to 6 sites or organs will receive stereotactic radiation, at the discretion of the treating physician. All sites of gross tumor (typically defined by CT and PET) should be treated for patients to continue on study.

*Subsequent areas of progression:*

As long as the study participant is on osimertinib as the sole systemic therapy, further stereotactic radiation may be delivered to up to 6 sites or organs at a time. Lesions in close proximity to one another amenable to treatment with a single SABR field are considered a single site. When osimertinib is no longer the systemic agent or when new progressive sites cannot feasibly be treated with stereotactic radiation (e.g., malignant pleural effusion or site previously irradiated), study participation will end. The primary endpoint of progression-free survival will be calculated from the start of treatment to the first episode of progression.

*Osimertinib during stereotactic radiation:*

Currently there are limited data about the safety of concurrent osimertinib and stereotactic radiation, though studies are ongoing. In this trial, osimertinib may be held prior to, during and after stereotactic radiation per the treating investigator’s discretion. The total expected duration of the stereotactic radiation is expected to range between 1-5 treatments, generally not exceeding 21 days from first to last radiation therapy.

*CNS metastases:*

Stereotactic radiation to CNS lesions that require therapy will be allowed. At study entry the presence of CNS lesions or their treatment with radiation will not constitute a restriction to study participation. Subsequently, progression in the CNS will not terminate study participation, and patients may receive stereotactic radiation to those areas.

Evaluation of CNS metastases will consist of an MRI or contrast enhanced CT performed at baseline and then as clinically indicated, but at least every 3 months. Patients without CNS involvement will not be required to have mandatory CNS imaging. All patients with new symptoms suspicious of CNS involvement will have urgent CNS imaging performed.

## Study rationale and purpose

Patients with *EGFR* mutant non-small cell lung cancer will receive the current optimal therapy with osimertinib. After 8 weeks of targeted therapy, there will likely be some persisting lesions that would not have completely regressed. These persisting lesions would likely consist of cells that are less sensitive to targeted therapy. From the data summarized above [14], these persisting lesions are most to subsequently develop resistance and demonstrate progression.

To delay the onset of clinical progression, lesions that persist after 8 weeks of osimertinib therapy and are amenable to stereotactic ablative radiation will be radiated. Osimertinib may be held prior to, during and after stereotactic radiation per the treating investigator’s discretion. After radiation, all patients will continue osimertinib therapy. If subsequently there is any evidence of progression, there will be an assessment of whether a repeat course of radiation is feasible. If it is feasible to repeat SABR to sites of progression, this will be performed and osimertinib resumed. If SABR is not possible, then a change in systemic therapy will be required.

## Rationale for the protocol design

The purpose of the trial is to determine if the addition of SABR to osimertinib can prolong the clinical benefit of targeted therapy. This will be an open-label, non-randomized, single arm trial in *EGFR* mutant lung adenocarcinoma. The protocol will also report the safety of treating with SABR and osimertinib, though both are widely used in clinical practice.

## Rationale for dose and regimen selection

In the AURA phase I study, the recommended dose for the expansion cohorts and future trials was determined to be 80 mg daily (QD). The dose-escalation cohort with 31 patients and the dose-expansion cohort with 222 patients who received oral osimertinib at five dosages of 20 mg/d, 40 mg/d, 80 mg/d, 160 mg/d, or 240 mg/d. No dose-limiting toxic effects were observed in the dose escalation cohort (31 patients). Tumor response rates were similar across all osimertinib dose levels, with increasing toxicities at the 160 mg and 240 mg daily doses; thus, a dose of 80 mg daily was adopted for future studies.

The most common adverse events were diarrhea (47% of patients), rash (40%), nausea (22%), and decreased appetite (21%). Adverse events of diarrhea and rash increased in frequency in a dose-dependent manner. Any event of grade 3 or higher was observed in 32% of the patients. Adverse events leading to dose reduction or drug discount

inuation were observed in 7% and 6%, respectively, of all patients. Serious adverse events were observed in 22% of the patients;

The package insert for osimertinib recommends a starting dose of 80 mg daily. There are recommendations for holding/discontinuation for toxicity however no dosing adjustments are needed for renal/hepatic impairment.

# Hypothesis and Study Objectives

## Hypothesis

Our proposed study is designed to prolong the clinical benefit of osimertinib therapy in patients with *EGFR* mutant lung cancer. We will evaluate the combination of two well-tolerated therapies: osimertinib and SABR. This dual therapy is expected to be more effective than either alone in destroying sensitive tumor cells and also in preventing the development of resistant cells. Published experience suggests that median PFS for patients who have previously been treated with EGFR TKI is less with osimertinib than those who have never been treated with an EGFR TKI (10.1 months vs 19.3 months). In this study, the patients enrolled will have no prior treatment with EGFR inhibitors.

## Objectives:

Primary Objective

- The primary objective of this study will be to determine the efficacy of osimertinib plus SABR in patients with EGFR mutant lung cancer. The measure for this outcome will be: Progression-Free Survival (PFS); defined as time from first dose of osimertinib to date of objective progression (as determined by the investigator according to RECIST 1.1) or death (in the absence of progression). Patients will still be followed for progression even if they stop due to toxicity or start a subsequent therapy prior to progression.

There may be progression in a specific localized region that is amenable to stereotactic radiation. If all existing lesions that enlarge and any new lesions that develop can still be effectively treated with stereotactic radiation, patients will be evaluated for repeat SABR therapy and osimertinib resumed. This will be at the recommendation of the treating physician according to standard practice. This will be defined as Time to 2^nd^ SABR therapy and will be one of the study secondary endpoints. Similarly, if 3^rd^ round of SABR can be safely provided with subsequent resumption of osimertinib, that will be defined as the Time to 3^rd^ SABR therapy.

Secondary Objectives

Secondary objectives include the following:

| Objective | Endpoint | Definition of endpoint |
| --- | --- | --- |
| To determine the impact of osimertinib plus SABR on survival | Overall survival | Defined as: Time from the date of initiation of osimertinib until death of any cause. |
| To determine the impact of osimertinib plus SABR on length of response | Duration of response (DoR) | Defined as: The time from the date of first documented response, (that is subsequently confirmed) until date of documented progression or death in the absence of disease progression, the end of response should coincide with the date of progression or death from any cause used for the PFS endpoint. The time of the initial response will be defined as the latest of the dates contributing towards the first visit response of PR or CR. If the response is not confirmed, it will not be included. If a subject does not progress following a response, then their duration of response will use the PFS censoring time. |
| To determine the impact of osimertinib plus SABR on the length of time until next therapy needed. | Time to subsequent SABR (2^nd^, 3^rd^, etc) or death. | Defined as the time from the date of first SABR, until the first date of documented progression that is amenable to SABR. |
| To determine the impact of osimertinib plus SABR on tumor response. | Objective response rate (ORR) | Defined as the proportion of patients with measurable disease who had a response per the definitions stated in section (6.1.4) After receiving at least one cycle of therapy. |
| To determine the impact of osimertinib plus SABR on the duration of time while on osimertinib. | Time until discontinuation of osimertinib or death | Time to treatment discontinuation or death (TDT) is defined as the time from first dose to the earlier of the date of treatment discontinuation or death. Any patient not known to have died at the time of analysis and not known to have discontinued study treatment will be censored based on the last recorded date on which the patient was known to be alive. |
| Demonstrate safety of osimertinib followed by SABR | Safety and tolerability | Safety: Defined as the risk to patients by using osimertinib plus SABR  Tolerability: Defined as and the degree to which overt adverse events of the osimertinib plus SABR can be tolerated. |

Translational and exploratory objectives

Biopsy of progressive sites will be encouraged per standard of care practice. After 8 weeks of osimertinib, treatment will be transiently held and SABR will be delivered to persisting lesions. These patients will then resume osimertinib monotherapy and continue clinical and radiographic monitoring. During this time if progressive lesions are found after resumption of osimertinib they will be biopsied if feasible. These research biopsies will be performed after informed consent is obtained and only if biopsies can be done safely. If a biopsy is performed as part of the patient’s standard of care, we will request tissue from the standard of care biopsy if available, rather then perform an additional biopsy for research. Archival tissue will only be collected for patients who consent to tissue collection at time of progression. Research biopsies will not be required for patients on this study. We will collect blood at the time of progression for circulating tumor DNA (ctDNA) mutation analysis. For circulating tumor DNA (cfDNA) mutation analysis, 32 ml of whole blood (8 X 4 tubes) [Approximately 2 table spoon] will be drawn in yellow top 8.5 ml ACD tubes (Soln A) at end of treatment. Plasma will be processed and frozen for analysis in batch. Buffy coat will be frozen also.

This trial will offer the opportunity to improve our understanding of the molecular changes that occur during the process of osimertinib resistance. Collected tissue and blood samples will be tested for molecular mechanisms of resistance, which may include C797S resistant mutation[24], MET and HER2 amplifications [25], and small cell transformation, all of which have been reported as resistance mechanisms to osimertinib .

## Description of protocol design

This is an, open-label, single arm protocol designed to evaluate the activity of targeted therapy and SABR in EGFR+ lung adenocarcinoma. Study participants will have to be eligible for both radiation and targeted therapy with osimertinib.

### Treatment phase and duration of treatment

Patients eligible for treatment will receive 80 mg daily of osimertinib as part of a 28-day cycle. The first dose of each cycle will be administered after evaluation at the study center for the first 6 cycles. Following 6 cycles, visits will be extended to every 2 cycles. Following 1 year of participation, study visits will be completed every 3 cycles unless the treating physician prefers more frequent visits. Patients will remain on study and take osimertinib until there is evidence of disease progression by RECIST 1.1, unacceptable toxicity, withdrawal of consent, or discontinuation of the trial for any other reason. In case of oligoprogression amenable to repeat courses of SABR, the treating oncologist may resume osimertinib if it is indicated per standard clinical practice. Patients must continue to be followed for safety and efficacy assessments as per the schedule of assessments.

At 8 weeks from initiation of osimertinib, persisting lesions will be treated with a course of SABR. Osimertinib will be held per the treating physician’s discretion. Regular surveillance will follow and any new or enlarging lesions that develop will be assessed for repeat SABR.

### Definition of end of the Study

### Study treatment will end when all 43 patients have evidence of disease progression by RECIST not amenable to SABR requiring discontinuation of osimertinib, unacceptable toxicity, withdrawal of consent, or discontinuation of the trial for any other reason. After completion of study treatment all patients will be seen within 30 days for an end of therapy evaluation. This will include a safety assessment for AE’s SAE’s. Any unused medication will be returned. After that patients will be followed for survival every 3 months until death. Data will be collected every 3 months until all 43 patients have deceased. Early Termination

Treatment on protocol may be terminated early if the Principal investigator or institution assess that the safety of the enrolled subjects will be compromised by continuation of the trial. The procedure followed will be that of the premature withdrawal patient and any patients on treatment will be seen as soon as possible.

# Subject eligibility

Eligibility waivers are not recommended; however, if warranted, prior approvals are required per Section 11.5.1. Subjects must meet all inclusion and exclusion criteria to be registered to the study. Study treatment may not begin until a subject is registered. Once registered, a subject is still required to meet all inclusion and exclusion criteria on the first day of treatment, prior to treatment.

## Patient population

Subjects for this study will be selected from those with *EGFR* mutant NSCLC. EGFR mutations may be demonstrated by standard, clinically accepted methods, including direct gene sequencing, PCR, and NextGen sequencing.

Patients will be informed about their potential eligibility status for this trial. None of these patients will be given any therapy or undergo any study related assessments/procedures until they have successfully screened and enrolled on to the therapeutic portion of the study.

Patients enrolled to the therapeutic portion of the study will have histologically proven lung adenocarcinoma with EGFR mutation detected by polymerase chain reaction or gene sequencing assays as described above.

## Inclusion criteria

Eligibility waivers are not permitted. Subjects must meet all of the inclusion and none of the exclusion criteria to be registered to the study. Study treatment may not begin until a subject is registered.

1. Written informed consent in accordance with federal, local, and institutional guidelines. The patient must provide informed consent prior to the first screening procedure. However, the Investigator should not repeat procedures that are performed as part of standard of care (SOC), if they are within the screening window and are done prior to signing the ICF.
2. Age > 18 years
3. Advanced *EGFR* exon 19 or 21 mutant NSCLC, not amenable to curative surgery or radiotherapy. EGFR mutations may be demonstrated by standard, clinically accepted methods, including direct gene sequencing, PCR, and NextGen sequencing.
4. World Health Organization (WHO)/Eastern Cooperative Oncology Group (ECOG) performance status 0-2.
5. Patients must have a life expectancy ≥ 12 weeks.
6. Females should be using adequate contraceptive measures, should not be breast feeding and must have a negative pregnancy test prior to start of dosing if of child-bearing potential or must have evidence of non-child-bearing potential by fulfilling one of the following criteria at screening:

- Post-menopausal defined as aged more than 50 years and amenorrheic for at least 12 months following cessation of all exogenous hormonal treatments
- Women under 50 years old would be consider postmenopausal if they have been amenorrheic for 12 months or more following cessation of exogenous hormonal treatments and with LH and FSH levels in the post-menopausal range for the institution
- Documentation of irreversible surgical sterilisation by hysterectomy, bilateral oophorectomy or bilateral salpingectomy but not tubal ligation

1. Male patients should be willing to use barrier contraception (see Restrictions, Section 3.3)
2. Patient is willing and able to comply with the protocol for the duration of the study including undergoing treatment and scheduled visits and examinations including follow up.
3. At least one lesion, not previously irradiated, that can be accurately assessed at baseline with computed tomography (CT) or magnetic resonance imaging (MRI) and which is suitable for accurate repeated measurements.
4. Adequate bone marrow reserve or organ function as demonstrated by any of the following laboratory values:

- Absolute neutrophil count >1.5 x 10^9^/L
- Platelet count >100 x 10^9^/L
- Haemoglobin >9.0 g/dL (transfusion is permitted to achieve Hgb ≥9.0 g/dL)
- Alanine aminotransferase <2.5 times the upper limit of normal (ULN) if no demonstrable liver metastases or <5 times ULN in the presence of liver metastases
- Aspartate aminotransferase <2.5 times ULN if no demonstrable liver metastases or <5 times ULN in the presence of liver metastases
- Total bilirubin <1.5 times ULN if no liver metastases or <3 times ULN in the presence of documented Gilbert’s Syndrome (unconjugated hyperbilirubinemia) or liver metastases
- Serum Creatinine <1.5 times ULN concurrent with creatinine clearance >50 ml/min (measured or calculated by Cockcroft and Gault equation); confirmation of creatinine clearance is only required when creatinine is >1.5 times ULN.

## Exclusion criteria

1. Involvement in the planning and/or conduct of the study (applies to both sponsor staff and/or staff at the study site)
2. Previous treatment with osimertinib or any EGFR TKI.
3. Previous treatment with immunotherapy or any check point inhibitors.
4. Treatment with an investigational drug within five half-lives of the compound or 3 months, whichever is greater
5. Patients currently receiving (or unable to stop use prior to receiving the first dose of study treatment) medications or herbal supplements known to be potent inducers of CYP3A4 (at least 3 week prior) (Appendix A).  All patients must try to avoid concomitant use of any medications, herbal supplements and/or ingestion of foods with known inducer effects on CYP3A4.
6. Any unresolved toxicities from prior therapy greater than Common Terminology Criteria for Adverse Events (CTCAE) grade 1 at the time of starting study treatment with the exception of alopecia and grade 2, prior platinum-therapy related neuropathy.
7. Any evidence of severe or uncontrolled systemic diseases, including uncontrolled hypertension and active bleeding diatheses, which in the investigator’s opinion makes it undesirable for the patient to participate in the trial or which would jeopardise compliance with the protocol, or active infection including hepatitis B, hepatitis C and human immunodeficiency virus (HIV). Screening for chronic conditions is not required.
8. Patients with spinal cord compression, symptomatic and unstable brain metastases except for those patients who have completed definitive therapy, and have had a stable neurological status for at least 2 weeks after completion of definitive therapy. Patients may be on corticosteroids to control brain metastases if they have been on a stable dose for 2 weeks (14 days) prior to the start of study treatment and are clinically asymptomatic
9. Patients with metastatic disease involving the esophagus, stomach, intestines or mesenteric lymph nodes.
10. Past medical history of ILD, drug-induced ILD, radiation pneumonitis requiring steroid treatment, or any evidence of clinically active ILD
11. Any of the following cardiac criteria:
12. Mean resting corrected QT interval (QTc using Fredericia’s formula) > 470 msec
13. Any clinically important abnormalities in rhythm, conduction or morphology of resting ECG (e.g., complete left bundle branch block, third degree heart block, second degree heart block)
14. Any factors that increase the risk of QTc prolongation or risk of arrhythmic events such as heart failure, electrolyte abnormalities (including: Serum/plasma potassium < LLN; Serum/plasma magnesium < LLN; Serum/plasma calcium < LLN) , congenital long QT syndrome, family history of long QT syndrome or unexplained sudden death under 40 years of age in first degree relatives or any concomitant medication known to prolong the QT interval and cause Torsades de Pointes
15. Refractory nausea and vomiting, chronic gastrointestinal diseases, inability to swallow the formulated product or previous significant bowel resection that would preclude adequate absorption of osimertinib
16. History of hypersensitivity to osimertinib (or drugs with a similar chemical structure or class to osimertinib) or any excipients of these agents
17. Males and females of reproductive potential who are not using an effective method of birth control and females who are pregnant or breastfeeding or have a positive (urine or serum) pregnancy test prior to study entry
18. Judgment by the Investigator that the patient should not participate in the study if the patient is unlikely to comply with study procedures, restrictions and requirements
19. Previous allogeneic bone marrow transplant.
20. Inadequate bone marrow reserve or organ function as demonstrated by any of the following laboratory values:

- Absolute neutrophil count <1.5 x 10^9^/L
- Platelet count <100 x 10^9^/L
- Haemoglobin <90 g/L
- Alanine aminotransferase >2.5 times the upper limit of normal (ULN) if no demonstrable liver metastases or >5 times ULN in the presence of liver metastases
- Aspartate aminotransferase >2.5 times ULN if no demonstrable liver metastases or >5 times ULN in the presence of liver metastases
- Total bilirubin >1.5 times ULN if no liver metastases or >3 times ULN in the presence of documented Gilbert’s Syndrome (unconjugated hyperbilirubinaemia) or liver metastases
- Creatinine >1.5 times ULN concurrent with creatinine clearance <50 ml/min (measured or calculated by Cockcroft and Gault equation); confirmation of creatinine clearance is only required when creatinine is >1.5 times ULN.

.

**Restrictions:**

The following restrictions apply while the patient is receiving osimertinib and for the specified times before and after:

1. Females of child-bearing potential should use reliable methods of contraception from the time of screening (at least 2 weeks prior to first dose) until 6 weeks after discontinuing osimertinib.  Acceptable methods of contraception include total and true sexual abstinence, hormonal contraceptives that are not prone to drug-drug interactions (IUS Levonorgestrel Intra Uterine System (Mirena), Medroxyprogesterone injections (Depo-Provera)), copper-banded intra-uterine devices, and vasectomized partner.  All hormonal methods of contraception should be used in combination with the use of a condom by their male sexual partner for intercourse.
2. Male patients should be asked to use barrier contraceptives (i.e., by use of condoms) during sex with women of childbearing potential, including pregnant women, during the trial and for a washout period of 4 months. Patients should not father a child for 4 months after completion of osimertinib treatment. Patients should refrain from donating sperm from the start of dosing until 4 months after discontinuing osimertinib treatment. If male patients wish to father children they should be advised to arrange for freezing of sperm samples prior to the start of osimertinib treatment.
3. If medically feasible, patients taking regular medication, with the exception of potent inducers of CYP3A4 (see above), should be maintained on it throughout the access program period (30 days post-last dose). Patients taking concomitant medications whose disposition is dependent upon breast cancer resistance protein (BCRP) and/or P-glycoprotein (P-gp) which have a narrow therapeutic index should be closely monitored for signs of changed tolerability as a result of increased exposure of the concomitant medication whilst receiving osimertinib. Guidance on medications to avoid, medications that require close monitoring and on washout periods is provided (see Appendix A).

Patients taking rosuvastatin should have creatine phosphokinase levels monitored (due to BCRP-mediated increase in exposure). If the patient experiences any potentially relevant AEs suggestive of muscle toxicity including unexplained muscle pain, tenderness, or weakness, particularly if accompanied by malaise or fever, rosuvastatin must be stopped and any appropriate further management should be taken.

# Treatment Plan

## Treatment Dosage and Administration

For this protocol, the term “treatment” refers to osimertinib. The dose and schedule are listed under table 4-1.

Each cycle of therapy will be 28 days long. A completed cycle will be 28 days of once daily continuous treatment. Day 1 will be the first day that osimertinib is taken, and the last day of a completed treatment cycle will be day 28.

Table 4-1:

| Investigational product | Dosage form and strength | Manufacturer |
| --- | --- | --- |
| osimertinib | 40mg Tablets  80mg Tablets | AstraZeneca |

AstraZeneca will supply osimertinib as tablets for oral administration as a single daily dose of 80 mg. osimertinib will usually be supplied as either bulk or unlabelled bottles for ISS/ESR studies.

Additional information about the Investigational product may be found in the Investigators’ Brochure.

Administration guidance

Osimertinib is administered as 80 mg once daily. Osimertinib may be taken without regard to food.

Doses should be taken approximately 24 hours apart at the same time point each day. Doses should not be missed. If a patient misses taking a scheduled dose, within a window of 12 hours, it is acceptable to take the dose. If it is more than 12 hours after the dose time, the missed dose should not be taken, and patients should be instructed to take the next dose at the next scheduled time. If a patient vomits after taking their osimertinib, they should not make up for this dose, but should take the next scheduled dose.

The dose of 80 mg osimertinib daily can be reduced to 40 mg osimertinib once daily under circumstances described in Section [4.7]. Further dose reductions are not possible. Once a dose has been reduced, it should not be re-escalated at future cycles.

Patients will self-administer the doses of osimertinib for the cycle. A dosing diary will be given to the patient to record doses and missed doses. The dosing diary will be reviewed by the study team at each cyclefor dosing compliance.

### Patients who have difficulty swallowing solids:

Disperse tablet in 4 tablespoons (approximately 60 mL) of non-carbonated water only. Stir until tablet is completely dispersed and swallow or administer through naso-gastric tube immediately. Do not crush, heat, or ultrasonicate during preparation. Rinse the container with 4 to 8 ounces of water and immediately drink or administer through the naso-gastric tube.

#### Any change from dosing schedule, dose interruptions, or dose reductions should be recorded

## Labeling

Labels will be prepared in accordance with Good Manufacturing Practice (GMP) and local regulatory guidelines. The labels will fulfill GMP Annex 13 requirements for labeling.

## Storage:

All study drugs should be kept in a secure place under appropriate storage conditions. The investigational product label on the bottle and the Investigator Brochure specifies the appropriate storage.

## Treatment duration

Patients will continue osimertinib treatment until they experience any of the following:

-Disease progression not amenable to SABR and necessitating change in systemic therapy determined by the treating physician.

-Unacceptable toxicity that precludes further treatment.

-Start of a new anti-cancer therapy.

-Pregnancy.

-Treatment is discontinued at the discretion of the investigator or patient.

-Lost to follow-up.

-Death.

## Dose escalation guidelines

No doses escalation will be permitted on this study.

## Dose modifications

Dose adjustment for adverse events should be in accordance with the following table:

Table 4.6 Osimertinib dose adjustment information for adverse reactions

| **Target Organ** | **Adverse Reaction^a^** | **Dose Modification** |
| --- | --- | --- |
| Pulmonary | ILD/Pneumonitis | Permanently discontinue osimertinib |
| Cardiac | QTc interval greater than 500 msec on at least 2 separate ECGs | Withhold osimertinib until QTc interval is less than 481 msec or recovery to baseline if baseline QTc is greater than or equal to 481 msec, then restart at a reduced dose (40 mg). |
|  | QTc interval prolongation with signs/symptoms of serious arrhythmia | Permanently discontinue osimertinib |
| Other | Grade 3 or higher adverse reaction | Withhold osimertinib for up to 3 weeks |
|  | If Grade 3 or higher adverse reaction improves to Grade 0-2 after withholding of osimertinib for up to 3 weeks | osimertinib may be restarted at the same dose (80 mg) or a lower dose (40 mg) |
|  | Grade 3 or higher adverse reaction that does not improve to Grade 0-2 after withholding for up to 3 weeks | Permanently discontinue osimertinib |

^a^ Note: The intensity of clinical adverse events graded by the National Cancer Institute (NCI) Common Terminology Criteria for Adverse Events (CTCAE) version 4.0.

If a patient experiences a CTCAE grade 3 or higher and/or unacceptable toxicity (any grade), where the clinician considers the event of concern to be specifically associated with osimertinib (and not attributable to the disease or disease-related processes for which patient is being treated), dosing will be interrupted and supportive therapy administered as required in accordance with local practice/guidelines. Detailed information on the clinical management of events may be found in Appendix B, “Guidance for the Management of Adverse Events in Studies using 80 mg osimertinib.”

Patients with QTc prolongation (i.e., confirmed QTc prolongation to >500 msec absolute on at least 2 separate ECGS should have osimertinib treatment interrupted and regular ECGs performed until resolution to baseline.

If the toxicity resolves or reverts to ≤CTCAE grade 2 within 3 weeks of onset, treatment with osimertinib may be restarted at the same dose (80 mg, daily) or a lower dose (40 mg, daily) using the rules below for dose modifications (Table 1). There will be no individual modifications to dosing schedule in response to toxicity, only potential dose reduction or dose interruption.

If the toxicity does not resolve to ≤CTCAE grade 2 after 3 weeks, then the patient should be withdrawn from the study and observed until resolution of the toxicity.

## Dose Interventions

| Intervention | Osimertinib Dose |
| --- | --- |
| Starting Dose | 80 mg daily |
| Reduced Dose | 40 mg daily |

On resolution of toxicity within 3 weeks:

If an event subsequently requires dose interruption, osimertinib may restart at the same dose or the reduced dose, on resolution/improvement of the event at the discretion of the clinician.

### Pulmonary Symptoms

### If new or worsening pulmonary symptoms (e.g., dyspnea) or radiological abnormality suggestive of interstitial lung disease or pneumonitis is observed, osimertinib should be permanently discontinued. QTc Prolongation

In light of the potential for QT changes associated with osimertinib, electrolyte abnormalities (hypokalaemia, hypomagnesaemia, hypocalcaemia) must be corrected to be within normal ranges prior to first dose and electrolyte levels monitored during study treatment. Patients with QTcF prolongation fulfilling the following criteria (i.e., confirmed QTcF prolongation to > 500 msec absolute should have osimertinib interrupted and regular ECGs performed until resolution to <481 msec or baseline QTcF if baseline QTcF is >481 msec, and then restarted at a reduced dose of 40 mg . If the toxicity does not resolve to < grade 1 within 3 weeks the patient will be permanently withdrawn from osimertinib treatment. For QTc prolongation with signs/symptoms of serious arrhythmia, osimertinib treatment will not be reinitiated.

### Keratitis

Keratitis was reported in 0.7% (n=6) of the 833 patients treated with osimertinib in the AURA studies. Patients presenting with signs and symptoms suggestive of keratitis such as acute or worsening: eye inflammation, lacrimation, light sensitivity, blurred vision, eye pain and/or red eye should be referred promptly to an ophthalmology specialist.

### 4.7.4 Changes in cardiac contractility

Across clinical trials, LVEF decreases greater than or equal to 10% and a drop to less than 50% occurred in 4.0% (26/655) of patients treated with osimertinib who had baseline and at least one follow-up LVEF assessment. Based on the available clinical trial data, a causal relationship between effects on changes in cardiac contractility and osimertinib has not been established. Echocardiogram/MUGA to assess LVEF should be performed at screening in patients with pre-existing cardiac disease that does not meet the criteria for study exclusion but may worsen on treatment with osimertinib. Subsequent assessment of LVEF should be performed as clinically indicated. In patients who develop cardiac signs/symptoms during treatment, cardiac monitoring including LVEF assessment should be considered.

### Liver abnormalities

**NOTE:** In case a subject shows an AST **or** ALT ≥3xULN **or** total bilirubin ≥ 2xULN please refer to Appendix C ‘Actions required in cases of combined increase of Aminotransferase and Total Bilirubin – Hy’s Law’, for further instructions.

### 4.7.5 Erythema Multiforme and Stevens-Johnson syndrome

Case reports of Erythema multiforme (EM) and Stevens-Johnson syndrome (SJS) have been uncommonly and rarely reported, respectively, in association with osimertinib treatment. Before initiating treatment, patients should be advised of signs and symptoms of EM and SJS. If signs and symptoms suggestive of EM develop, close patient monitoring and drug interruption or discontinuation of osimertinib should be considered. If signs and symptoms suggestive of SJS appear, osimertinib should be interrupted or discontinued immediately.

## Concomitant medications

Once enrolled, all patients must try to avoid concomitant use of medications, herbal supplements and/or ingestion of foods that are known to be potent inducers of CYP3A4 whenever feasible, but patients may receive any medication that is clinically indicated for treatment of adverse events. Such drugs must have been discontinued for an appropriate period before they enter screening and for a period of 3 months after the last dose of osimertinib. All concomitant medications should be captured on the CRF. Guidance on medicines to avoid, medications that require close monitoring and on washout periods is provided (see Appendix A).

If medically feasible, patients taking regular medication, with the exception of potent inducers of CYP3A4 (see above), should be maintained on it throughout the study period. Patients taking concomitant medications whose disposition is dependent upon BCRP and/or P-glycoprotein (P-gp) which have a narrow therapeutic index should be closely monitored for signs of changed tolerability as a result of increased exposure of the concomitant medication whilst receiving osimertinib.

Patients taking rosuvastatin should have creatine phosphokinase levels monitored (due to BCRP-mediated increase in exposure). If the patient experiences any potentially relevant AEs suggestive of muscle toxicity including unexplained muscle pain, tenderness, or weakness, particularly if accompanied by malaise or fever, rosuvastatin must be stopped and any appropriate further management should be taken

Guidance on medications to avoid, medications that require close monitoring and on washout periods should be provided (see Appendix A)

Patients taking warfarin should be monitored regularly for changes in prothrombin time or INR.

## Patient numbering and treatment assignment

### Patient numbering

Each patient will be assigned a subject number at the time of screening which shall be the primary identifier for the patient throughout the entire trial. This number will not be reassigned if a patient is not enrolled on to the therapeutic portion of the trial. Once assigned a number, the patient will not be given a new number if enrollment is delayed behind other patients.

### Treatment assignment

Before the first dose of osimertinib, the investigator will confirm that the patient meets all eligibility criteria. The patient will be assigned to the treatment arm and the Investigator or delegate will update the trial record accordingly.

## Study drug compliance

At each visit, the patient’s compliance in study medication according to protocol will be assessed by the investigator and/or study personnel. This information will be entered into the Drug Accountability Form and captured into the source document at each visit, in their medication diary.

# Visit schedule and assessments

## Screening/Baseline Procedures

Assessments performed exclusively to determine eligibility for therapy on this study will be done only after obtaining informed consent. Assessments performed for clinical indications (not exclusively to determine study eligibility) may be used for baseline values even if the studies were done before informed consent was obtained.

All screening procedures must be performed within 30 days prior to registration into the study unless otherwise stated. The screening procedures include:

### Informed Consent

Screening assessments to confirm eligibility will be performed as per the schedule of assessments. Documented Informed consent must be obtained before any study specific procedure will be performed.

For treatment on the trial, the patient must have a documented *EGFR mutation* either by sequencing or assay and meet the remainder of the eligibility criteria.

Re-screening of patients will be allowed, if all entry criteria are met during the re-screening phase time period (-30 days to -1 day).

### Medical history

Complete medical and surgical history, including history of infections. Any ongoing signs/symptoms present during Screening should be assessed and graded per CTCAE, and followed as per section 7

### Demographics

Data to be collected on patient characteristics at screening include:

-Demography (including: date of birth, age, patient initials, gender, childbearing potential,

race and ethnicity, or as allowed by local regulations)

-Lung adenocarcinoma diagnosis and extent of disease, including:

Date of diagnosis

Site of active disease

Prior antineoplastic therapies (medications, radiation, surgeries)

Prior and Concomitant Medications, surgical and medical procedures

All other medications taken within 30 days before the first dose of osimertinib treatment is administered will be noted in the clinical trial medication record and updated on a continual basis if there is new change to the medication.

### Review subject eligibility criteria

According to section 3

### Review previous and concomitant medications

### Physical exam including vital signs, height and weight

A physical examination will be performed prior to receiving first dose of study drug on day 1 of every cycle and at the EoT Visit. The physical exam will include: general appearance, skin, head and neck (including ears, eyes, nose and throat), respiratory, cardiovascular, abdomen, lymph nodes, thyroid, musculo-skeletal (including spine and extremities) and neurological systems. Vital signs (temperature, pulse, respirations, blood pressure, and weight) will be obtained and recorded with any clinically relevant findings notated as such. The COVID-19 pandemic has caused disruption in normal operations. Some deviation from normal standards of care may be experienced during this time and physical exams may be limited to what the provider can observe through telehealth visits. The provider should determine when physical exams in person are clinically indicated.

### Performance status

Performance status evaluated prior to study entry.

### Adverse event assessment

Baseline adverse events will be assessed. See section 7.1 for Adverse Event monitoring and reporting.

### Hematology

Hgb, platelets, white blood cells (WBC), red blood cells (RBC), differential (basophils, eosinophils, lymphocytes, monocytes, neutrophils (% or absolute).

### Serum chemistries and coagulation

Albumin, ALT, AST, calcium, creatinine, total bilirubin, direct bilirubin (only if total bilirubin is ≥ grade 2), blood urea nitrogen (BUN) or urea, magnesium, potassium, sodium, glucose, phosphate (inorganic phosphorus), alkaline phosphatase. Creatinine clearance will be calculated using the serum creatinine value. Coagulation tests will be Prothrombin Time (PT) and Partial Thromboplastin Time. Electrolyte abnormalities (hypokalaemia, hypomagnesaemia, hypocalcaemia) must be corrected to be within normal ranges prior to first dose and electrolyte levels monitored during study treatment

### Urinalysis

Macroscopic panel (dipstick)(color, total bilirubin, blood, glucose, ketones, leukocyte esterase, nitrite, pH, protein, specific gravity, urobilinogen) Microscopic panel (RBC, WBC, casts, crystals, bacteria, epithelial cells)

### ECG

Patients should be monitored for ECG changes at every cycle. ECGs should be reviewed and any abnormalities noted.

Resting 12 lead ECG: Twelve-lead ECG will be performed at the visit indicated in the Study Plan. If there is a clinically significant abnormal ECG finding during the Treatment period, this should be recorded on the AE eCRF, according to standard adverse events collection and reporting processes. A 28-day follow-up assessment will be required if an on-treatment assessment was abnormal at the time of discontinuation of study therapy, to confirm reversibility of the abnormality.

The COVID-19 pandemic has caused disruption in normal operations. ECG may be omitted when telehealth visit are performed. ECGs should be performed during in person physician visits and as clinically indicated if the patient is being seen through telehealth visits.

### Echocardiogram:

An echocardiogram or multigated acquisition scan (MUGA) to assess LVEF will be performed at screening (prior to first dose of osimertinib) only in patients with pre-existing cardiac disease that does not meet the criteria for study exclusion but may worsen on treatment with osimertinib. Subsequent assessment of LVEF should be performed as clinically indicated. The modality of the cardiac function assessments must be consistent within a patient, i.e., if echocardiogram is used for the screening assessment then echocardiogram should also be used for subsequent scans. The patients should also be examined using the same machine and operator whenever possible, and quantitative measurements should be taken. A 28-day follow-up assessment will be required if an on-treatment assessment was abnormal at the time of discontinuation of study therapy, to confirm reversibility of the abnormality.

### Pregnancy test (for females of child bearing potential)

At screening visit, serum pregnancy test will be performed. Following the screening assessment, urine or serum pregnancy tests should be performed.

### Tumor assessment

To be performed using radiographic imaging according to section 5.3.1.

### Follow-up after Treatment

The study team will attempt to collect survival status and new therapy information for patients who are enrolled. Public sources may be searched for survival status information.

## Protocol flow and visit schedule

Table 5-1 lists the protocol schedule and assessments and indicates when particular assessments will be performed with an “X”. Each cycle of therapy will be 28 days long. Screening procedures may overlap with Cycle 1 Day 1 procedures only if all eligibility criteria is met and confirmed prior to dosing. A physician visit is not needed on C1D1 if performed within 7 days prior to starting treatment. In this case, delegated study personnel may assess performance status (WHO) on Cycle 1 Day 1.There will be variation of up to +/- 3 days allowed in visits and assessments. The COVID-19 pandemic has caused disruption in normal operations. Some deviation from normal standards of care may be experienced during this time. Telehealth visits may be performed per the treating investigator’s discretion.

A visit delay due to holiday, inclement weather, dosing hold for palliative radiation, or other unforeseen circumstances will be permitted for up to 7 days. Delays beyond 7 days must be preapproved by the principal investigator. The reason for any cycle delay should be documented in the patient’s research record.

| **Table 5-1**  **Visit evaluation Schedule** | **Protocol**  **Section** | ***EGFR* status** | **Screening / Baseline** | **Day 1 of Cycle 1 (28d) ± 3** | **Day 15 of Cycle 1 (28d) ± 3** | **Day 1 of Cycle 2 (28d) ± 3** | **Day 1 of Subsequent cycles**  **(28d) ± 3** | **End of study treatment (EoT)**  **(at last visit, or up to 30 days from last dose)** | **Long Term Follow up Q3 mo following EOT** |
| --- | --- | --- | --- | --- | --- | --- | --- | --- | --- |
| **Visit Number** |  |  | **1** | **2** | **3** | **4** | **5, 6..** | **Last** |  |
| **Day of cycle** |  |  | **-30 to D1** | **D1** | **D15** | **D29** | **D57, D85..** | **Last** |  |
| Obtain Informed Consent | ICF | X | X |  |  |  |  |  |  |
| Inclusion/exclusion criteria | 3.2 & 3.3 |  | X |  |  |  |  |  |  |
| Documentation of *EGFR* positivity by PCR or sequencing. | 5.1.1 | X | X |  |  |  |  |  |  |
| Diagnosis and extent of cancer | 5.1.2 |  | X |  |  |  |  |  |  |
| Demography | 5.1.3 |  | X |  |  |  |  |  |  |
| Ophthalmologic evaluation ^2^ |  |  | X* |  |  | X* | X* |  |  |
| Relevant medical history/ current medical conditions | 5.1.4 |  | X |  |  |  |  |  |  |
| Prior antineoplastic therapy  (meds, surgery, radiation) | 5.1.5 |  | X |  |  |  |  |  |  |
| Prior/concomitant medications | 5.1.5 |  | X | Continuous | | | | |  |
| Surgical and Medical Procedures | 4.4 |  | X | Continuous | | | | |  |
| Eligibility Screening | 5.1.4 |  | X |  | | | | |  |
| End of Phase Disposition | 5.1.3 |  | X  Screening Phase  Disposition |  |  |  |  | X  End of Treatment  Phase |  |
| **Physical examination** | **5.1.6** |  | **X** | **X^4^** | **X^4^** | **X** | **X** | **X** |  |
| Performance status (WHO) | 5.1.7 |  | X | X |  |  | X | X |  |
| Height | 5.1.6 |  | X |  |  |  |  |  |  |
| Weight | 5.1.6 |  | X | X |  | X | X | X |  |
| Vital signs | 5.1.6 |  | X | X |  | X | X | X |  |

######

| **Table 5-1.**  **Visit evaluation Schedule**  (continued) | **Protocol**  **Section** | ***EGFR* status** | **Screening / Baseline** | **Day 1 of Cycle 1 (28d) ± 3** | **Day 15 of Cycle 1 (28d) ± 3** | **Day 1 of Cycle 2 (28d) ± 3** | **Day 1 of Subsequent cycles**  **(28d) ± 3^1^** | **End of study treatment (EoT) (at last visit, or up to 30 days from last dose)** | **Long Term Follow Up**  **Q3 mo following EOT** |
| --- | --- | --- | --- | --- | --- | --- | --- | --- | --- |
| **Visit Number** |  |  | **1** | **2** | **3** | **4** | **5, 6,…** | **Last** |  |
| **Day of cycle** |  |  | **-30 to-1** | **1** | **D15** | **D29** | **D57, D85..** | **Last** |  |
| **Lab assessments** |  |  | X | X ^3^ | X | X | X | X |  |
| CBC | 5.1.9 |  | X | X | X | X | X | X |  |
| Chemistry^8^ | 5.1.10 |  | X | X | X | X | X | X |  |
| Creatinine clearance | 5.1.10 |  | X |  | X |  |  |  |  |
| Coagulation (PT/PTT) | 5.1.10 |  | X |  |  |  |  |  |  |
| Urinalysis (dipstick) with microscopic analysis | 5.1.11 |  | X |  | X |  |  | X |  |
| ctDNA blood sample collection | 2.2 |  |  |  |  |  |  | X |  |
| Pregnancy test | 5.1.15 |  | X |  |  | X | X | X |  |
| Standard of care imaging of neck/chest/abdomen (CT, MRI) as indicated to areas of known/ suspected disease^6^ | 5.3.1 |  | X |  |  |  | X |  |  |
| Standard of Care Imaging of the brain as clinically indicated (MRI/CT)^7^ | 5.3.1 |  | X (if known or suspected brain metastases) |  |  |  | X |  |  |
| Adverse events | 7.1 |  | X | Continuous | | | | |  |
| EKG | 5.1.12 |  | X | X |  | X | X | X |  |
| Echocardiogram/MUGA^5^ | 5.1.13 |  | X |  |  |  |  |  |  |
| Drug administration | 4.1 |  |  | Continuous | | | |  |  |
| Survival Assessment | 5.1.17 |  |  |  | | | |  | X |

1. For Cycles 1 through 6 assessments must be performed prior to the start of each treatment cycle. For cycles 7 through 12, assessments will be performed every other cycle. Starting with cycle 13, assessments will be performed every 3 cycles until treatment completion or progression.

2. Patients presenting with signs and symptoms suggestive of keratitis such as acute or worsening: eye inflammation, lacrimation, light sensitivity, blurred vision, eye pain and/or red eye should be referred promptly to an ophthalmology specialist at screening (if symptoms are present) and throughout treatment on study

3. Laboratory assessments must be performed on Cycle 1 Day 1 or within 24 hours prior to dosing.

4. Cycle 1 day 1 Physical exam does not need to be repeated if screening PE was performed within 7 days of C1D1. Physical exam on Day 15 to be performed only if preferred by treating investigator, per standard of care.

5. An echocardiogram or multigated acquisition scan (MUGA) to assess LVEF will be performed at screening (prior to first dose of osimertinib) only in patients with pre-existing cardiac disease that does not meet the criteria for study exclusion but may worsen on treatment with osimertinib. Subsequent assessment of LVEF should be performed as clinically indicated. The modality of the cardiac function assessments must be consistent within a patient, i.e., if echocardiogram is used for the screening assessment then echocardiogram should also be used for subsequent scans. The patients should also be examined using the same machine and operator whenever possible, and quantitative measurements should be taken. A 28-day follow-up assessment will be required if an on-treatment assessment was abnormal at the time of discontinuation of study therapy, to confirm reversibility of the abnormality.

6. CT scans of the neck, chest, abdomen and pelvis as indicated to areas of known/ suspected disease will be performed at baseline and every two cycles for 1 year. Following 1 year of treatment, CT scans will be performed every 3 cycles. Other imaging of these areas such as PET/MRI will be allowed if CT cannot be performed. A window of +/- 7 days is allowed for imaging assessments.

7. MRI of the brain will be performed at baseline if patient has known or suspected brain metastases and every 12 weeks thereafter if patient has known brain metastases.

8. Electrolyte abnormalities (hypokalaemia, hypomagnesaemia, hypocalcaemia) must be corrected to be within normal ranges prior to first dose and electrolyte levels monitored during study treatment.

9. At screening visit, serum pregnancy test will be performed. Following the screening assessment, urine or serum pregnancy tests should be performed.

### Screening/Baseline

Screening assessments to confirm eligibility will be performed as per the schedule of assessments. Documented Informed consent must be obtained before any study specific procedure will be performed.

The patient must have a documented *EGFR mutation* either by sequencing or assay and meet the remainder of the eligibility criteria. All study required testing must be completed within the 30 day period prior to day of initiating therapy.

Re-screening of patients will be allowed, if all entry criteria are met during the re-screening phase time period (-30 days to -1 day).

#### Biomarker required for Eligibility on this trial arm

Patient eligibility will be checked once all screening procedures are completed.

##### ***EGFR* mutation**

Histologically or cytologically confirmed diagnosis of lung adenocarcinoma with *EGFR mutation* will be required to be considered eligible for the therapeutic portion of the trial. *EGFR* testing will be via, standard clinically accepted methods such as:

1. Polymerase chain reaction
2. Gene sequencing
3. NextGeneration sequencing

Documentation of *EGFR mutation* using one of the above tests is required.

#### Patient demographics and other baseline characteristics

Data to be collected on patient characteristics at screening include:

-Demography (including: date of birth, age, patient initials, gender, childbearing potential,

race and ethnicity, or as allowed by local regulations)

-Relevant medical history

-NSCLC diagnosis and extent of disease, including:

Date of diagnosis

Site of active disease

Prior antineoplastic therapies (medications, radiation, surgeries)

Prior and Concomitant Medications, surgical and medical procedures

All other medications taken within 30 days before the first dose of osimertinib treatment is administered will be noted in the clinical trial medication record and updated on a continual basis if there is new change to the medication.

#### Information to be collected on screening failures

A patient who signs an informed consent but fails to satisfy all eligibility criteria for any reason will be considered a screen failure. The reason for not entering the treatment protocol will be recorded. The demographic information, informed consent, and Inclusion/Exclusion pages will be completed for Screen Failure patients. No other data will be entered into the clinical database for patients who are screen failures, unless the patient experienced a Serious Adverse Event during the Screening Phase (see Section 7 for SAE reporting details).

Subjects who signed ICF but are considered ineligible after signing the study consent will be considered as screening failures, and data will be handled in the same manner.

The following information will be recorded for screening failure patients:

-Screening Phase Disposition page (including reason for not satisfying eligibility criteria

and being started on treatment).

-Informed consent.

-Demography.

-Adverse Events (only if an SAE occurs).

-Inclusion/Exclusion Criteria.

### Treatment period

Following completion of screening procedures and verifying patient eligibility, the patient will be approved for treatment per protocol.

The study treatment phase begins on Cycle 1, Day 1 with the first administration of osimertinib and will continue to receive osimertinib treatment until disease progression by RECIST1.1 not amenable to SABR necessitating a change in systemic therapy, unacceptable toxicity, withdrawal of consent, or discontinuation of the trial for any other reason whichever occurs first. Patients who have RECIST-defined PD as assessed by the investigator but who, in the opinion of the investigator, have evidence of continued clinical benefit from osimertinib may continue to receive the study medication upon approval by the principal investigator. In such cases, these patients must continue to be followed for safety and efficacy assessments as per the schedule of assessments. Patients will be assessed as per visit schedule in Table 5-1.

There is a visit window of ± 3 days for scheduled study assessments. A visit delay due to holiday, inclement weather, dosing hold for palliative radiation, or other unforeseen circumstances will be permitted for up to 7 days. The reason for the delay should be documented in the patient’s research record.

As some patients might have symptomatic metastases, they may need palliative radiotherapy during the treatment period:

• Palliative radiotherapy to control symptoms (including gamma knife technique), e.g. to control brain disease, is permitted during osimertinib studies.

• Osimertinib washout before and following brain and thoracic radiotherapy (only to control symptoms) is to be followed per the treating physician’s discretion

• All radiotherapy related toxicities should be managed and ideally resolved before restarting osimertinib.

• Investigators should consider the radiotherapy when assessing causality if there are any localized AEs following the procedure.

### End of treatment visit including study completion and premature withdrawal

#### End of Treatment Disposition

Patients will be evaluated upon discontinuation of the osimertinib by a clinic visit. At that time all assessments listed for End of Treatment will be performed. A note will be entered into the clinical trial record will be completed, giving the date and reason for stopping the osimertinib treatment.

At a minimum, all patients who discontinue osimertinib treatment, including those who refuse to return for a final visit, will be contacted for safety evaluations during the 30 days following the last dose of treatment.

#### Criteria for patient withdrawal

Patients may voluntarily withdraw from the study (no further study data to be collected) at any time.

Patient death will be considered as a withdrawal from the study. Patients may also be withdrawn (the physician may decide to remove the patient from any further study activity) if any of the following occur. Note, data will continue to be collected in a number of the following scenarios; see the applicable sections of the protocol for further information.

- Adverse events (see [Section 7.1](#_441315748461Adverse_events))
- Disease progression
- Major protocol deviation
- Technical problems
- Physician decision
- Non-compliance with study treatment.
- Death
- Completed

Patients must be withdrawn if any of the following occur:

- Lost to follow-up
- Subject/guardian decision
- Pregnancy (Pregnancy will be followed for outcome, but pregnant women will undergo no other study procedures, including any end-of-treatment visits)

Patients lost to follow up should be recorded as such in the clinical trial record. For patients who are lost to follow-up, the investigator will record the attempts at “due diligence” by documenting in the source documents steps taken to contact the patient, e.g. dates of telephone calls, registered letters, etc.

#### Replacement policy

If an eligible patient is unable to start therapy with osimertinib, they may be replaced with another eligible patient if they have not received any study drug.

Apart from the above, patients will not be replaced on this study.

## Assessment types

### Efficacy assessments

Efficacy evaluations will be via revised RECIST 1.1 criteria on imaging performed at the conclusion of every 2 cycles for 1 year. Following 12 cycles on treatment, patients will have imaging evaluations every 3 cycles. Target lesions will be identified prior to initiation of therapy on imaging and will be followed on subsequent imaging.

Physical exam findings of progressive disease will be considered as sufficient for documented progression if the record records biopsy proven tumor measurements in 2 dimensions and shows an increase in both dimensions of 20%. If the progression is amenable to SABR, the patient will receive SABR and be maintained on osimertinib. If the progression is not amenable to SABR and a change in systemic therapy is needed, the patient will be considered to have progressed and have reached the end of the study. New or worsening brain metastasis can be treated with radiotherapy (including gamma knife technique), e.g. while on study if the patient is still benefiting from osimertnib.

Imaging exams will be according to standard of care guidelines to areas of known/suspected disease. These will include CT of the neck, chest, abdomen and pelvis as well as MRI of the brain. Other tests may be clinically indicated, these will be ordered according to disease and patient specific guidelines. Imaging of the brain will be performed at baseline by CT or MRI per standard of care for lung adenocarcinoma.

Subjects with known CNS metastases must have an MRI of the brain performed every 3 cycles while on study. If an MRI cannot be performed, CT of the brain would be an acceptable alternative. Subjects without documented CNS metastases will not have mandated CNS imaging requirements.

### Safety and tolerability assessments

Safety will be monitored by the assessments described below as well as the collection of AEs at every visit. For details on AE collection and reporting, refer to Section 7. Significant findings that were present prior to the signing of informed consent must be included in the relevant medical history/current medical conditions in the clinical trial record. Significant new findings that begin or worsen after informed consent must be recorded in the clinical trial record.

#### Physical examination

A physical examination will be performed and include an assessment of the following: general appearance, skin, head and neck (including ears, eyes, nose and throat), respiratory, cardiovascular, abdomen, lymph nodes, thyroid, musculo-skeletal (including spine and extremities) and neurological systems. Significant findings that were present prior to the signing of informed consent must be included in the clinical trial record. Significant new findings that begin or worsen after informed consent must be recorded in the clinical trial record.

#### Vital signs

Vital signs include body temperature, blood pressure and pulse measurements. Blood pressure (systolic and diastolic) and pulse should be measured.

For the assessment schedule refer to Table 5-1.

#### Height and weight

Height in centimeters (cm) and body weight (to the nearest 0.1 kilogram [kg] in indoor clothing, but without shoes) will be measured. Height will be measured at screening only.

For the assessment schedule for weight refer to Table 5-1.

#### Performance status

WHO performance status will be assessed as per the assessment schedule (refer to Table 5-1).

Assessment of WHO/ECOG performance status (Table 5-2) will be performed within the time windows described above of the scheduled assessment, even if osimertinib medication is being held. More frequent examinations may be performed at the investigator’s discretion, if medically indicated.

###### Table 5-2 WHO/ECOG Performance status scale

| **Score** | **Performance Status** |
| --- | --- |
| 0 | Fully active, able to carry on all pre-disease performance without restriction |
| 1 | Restricted in physically strenuous activity but ambulatory and able to carry out work of a light or  sedentary nature, e.g. light housework, office work |
| 2 | Ambulatory and capable of all self-care but unable to carry out any work activities. Up and about  more than 50% of waking hours |
| 3 | Capable of only limited self-care, confined to bed or chair more than 50% of waking hours |
| 4 | Completely disabled. Cannot carry on any self-care. Totally confined to bed or chair |
| 5 | Dead |

#### Laboratory evaluations

Local site laboratories will be used for the analysis of scheduled hematology, biochemistry, urine, and other blood specimens collected as part of safety monitoring. All unscheduled blood testing will be performed locally, with exceptions for emergency conditions. The time windows granted for laboratory evaluations are identical to the corresponding visit time windows for each visit (refer to Section 5.1).

Laboratory abnormalities that are considered clinically significant, induce clinical signs or symptoms, require concomitant therapy or require changes in osimertinib treatment constitute an adverse event (AE) and must be reported as an AE in the clinical trial record.

Laboratory values obtained at the screening visit will be used to assess eligibility to meet inclusion criteria. In addition, eligible patients must have baseline laboratory assessments performed on Cycle 1 Day 1 or within 24 hours prior to dosing.

##### Hematology

Hematology assessments of the parameters listed in Table 5-3 will be tested as per the schedule of assessments (Table 5-1).

###### Table 5-3 Local Clinical laboratory parameters collection plan

| **Test Category** | **Test Name** |
| --- | --- |
| Hematology | Hgb, platelets, white blood cells (WBC), red blood cells (RBC), differential (basophils, eosinophils, lymphocytes, monocytes, neutrophils (% or absolute) |
| Chemistry | Albumin, ALT, AST, calcium, creatinine, total bilirubin, direct bilirubin (only if total bilirubin is ≥ grade 2), blood urea nitrogen (BUN) or urea, magnesium, potassium, sodium, glucose, phosphate (inorganic phosphorus), alkaline phosphatase, GGT, lipase, amylase |
| Coagulation | PT/PTT |
| Creatinine clearance | Creatinine clearance |
| Urinalysis | Macroscopic panel (dipstick)(color, total bilirubin, blood, glucose, ketones, leukocyte esterase, nitrite, pH, protein, specific gravity, urobilinogen)  Microscopic panel (RBC, WBC, casts, crystals, bacteria, epithelial cells) |
| Pregnancy test | For women of childbearing potential :  At screening visit, serum pregnancy test  At subsequent cycles, urine or serum pregnancy test.  If local requirements dictate otherwise, local regulations should be followed |

##### Clinical chemistry and Creatinine clearance

Clinical chemistry and Creatinine clearance assessments of the parameters listed in Table 5-3 will be tested as per the schedule of assessments (Table 5-1).

##### Urinalysis

Dipstick measurements will be performed as per Table 5-3 and according to the schedule of assessments (Table 5-1). Any significant findings on dipstick will be followed up with microscopic evaluation as per Table 5-3.

##### Pregnancy and assessments of fertility

During screening, a serum pregnancy test will be completed. Starting on day 1 of Cycle 2, and at EOT, urinary pregnancy test (dipstick) or serum test will be performed. The time windows granted for pregnancy testing are identical to the corresponding visit time windows for each visit. If local requirements dictate otherwise, local regulations should be followed.

Pregnancy tests are not required for women who are determined not to be of child bearing potential before the study. Women are considered post-menopausal if they have had 12 months of natural (spontaneous) amenorrhea with an appropriate clinical profile (e.g. age appropriate, history of vasomotor symptoms), and otherwise not of child bearing potential if they have had surgical bilateral oophorectomy (with or without hysterectomy) or tubal ligation at least six weeks ago. In the case of oophorectomy alone, only when the reproductive status of the woman has been confirmed by follow up hormone level assessment is she considered not of child bearing potential.

If a positive pregnancy test is performed in between study visits, the patients must immediately notify the investigator.

#### Cardiac assessments

##### Electrocardiogram (ECG)

A twelve-lead ECG will be performed at the visit indicated in the Study Plan.

If there is a clinically significant abnormal ECG finding during the Treatment period, this should be recorded on the AE eCRF, according to standard adverse events collection and reporting processes. A 28-day follow-up assessment will be required if an on-treatment assessment was abnormal at the time of discontinuation of study therapy, to confirm reversibility of the abnormality.

##### Echocardiogram or MUGA: An echocardiogram or multigated acquisition scan (MUGA) to assess LVEF will be performed at screening (prior to first dose of osimertinib) only in patients with pre-existing cardiac disease that does not meet the criteria for study exclusion but may worsen on treatment with osimertinib. Subsequent assessment of LVEF should be performed as clinically indicated. The modality of the cardiac function assessments must be consistent within a patient, i.e., if echocardiogram is used for the screening assessment then echocardiogram should also be used for subsequent scans. The patients should also be examined using the same machine and operator whenever possible, and quantitative measurements should be taken. A 28-day follow-up assessment will be required if an on-treatment assessment was abnormal at the time of discontinuation of study therapy, to confirm reversibility of the abnormalityMeasurement of Effect

# Measurement of Effect

## Antitumor Effect – Solid Tumors

Response and progression will be evaluated in this study using the new international criteria proposed by the Response Evaluation Criteria in Solid Tumors (RECIST v 1.1) Committee [Eur J Cancer. 2009;45(2):228-247]. Changes in only the largest diameter (unidimensional measurement) of the tumor lesions are used in the RECIST v1.1 criteria.

### Definitions

Evaluable for toxicity. All subjects will be evaluable for toxicity from the time of their first treatment with study therapy.

Evaluable for objective response. Only those subjects who have measurable disease present at baseline, have received at least one cycle of therapy, and have had their disease re-evaluated will be considered evaluable for response. These subjects will have their response classified according to the definitions stated below. (Note: Subjects who exhibit objective disease progression or die prior to the end of cycle 1 will also be considered evaluable.)

### Disease Parameters

Measurable disease: Tumor lesions: Must be accurately measured in at least one dimension (longest diameter in the plane of measurement is to be recorded) with a minimum size of:

1. 10 mm by CT scan (CT scan slice thickness no greater than 5 mm)

2. 10 mm caliper measurement by clinical exam (lesions which cannot be accurately measured with calipers should be recorded as non-measurable)

3. 20 mm by chest x-ray.

Malignant lymph nodes: To be considered pathologically enlarged and measurable, a lymph node must be ≥ 15 mm in short axis when assessed by CT scan (CT scan slice thickness recommended to be no greater than 5 mm). Lymph nodes merit special mention since they are normal anatomical structures which may be visible by imaging even if not involved by tumor. Pathological nodes which are defined as measurable and may be identified as target lesions must meet the criterion of a short axis of ≥ 15 mm by CT scan. Only the short axis of these nodes will contribute to the baseline sum. The short axis of the node is the diameter normally used by radiologists to judge if a node is involved by solid tumor. Nodal size is normally reported as two dimensions in the plane in which the image is obtained (for CT scan this is almost always the axial plane; for MRI the plane of acquisition may be axial, sagittal or coronal). The smaller of these measures is the short axis. For example, an abdominal node which is reported as being 20 mm x 30 mm has a short axis of 20 mm and qualifies as a malignant, measurable node. In this example, 20 mm should be recorded as the node measurement. All other pathological nodes (those with short axis ≥ 10 mm but < 15 mm) should be considered non-target lesions. Nodes that have a short axis < 10 mm are considered non-pathological and should not be recorded or followed.

Note: Previously irradiated lesions are non-measurable except in cases of documented progression of the lesion since the completion of radiation therapy.

Non-measurable disease.

All other lesions are considered non-measurable, including small lesions (longest diameter < 10mm or pathological lymph nodes with ≥ 10 to < 15 mm short axis) as well as truly non-measurable lesions. Lesions considered truly non-measurable include: leptomeningeal disease, ascites, pleural or pericardial effusion, inflammatory breast disease, lymphangitic involvement of skin or lung, abdominal masses/abdominal organomegaly identified by physical exam that is not measurable by reproducible imaging techniques.

Target lesions. All measurable lesions up to a maximum of five lesions total (and a maximum of two lesions per organ), representative of all involved organs should be identified as target lesions**.**

Non-target lesions. All other lesions (or sites of disease) including any measurable lesions over and above the five target lesions should be identified as **non-target lesions** and should also be recorded at baseline. Measurements of these lesions are not required, but the presence or absence of each should be noted throughout follow-up.

### Methods for Evaluation of Measurable Disease

All measurements should be taken and recorded in metric notation using a ruler or calipers. All baseline evaluations should be performed as closely as possible to the beginning of treatment and not more than 30 days before the beginning of the treatment.

The same method of assessment and the same technique should be used to characterize each identified and reported lesion at baseline and during follow-up. Imaging-based evaluation is preferred to evaluation by clinical examination when both methods have been used to assess the antitumor effect of a treatment.

Conventional CT and MRI. These techniques should be performed with cuts of 10 mm or less in slice thickness contiguously. Spiral CT should be performed using a 5 mm contiguous reconstruction algorithm. This applies to tumors of the chest, abdomen, and pelvis.

CT scans of the neck, chest, abdomen and pelvis will be performed at baseline and every two cycles (or 8 weeks) according to standard of care for 1 year. Following 1 year of treatment, CT scans will be performed every 3 cycles Other imaging of these areas such as PET/MRI will be allowed if CT cannot be performed.

MRI of the brain will be performed at baseline if patient has known or suspected brain metastases and every 12 weeks thereafter if patient has known brain metastases. Wherever it can be safely given, radiographic contrast agents should be given for the imaging studies.

### Response Criteria

#### Evaluation of Target Lesions

Complete Response (CR): Disappearance of all target lesions.

Any pathological lymph nodes (whether target or non-target) must have reduction in short axis to <10 mm (the sum may not be “0” if there are target nodes). Determined by two separate observations conducted not less than 4 weeks apart. There can be no appearance of new lesions.

Partial Response (PR): At least a 30% decrease in the sum of the longest diameter (SLD) of target lesions, taking as reference the baseline SLD. There can be no appearance of new lesions.

Progressive Disease (PD): > 20% increase in the SLD taking as reference the smallest SLD recorded since the treatment started (nadir) and minimum 5 mm increase over the nadir.

Stable Disease (SD): Neither sufficient shrinkage to qualify for PR nor sufficient increase to qualify for PD, taking as reference the smallest SLD since the treatment started. There can be no unequivocal new lesions.

While on study, should a chosen Target lesion become non-evaluable, document as Not Evaluable (NE).

#### Evaluation of Non-Target Lesions

Complete Response (CR): Disappearance of all non-target lesions and normalization of tumor marker level. All lymph nodes must be non-pathological in size (< 10 mm short axis).

Non-CR/Non-PD): Persistence of one or more non-target lesion(s) and/or maintenance of tumor marker level above the normal limits.

Progressive Disease (PD): Appearance of one or more new lesions and/or unequivocal progression of existing non-target lesions

While on study, should a chosen Non-Target lesion become non-evaluable, document as Not Evaluable (NE).

#### Evaluation of Best Overall Response

The best overall response is the best response recorded from the start of the treatment until disease progression/recurrence (taking as reference for progressive disease the smallest measurements recorded since the treatment started). The subject’s best response assignment will depend on the achievement of both measurement and confirmation criteria.

| Time point response: patients with target (+/– non-target) disease. | | | |
| --- | --- | --- | --- |
| **Target lesions** | **Non-target lesions** | **New lesions** | **Overall response** |
| CR | CR | No | CR |
| CR | Non-CR/non-PD | No | PR |
| CR | Not evaluated | No | PR |
| PR | CR | No | PR |
| PR | Non-CR/non-PD | No | PR |
| PR | Not all evaluated | No | PR |
| Not all evaluated | CR | No | NE |
| Not all evaluated | Non-CR/non-PD | No | NE |
| SD | Non-PD or not all evaluated | No | SD |
| Not all evaluated | CR | No | NE |
| Not all evaluated | Non-CR/non-PD | No | NE |
| Or Not all evaluated | Non-PD | No | NE |
| PD | Any | Yes or No | PD |
| Any | PD | Yes or No | PD |
| Any | Any | Yes | PD |

CR = complete response, NE = not evaluable, PD = progressive disease, PR = partial response, SD = stable disease.

| Time point response: patients with non-target disease only. | | |
| --- | --- | --- |
| Non-target lesions | New lesions | Overall response |
| CR | No | CR |
| Non-CR/non-PD | No | Non-CR/non-PD |
| Not all evaluated | No | NE |
| Unequivocal PD | Yes or No | PD |
| Any | Yes | PD |
| CR = complete response, NE = not evaluable, PD = progressive disease | | |
|  | | |

### Duration of Response

Duration of overall response: The duration of overall response is measured from the time measurement criteria are met for CR or PR (whichever is first recorded) until the first date that recurrent or progressive disease is objectively documented (taking as reference for progressive disease the smallest measurements recorded since the treatment started).

The duration of overall CR: The duration of overall CR is measured from the time measurement criteria are first met for CR until the first date that recurrent disease is objectively documented.

### Duration of stable disease: Stable disease is measured from the start of the treatment until the criteria for progression are met, taking as reference the smallest measurements recorded since the treatment started. Overall Survival

Overall Survival is defined as the time from the date of initiation of osimertinib until death of any cause.

### Progression-Free Survival

Progression-free survival is defined as the time from first dose of osimertinib to date of objective progression (as determined by the investigator according to RECIST 1.1) or death (in the absence of progression). Patients will still be followed for progression even if they stop due to toxicity or start a subsequent therapy prior to progression.

### Time to Subsequent SABR

Time to 2nd SABR is defined as the duration of time from the start of treatment to the time of development of new or enlarging lesions that are amenable to SABR and the progression does not require a discontinuation of osimertinib.

Time to 3^rd^ SABR is defined in patients who have already received two rounds of SABR while on this study as the duration of time from the start of treatment to the time of development of new or enlarging lesions that are amenable to SABR and the progression does not require a discontinuation of osimertinib.

After the initial SABR to persisting lesions if new lesions appear or if existing lesions enlarge but are NOT amenable to SABR (necessitating a change in systemic therapy), the patient will have completed the therapeutic portion of the study.

### Time until discontinuation of osimertinib or Death

This is defined as the time from first dose to the earlier of the date of treatment discontinuation or death. Any patient not known to have died at the time of analysis and not known to have discontinued study treatment will be censored based on the last recorded date on which the patient was known to be alive.

# Adverse events

## Definition of adverse events

An adverse event is the development of an undesirable medical condition or the deterioration of a pre-existing medical condition following or during exposure to a pharmaceutical product, whether or not considered causally related to the product. An undesirable medical condition can be symptoms (e.g., nausea, chest pain), signs (e.g., tachycardia, enlarged liver) or the abnormal results of an investigation (e.g., laboratory findings, electrocardiogram). In clinical studies, an AE can include an undesirable medical condition occurring at any time, including run-in or washout periods, even if no study treatment has been administered. The term AE is used to include both serious and non-serious AEs.

## Definitions of serious adverse event

A serious adverse event is an AE occurring during any study phase (i.e., run-in, treatment, washout, follow-up), that fulfils one or more of the following criteria:

- Results in death
- Is immediately life-threatening
- Requires in-patient hospitalisation or prolongation of existing hospitalisation
- Results in persistent or significant disability/incapacity or substantial disruption of the ability to conduct normal life functions
- Is a congenital abnormality or birth defect
- Is an important medical event that may jeopardise the subject or may require medical intervention to prevent one of the outcomes listed above.
- Based upon appropriate medical judgment, may jeopardize the patient’s health and may require medical or surgical intervention to prevent one of the other outcomes listed in this definition.

The causality of SAEs (their relationship to all study treatment/procedures) will be assessed by the investigator(s) and communicated to AstraZeneca in accordance with the agreed process.

It is important to distinguish between serious and severe AEs. Severity is a measure of intensity whereas seriousness is defined by the criteria in Section 7.2. An AE of severe intensity need not necessarily be considered serious. For example, nausea that persists for several hours may be considered severe nausea, but not a SAE unless it meets the criteria shown in Section 7.2. On the other hand, a stroke that results in only a limited degree of disability may be considered a mild stroke but would be a SAE when it satisfies the criteria shown in Section 7.2.

## Contraindications:

Osimertinib is contraindicated in individuals who are hypersensitive to any component of the finished dosage forms. No other contraindications are listed in the package insert.

## Special Warnings and Precautions for Use:

### Interstitial Lung Disease/Pneumonitis:

Across clinical trials, interstitial lung disease (ILD)/pneumonitis occurred in 3.3% (n=27) of osimertinib treated patients (n=813); 0.5% (n=4) were fatal. Withhold osimertinib and promptly investigate for ILD in any patient who presents with worsening of respiratory symptoms which may be indicative of ILD (e.g., dyspnea, cough and fever). Permanently discontinue osimertinib if ILD is confirmed

### QTc Interval Prolongation:

The heart rate-corrected QT (QTc) interval prolongation occurs in patients treated with osimertinib. Of the 411 patients in Study 1 and Study 2, one patient (0.2%) was found to have a QTc greater than 500 msec, and 11 patients (2.7%) had an increase from baseline QTc greater than 60 msec [see Clinical Pharmacology (12.2)]. In Study 1 and 2, patients with baseline QTc of 470 msec or greater were excluded. Conduct periodic monitoring with ECGs and electrolytes in patients with congenital long QTc syndrome, congestive heart failure, electrolyte abnormalities, or those who are taking medications known to prolong the QTc interval. Permanently discontinue osimertinib in patients who develop QTc interval prolongation with signs/symptoms of life threatening arrhythmia

### Cardiomyopathy

Across clinical trials, cardiomyopathy (defined as cardiac failure, pulmonary edema, ejection fraction

decreased or stress cardiomyopathy) occurred in 1.4% (n=11) of osimertinib treated patients (n=813);

0.2% (n=2) were fatal. In Study 1 and Study 2, Left Ventricular Ejection Fraction (LVEF) decline >10% and a drop to <50% occurred in 2.4% (9/375) of patients who had baseline and at least one follow up LVEF assessment. Assess LVEF by echocardiogram before initiation of osimertinib and then at 3 month intervals while on treatment. Withhold treatment with osimertinib if ejection fraction decreases by 10% from pretreatment values and is less than 50%. For symptomatic congestive heart failure or persistent, asymptomatic LV dysfunction that does not resolve within 4 weeks, permanently discontinue osimertinib

### Embryo-Fetal Toxicity:

Based on data from animal studies and its mechanism of action, osimertinib can cause fetal harm when administered to a pregnant woman. In animal reproduction studies, osimertinib caused post-implantation fetal loss when administered during early development at a dose exposure 1.5 times the exposure at the recommended human dose. When males were treated prior to mating with untreated females, there was an increase in preimplantation embryonic loss at plasma exposures of approximately 0.5-times those observed in patients at the 80 mg dose level. Advise pregnant women of the potential risk to a fetus. Advise females of reproductive potential to use effective contraception during treatment with osimertinib and for 6 weeks after the final dose. Advise males with female partners of reproductive potential to use effective contraception for 4 months after the final dose.

## Adverse Reactions

Because clinical trials are conducted under widely varying conditions, adverse reaction rates observed in the clinical trials of a drug cannot be directly compared to rates in the clinical trials of another drug and may not reflect the rates observed in practice.

The data described below reflect exposure to osimertinib (80 mg daily) in 411 patients with EGFR T790M mutation-positive non-small cell lung cancer who received prior EGFR TKI therapy, in two single arm studies, AURA 1 and AURA 2 Patients with a past medical history of ILD or radiation pneumonitis that required steroid treatment, serious arrhythmia or baseline QTc interval greater than 470 ms were excluded from AURA 1 and AURA 2. Baseline patient and disease characteristics were: median age 63 years, 13% of patients were ≥75 years old, female (68%); White (36%), Asian (60%);metastatic disease (96%), history of brain metastases (39%); World Health Organization (WHO) performance status of 0 (37%) or 1 (63%); 1 prior line of therapy [EGFR-TKI treatment only, second line, chemotherapy-naïve (31%)], 2 or more prior lines of therapy (69%). Of the 411 patients, 333 patients were exposed to osimertinib for at least 6 months; 97 patients were exposed for at least 9 months; however, no patient was exposed to osimertinib for 12 months.

In AURA1 and AURA2 studies, the most common (>20%) adverse reactions (all grades) observed in osimertinib- treated patients were diarrhea (42%), rash (41%), dry skin (31%), and nail toxicity (25%). Dose reductions occurred in 4.4% of patients treated with osimertinib. The most frequent adverse reactions that led to dose reductions or interruptions were: electrocardiogram QTc prolonged (2.2%) and neutropenia (1.9%). Serious adverse reactions reported in 2% or more patients were pneumonia and pulmonary embolus. There were 4 patients (1%) treated with osimertinib who developed fatal adverse reactions of ILD/pneumonitis. Other fatal adverse reactions occurring in more than 1 patient included pneumonia (4 patients) and CVA/cerebral hemorrhage (2 patients). Discontinuation of therapy due to adverse reactions occurred in 5.6% of patients treated with osimertinib. The most frequent adverse reactions that led to discontinuation were ILD/pneumonitis and cerebrovascular accidents/infarctions.

Tables 2 and 3 summarize the common adverse reactions and laboratory abnormalities observed in osimertinib-treated patients.

## Table 7.6.1, Adverse Reactions (>10% for all NCI CTCAE* Grades or >2% for Grades 3-4) in AURA1 and AURA2

| **Adverse Reaction** | **Osimertinib N=411** | |
| --- | --- | --- |
|  | **All Grades** | **Grade 3-4** |
|  | **%** | **%** |
| **Gastrointestinal disorders** |  |  |
| Diarrhea | 42 | 1.0 |
| Nausea | 17 | 0.5 |
| Decreased appetite | 16 | 0.7 |
| Constipation | 15 | 0.2 |
| Stomatitis | 12 | 0 |
| **Skin disorders** |  |  |
| Rasha | 41 | 0.5 |
| Dry skinb | 31 | 0 |
| Nail toxicityc | 25 | 0 |
| Pruritus | 14 | 0 |
| **Eye Disorders**d | 18 | 0.2 |
| **Respiratory** |  |  |
| Cough | 14 | 0.2 |
| **General** |  |  |
| Fatigue | 14 | 0.5 |
| **Musculoskeletal** |  |  |
| Back pain | 13 | 0.7 |
| **Central Nervous System** |  |  |
| Headache | 10 | 0.2 |
| **Infections** |  |  |
| Pneumonia | 4 | 2.2 |
| **Vascular events** |  |  |
| Venous thromboembolisme | 7 | 2.4 |

* NCI CTCAE v4.0.

aIncludes cases reported within the clustered terms for rash adverse events: Rash, rash generalized, rash erythematous, rash macular, rash maculo-papular, rash papular, rash pustular, erythema, folliculitis, acne, dermatitis and acneform dermatitis.

bIncludes dry skin, eczema, skin fissures, xerosis.

cIncludes nail disorders, nail bed disorders, nail bed inflammation, nail bed tenderness, nail discoloration, nail disorder, nail dystrophy, nail infection, nail ridging, onychoclasis, onycholysis, onychomadesis, paronychia.

dIncludes dry eye, vision blurred, keratitis, cataract, eye irritation, blepharitis, eye pain, lacrimation increased, vitreous floaters. Other ocular toxicities occurred in <1% of patients.

eIncludes deep vein thrombosis, jugular venous thrombosis, and pulmonary embolism.

fNo grade 4 events have been reported.

Additional clinically significant adverse reactions occurring in 2% or more of patients treated with osimertinib included cerebrovascular accident (2.7%).

## Table 7.6.2, Common Laboratory Abnormalities (>20% for all NCI CTCAE Grades) in AURA1 and AURA2.

| **Laboratory Abnormality** | **Osimertinib N=411** | |
| --- | --- | --- |
|  | **Change from Baseline All Grades (%)** | **Change from Baseline to Grade 3 or Grade 4** |
| **Clinical Chemistry** |  |  |
| Hyponatremia | 26 | 3.4 |
| Hypermagnesemia | 20 | 0.7 |
| **Hematologic** |  |  |
| Lymphopenia | 63 | 3.3 |
| Thrombocytopenia | 54 | 1.2a |
| Anemia | 44 | 0.2 |
| Neutropenia | 33 | 3.4 |

aThe only grade 4 laboratory abnormality was 1 patient with grade 4 thrombocytopenia.

All Adverse Drug Reactions (ADRs) are considered unexpected if the outcome is fatal, except ILD/pneumonitis, which is considered expected for all severity grades, including ILD/pneumonitis with fatal outcome.

## Adverse Event Monitoring

Adverse event data collection and reporting, which are required as part of every clinical trial, are done to ensure the safety of subjects enrolled in the studies as well as those who will enroll in future studies using similar agents. Adverse events are assessed in a routine manner at scheduled times during a trial. Additionally, certain adverse events must be reported in an expedited manner to allow for optimal monitoring of subject safety and care.

An adverse event is defined as any untoward or unfavorable medical occurrence in a human research study participant, including any abnormal sign (for example, abnormal physical exam or laboratory finding), symptom, clinical event, or disease, temporarily associated with the patient’s participation in the research, whether or not it is considered related to the patient’s participation in the research.

Adverse events encompass clinical, physical and psychological harms. Adverse events occur most commonly in the context of biomedical research, although on occasion, they can occur in the context of social and behavioral research. Adverse events may be expected or unexpected.

All subjects experiencing an adverse event, regardless of its relationship to study therapy, will be monitored until:

- the adverse event resolves or the symptoms or signs that constitute the adverse event return to baseline;
- any abnormal laboratory values have returned to baseline;
- there is a satisfactory explanation other than the study therapy for the changes observed; or
- death

Note: Adverse events that occur from consent to within 30 days of the last dose of protocol treatment (alternatively, to the end of the acute adverse events reporting period as defined in section 7.5 will be documented . Any event that occurs more than 30 days after the last dose of treatment (alternatively, during the late adverse event period as defined in section 7.5 and is attributed (possibly, probably, or definitely) to the agent(s) must also be documented and reported if it meets Serious Adverse Event criteria (See Section 7.3).

Severity

Adverse events will be graded by a numerical score according to the defined NCI Common Terminology Criteria for Adverse Events (NCI CTCAE) and version number specified in the protocol. Adverse events not specifically defined in the NCI CTCAE will be scored on the Adverse Event log according to the general guidelines provided by the NCI CTCAE and as outlined below.

• Grade 1: Mild

• Grade 2: Moderate

• Grade 3: Severe or medically significant but not immediately life threatening

• Grade 4: Life threatening consequences

• Grade 5: Death related to the adverse event

Serious Adverse Events

ICH Guideline E2A and the UTSW IRB define serious adverse events as those events, occurring at any dose, which meets any of the following criteria:

• Results in death

• Immediately life-threatening

• Results in inpatient hospitalization or prolongation of existing hospitalization

• Results in persistent or significant disability/incapacity

• Results in a congenital anomaly/birth defect

• Based upon appropriate medical judgment, may jeopardize the patient’s health and may require medical or surgical intervention to prevent one of the other outcomes listed in this definition.

Note: A “Serious adverse event” is by definition an event that meets any of the above criteria. Serious adverse events may or may not be related to the research project. A serious adverse event determination does not require the event to be related to the research. That is, both events completely unrelated to the condition under study and events that are expected in the context of the condition under study may be serious adverse events, independent of relatedness to the study itself. As examples, a car accident requiring overnight hospitalization would be a serious adverse event for any research participant; likewise, in a study investigating end-stage cancer care, any hospitalization or death which occurs during the protocol-specified period of monitoring for adverse and serious adverse events would be a serious adverse event, even if the event observed is a primary clinical endpoint of the study

.

Adverse Events (AEs) for new malignant tumors (i.e., it is not the tumor for which entry into the study is a criterion and that is being treated by the IP under study and is not the development of new or progression of existing metastasis to the tumor under study) reported during a study should generally be assessed as Serious AEs. If no other seriousness criteria apply, the ‘Important Medical Event’ criterion should be used. In certain situations, however, medical judgement on an individual event basis should be applied to clarify that the malignant tumor event should be assessed and reported as a Non-Serious AE. For example, if the tumor is included as medical history, and progression occurs during the study, but the progression does not change treatment and/or prognosis of the malignant tumor, the AE may not fulfill the attributes for being assessed as Serious, although reporting of the progression of the malignant tumor as an AE is valid and should occur. Also, some types of malignant tumors, which do not spread remotely after a routine treatment that does not require hospitalization, may be assessed as Non-Serious; examples include Stage 1 basal cell carcinoma and Stage 1A1 cervical cancer removed via cone biopsy

### Unanticipated Problems Involving Risks to Subjects or Others (UPIRSOs)

The term “unanticipated problem” is found, but not defined in the regulations for the Protection of Human Subjects at 45 CFR 46, and the FDA regulations at 21 CFR 56. Guidance from the regulatory agencies considers unanticipated problems to include any incident, experience, or outcome that meets ***each*** of the following criteria:

- - Unexpected (in terms of nature, severity or frequency) ***AND***
  - Definitely or probably related to participation in the research ***AND***
  - Serious or a possible unexpected problem in that the research places subjects or others at greater risk of harm than was previously known or recognized. Note: Any serious adverse event would always suggest a greater risk of harm.

Follow-up

All adverse events will be followed up according to good medical practices.

### Disease progression

Disease progression can be considered as a worsening of a subject’s condition attributable to the disease for which the investigational product is being studied. It may be an increase in the severity of the disease under study and/or increases in the symptoms of the disease. The development of new, or progression of existing metastasis to the primary cancer under study should be considered as disease progression and not an AE. Events, which are unequivocally due to disease progression, should not be reported as an AE during the study.

### New cancers

The development of a new primary cancer should be regarded as an AE and will generally meet at least one of the serious criteria. New primary cancers are those that are not the primary reason for the administration of the study treatment and have developed after the inclusion of the patient into the study. They do not include metastases of the original cancer. Symptoms of metastasis or the metastasis itself should not be reported as an AE/SAE, as they are considered to be disease progression.

### Lack of efficacy

When there is deterioration in the condition for which the study treatment(s) is being used, there may be uncertainty as to whether this is lack of efficacy or an AE. In such cases, unless the Sponsor or the reporting physician considers that the study treatment contributed to the deterioration of the condition, or local regulations state to the contrary, the deterioration should be considered to be a lack of efficacy and not an AE.

### Deaths

All deaths that occur during the study, or within the protocol‑defined 30‑day post‑study follow‑up period after the administration of the last dose of study treatment, must be reported as follows:

- Death clearly the result of disease progression should be reported to the study monitor at the next monitoring visit and should be documented in the eCRF but should not be reported as an SAE.
- Where death is not due (or not clearly due) to progression of the disease under study, the AE causing the death must be reported to the UTSW study team as an SAE within **24 hours** The report should contain a comment regarding the co‑involvement of progression of disease, if appropriate, and should assign main and contributory causes of death. This information will also be captured under cause of death in the ‘Survival Follow-up eCRF’.
- Deaths with an unknown cause should always be reported as a SAE. A post mortem maybe helpful in the assessment of the cause of death, and if performed a copy of the post-mortem results should be forwarded to AstraZeneca within the usual timeframes.

## Reporting adverse events

The UTSW IRB requires reporting of all UPIRSOs according to the guidance below. For participating centers other than UTSW, local IRB guidance should be followed for local reporting of serious adverse events. All SAEs occurring during the protocol-specified monitoring period should be submitted to the UTSW study team within 2 business days of the center learning of the event.

UPIRSOs occurring on the study require expedited reporting, and are submitted to the UTSW IRB through the UTSW eIRB by the UTSW study team and to the SCCC DSMC Coordinator. Hardcopies or electronic versions of the eIRB report; FDA Form #3500A forms, or other sponsor forms, if applicable; and/or any other supporting documentation available should be submitted to the UTSW study team and will be forwarded to the DSMC Coordinator. The DSMC Coordinator forwards the information onto the DSMC Chairman who determines if immediate action is required. Follow-up eIRB reports, and all subsequent documentation that is available are also submitted to the DSMC Chair who determines if further action is required.

All serious adverse events which occur on research patients on protocols for which the SCCC is the DSMC of record require reporting to the DSMC regardless of whether IRB reporting is required. Hardcopies or electronic versions of the FDA Form #3500A forms, or other sponsor forms, if applicable; and/or any other supporting documentation available should be forwarded to the DSMC Coordinator.

If the event occurs on a multi-institutional clinical trial coordinated by the UTSW Simmons Cancer Center, the DOT Manager or lead coordinator ensures that all participating sites are notified of the event and resulting action, according to FDA guidance for expedited reporting. DSMC Chairperson reviews all serious adverse events upon receipt from the DSMC Coordinator. The DSMC Chairperson determines whether action is required and either takes action immediately, convenes a special DSMC session (physical or electronic), or defers the action until a regularly scheduled DSMC meeting.

| Written reports to:  Sawsan Rashdan, MD  Sawsan.Rashdan@utsouthwestern.edu  c/o Lung DOT Manager  5323 Harry Hines Blvd, NB2.402  Fax: 214-648-1578  UTSW SCCC Data Safety Monitoring Committee Coordinator  Email: SCCDSMC@utsouthwestern.edu  Fax: 214-648-5949 or deliver to BLB.306  UTSW Institutional Review Board (IRB)  Submit via eIRB with a copy of the final sponsor report as attached supporting documentation |
| --- |

SAEs

Serious adverse events (SAEs) for studies where the SCCC DSMC is the DSMC of record require reporting to the DSMC coordinator within 2 working days of PI awareness, or as described in the protocol.

Adverse event reporting to AstraZeneca.

The investigator/Lead coordinator shall inform the FDA, via a MedWatch/AdEERs form, of any serious or unexpected adverse events that occur in accordance with the reporting obligations of 21 CFR 312.32, and will concurrently forward all such reports to AstraZeneca. A copy of the MedWatch/AdEERs report must be faxed or **email** to AstraZeneca at the time the event is reported to the FDA. It is the responsibility of the investigator to compile all necessary information and ensure that the FDA receives a report according to the FDA reporting requirement timelines and to ensure that these reports are also submitted to AstraZeneca at the same time.

***** A ***cover page*** should accompany the ***MedWatch/AdEERs*** form indicating the following:

- Investigator Sponsored Study (ISS)
- The investigator IND number assigned by the FDA
- The investigator’s name and address
- The trial name/title and AstraZeneca ISS reference number

******* Investigative site must also indicate, either in the SAE report or the cover page, the ***causality*** of events ***in relation to all study medications*** and if the SAE is ***related to disease progression***, as determined by the principal investigator.

SAE report and accompanying cover page to AstraZeneca will be sent by email to AE Mailbox Clinical Trial (TCS) <[AEMailboxClinicalTrialTCS@astrazeneca.com](mailto:AEMailboxClinicalTrialTCS@astrazeneca.com)> or by fax to 1-302-886-4114 (US Fax number). Email is the preferred method.

If a non-serious AE becomes serious, this and other relevant follow-up information must also be provided to AstraZeneca and the FDA.

Serious adverse events that do not require expedited reporting to the FDA need to be reported to AstraZeneca preferably using the MedDRA coding language for serious adverse events. This information should be reported on a monthly basis and under no circumstance less frequently than quarterly.

For all studies of osimertinib, the Sponsor should continue sending SUSARs to AstraZeneca in parallel to regulatory authorities as defined per AZ standard minimum requirements for safety data collection; all other SAEs should be sent to AZ on a monthly basis.

All SAEs have to be reported to AstraZeneca, whether or not considered causally related to the investigational product. All SAEs will be documented. The investigator is responsible for informing the IRB and/or the Regulatory Authority of the SAE as per local requirements.

Non-serious adverse events and SAEs will be collected from the time consent is given, throughout the treatment period and up to and including the *30 day follow-up* period. After withdrawal from treatment, subjects must be followed-up for all existing and new AEs for *30 calendar days after the last dose of trial drug and/or until event resolution.*  All new AEs occurring during that period must be recorded (if SAEs, then they must be reported to the FDA and AstraZeneca). All study-related toxicities/ SAEs must be followed until resolution, unless in the Investigator’s opinion, the condition is unlikely to resolve due to the patient’s underlying disease.

Recording of adverse events:

Variables:

- AE (verbatim)
- The date <<and time*>>* when the AE started and stopped
- Whether the AE is serious or not
- Investigator causality rating against the Investigational Product (yes or no), comparator/combination drug (yes/no)
- Action taken with regard to investigational product/ comparator/combination agent
- AE caused subject’s withdrawal from study *(*yes or no*)*
- Outcome.

In addition, the following variables will be collected for SAEs:

- Date AE met criteria for serious AE
- Date Investigator became aware of serious AE
- AE is serious due to
- Date of hospitalisation
- Date of discharge
- Probable cause of death
- Date of death
- Autopsy performed
- Causality assessment in relation to Study procedure(s)
- Causality assessment in relation to Other medication
- <<Causality assessment in relation to Additional Study Drug>>
- Description of AE.

### Unanticipated Problems Involving Risks to Subjects or Others (UPIRSOs)

Local Serious Adverse Event UPIRSOs require reporting to the UTSW IRB within 48 hours of PI awareness of the event (life threatening or fatal events experienced by patients enrolled by the investigator(s) under UTSW IRB jurisdiction).

Local UPIRSOs (non-serious events experienced by patients enrolled by the investigator(s) under UTSW IRB jurisdiction) require reporting to the UTSW IRB within 5 business days of PI awareness.

External UPIRSOs including those that occur as non-local events, require reporting to the UTSW IRB within 10 working days of PI awareness of the event.

For further guidance for Investigators regarding safety reporting requirements for INDs and BA/BE studies, refer to FDA Draft Guidance document:http://www.fda.gov/downloads/Drugs/GuidanceComplianceRegulatoryInformation/Guidances/UCM227351

### Steps to Determine If an Adverse Event Requires Expedited Reporting

Step 1: Identify the type of adverse event using the NCI Common Terminology Criteria for Adverse Events (CTCAE v4).

Step 2: Grade the adverse event using the NCI CTCAE v4.

Step 3: Determine whether the adverse event is related to the protocol therapy

Attribution categories are as follows:

- - Definite – The AE *is clearly related* to the study treatment.
  - Probable – The AE *is likely related* to the study treatment.
  - Possible – The AE *may be related* to the study treatment.
  - Unrelated – The AE *is clearly NOT related* to the study treatment.

Note: This includes all events that occur within 30 days of the last dose of protocol treatment. Any event that occurs more than 30 days after the last dose of treatment and is attributed (possibly, probably, or definitely) to the agent(s) must also be reported accordingly.

Step 4: Determine the prior experience of the adverse event.

Expected events are those that have been previously identified as resulting from administration of the agent. An adverse event is considered unexpected, for expedited reporting purposes only, when either the type of event or the severity of the event is not listed in:

- the current known adverse events listed in the Agent Information Section of this protocol
- the drug package insert;
- the current Investigator’s Brochure

### Adverse events based on signs and symptoms

All AEs spontaneously reported by the care provider or revealed by observation will be collected and recorded in the CRF. When collecting AEs, the recording of diagnoses is preferred (when possible) to recording a list of signs and symptoms. However, if a diagnosis is known and there are other signs or symptoms that are not generally part of the diagnosis, the diagnosis and each sign or symptom will be recorded separately.

### Adverse events based on examinations and tests

Deterioration as compared to baseline in protocol-mandated laboratory values, vital signs, physical examination, performance status and imaging should only be reported as AEs if they fulfill any of the SAE criteria or are the reason for discontinuation of treatment with the investigational product.

If deterioration in a laboratory value/vital sign is associated with clinical signs and symptoms, the sign or symptom will be reported as an AE and the associated laboratory result/vital sign will be considered as additional information. Wherever possible the reporting Investigator uses the clinical, rather than the laboratory term (e.g., anaemia versus low haemoglobin value). In the absence of clinical signs or symptoms, clinically relevant deteriorations in non-mandated parameters should be reported as AE(s). Deterioration of a laboratory value, which is unequivocally due to disease progression, should not be reported as an AE/SAE.

Any new or aggravated clinically relevant abnormal medical finding at a physical examination as compared with the baseline assessment will be reported as an AE.

## Warnings and precautions

Additional safety information collected between IB updates will be noted and added to the protocol. This information will be included in the patient informed consent and should be discussed with the patient during the study as needed. The package insert for osimertinib does not contain any specific contraindication.

# Investigational product and other Treatments

## Identity of investigational product(s)

### Agent:

- Other names for the drug(s): AZD9291, Osimertinib or Tagrisso™
- Classification - type of agent: Targeted cancer therapy
- Mode of action: *EGFR TKI.*

Storage and stability: Store osimertinib bottles at 25°C (77°F). Excursions permitted to 15-30°C (59-86°F)

- Protocol dose: 80 mg
- Preparation: 40mg or 80mg film coated tablets
- Route of administration for this study: Oral
- Incompatibilities: none
- Availability: FDA-approved
- Side effects: Please refer to the osimertinib package insert for a comprehensive list of adverse events.

### Return and Retention of Study Drug

The study drug osimertinib will be destroyed by the UT Southwestern designated investigational drug service pharmacy in accordance with institutional protocols. No patient returned drug will be re-assigned to other patients or diverted to another person.

### Study Drug compliance

The patient will be asked to keep a diary of taking the medication, as well as bringing pill bottles to each clinic visit.

## Therapy: Stereotactic Ablation Body Radiation (SABR)

### SABR DOSE AND TECHNIQUES

SABR (also termed stereotactic body radiation therapy [SBRT]) will begin at least 8 weeks after initiation of osimertinib for consolidation of residual disease sites. There is not a window for radiation initiation, however it should be started as soon as possible following 8 weeks of osimertinib. Additional SABR treatments will be delivered to new distinct areas of disease that develop after that period at the discretion of the treating medical oncology/radiation oncology teams.  PFS will at that point already been established, but patients will be followed for OS.

Stereotactic treatment is the targeting, planning, and directing of treatment fields guided to a target based on known 3-D coordinates related to reliable fiducial markers. This differs from conventional radiation therapy in which treatment is guided by skin or bony landmarks assumed to correlate to the target volume based on the initial simulation.  SABR in this study will be delivered with an ablative range of dose per fraction. Treatment will account for inter/intra-fractional errors with careful dosimetry that delivers an ablative dose to the metastatic lesion(s) while respecting normal tissue constraints.

#### Osimertinib during SABR

It is not expected osimertinib and SABR would have significant toxicity if given concurrently. Osimertinib may be held prior to, during and after radiation treatment per the treating investigator’s discretion. SABR Prescription

After 8 weeks of osimertinib, patients will be evaluated by the treating Radiation Oncologist. Based on location of the metastatic lesion(s), dose fractionation will be determined by clinical appropriateness that balances ablation of the lesion(s) while respecting normal tissue constraints.

Prescription Dose

|  | Total Cumulative Dose Encompassing 95% of Planning Target Volume | | |
| --- | --- | --- | --- |
| Number of Fractions | Protocol Compliant | Variation Acceptable | Deviation Unacceptable |
| 1 | 21-27 Gy | <21 Gy but ≥16 Gy | <16 Gy or >27 Gy |
| 3 | 26.5-33 Gy | <26.5 Gy but ≥24.5 Gy | <24.5 Gy or >33 Gy, |
| 5 | 30-37.5 Gy | ≥28 Gy, <30 Gy | <28 Gy or >37.5 Gy, |

Treatment may be delivered on consecutive days with 18 hours between fraction or every other day as deemed appropriate by the treating Radiation Oncologist.

If there is residual disease not amenable to SABR fractionation schemes proposed in the study, especially for bulky hilar or mediastinal disease, a fractionation schema of 45 Gy in 15 fractions may be employed, with adherence to standard normal tissue constraints outlined in the trial.  This treatment will be delivered daily for three weeks.

#### Planning Constraints and Concerns

The tolerance dose of SABR to the gastrointestinal tract is not established, and patients with metastatic disease involving the esophagus, stomach, intestines, or mesenteric lymph nodes will not be eligible. Patients with renal or adrenal metastases are potentially eligible if normal tissue constraints are otherwise met.

Cutaneous metastases are an uncommon manifestation of non-small cell lung cancer that are typically associated with poor prognosis [26]. Patients with cutaneous metastases will be ineligible. As this may represent a group of patients with particularly poor prognosis, again this will be considered within any comparison with historical controls.

It is well established that for palliative effect for a painful bone metastasis, a single dose of 8 Gy is usually as effective as 30 Gy. Long term survival after bone metastasectomy has been reported [27]. Irradiation of non-spinal skeletal sites does not generally require specialized techniques of treatment. Metastases in major lower extremity weight-bearing bones should undergo surgical stabilization if there is plain film evidence of cortical erosion.

Corticosteroid premedication will not be mandated, although it can be used at the discretion of the treating oncologist. Analgesic premedication to avoid general discomfort during long treatment durations is recommended when appropriate.

### Technical Factors

#### Physical Factors

Only photon (x-ray) beams produced by linear accelerators with photon energies of 4-15 MV will be allowed. Cobalt-60 and charged particle beams (including electrons, protons, and heavier ions) are not allowed. Restriction of photon beam energies > 10 MV but less than 15 MV will be based on clinical appropriateness taking into account distance the beam must travel to the target.

### Dose Verification at Treatment

*In-vivo* dosimeter measurements (e.g., diode, TLD) may be obtained for surface dose verification for accessible beams. This information is not required by the protocol.

### Treatment Platforms

The trial allows most commercially available photon producing treatment units except the exclusion of units described in Section 4.3.1 (e.g., cobalt units and charge particle accelerators). Conventional linear accelerators and specialized linear accelerators with image guidance (e.g., Novalis, Trilogy, Synergy, Artiste) are allowed. These units can be used with conformal dose delivery or IMRT. Other specialized accelerators (e.g., the CyberKnife® or Tomotherapy) are allowed as long as they meet the technical specifications of the protocol.

### Simulation/Image Guidance

#### Patient Positioning

Patients will be positioned in a stable position that allows accurate reproducibility of the target between treatments. Positions uncomfortable for the patient should be avoided so as to prevent uncontrolled movement during treatments. A variety of immobilization systems may be utilized including stereotactic frames that surround the patient on three sides and large rigid pillows (conforming to patients external contours) with reference to the stereotactic coordinate system. Patient immobilization must be reliable enough to insure that the Gross Tumor Volume (GTV) does not deviate beyond the confines of the Planning Treatment Volume (PTV) with any significant probability (i.e., < 5%).

At the time of simulation for patients who will receive SABR to the lung and/or liver, the movement of the dome of the diaphragm (superior portion of the liver) is to be observed under fluoroscopy or other acceptable means to estimate respiratory movement during treatment if no breathing control device is used. Patients will be assessed for suitability for tolerance of a respiratory control device using a breath-hold technique, respiratory gating, or abdominal compression to limit diaphragmatic excursion during respiration. Patients with severe lung disease and patients who cannot tolerate diaphragmatic or breathing control devices for other reasons will be treated without them. A larger margin to account for breathing related intra-fractional organ movement is required.

#### Image Guidance

Isocenter or reference point port localization images should be obtained on the treatment unit

immediately before treatment to ensure proper alignment of the geometric center (i.e.,

isocenter) of the simulated fields. These IGRT images can be obtained with planar kV imaging

devices or cone-beam CT equipment. For treatment systems that use kV imaging but also allow EPID imaging using the treatment beam, orthogonal images verifying the isocenter also should be obtained.

## Treatment Planning/Target volumes

### Image Acquisition

Computed tomography will be the primary image platform for targeting and treatment planning. The planning CT scans must allow simultaneous view of the patient anatomy and fiducial system for stereotactic targeting. CT scan with IV contrast is recommended unless the patient has allergy to contrast or renal insufficiency. Oral GI contrast to highlight the stomach and duodenum is recommended for patients with medial liver lesions or lesions of the caudate lobe. Axial acquisitions will be required with spacing ≤ 3.0 mm between scans. Images will be transferred to the treatment planning computers.

### Target Volumes

The target lesion will be outlined by an appropriately trained physician and designated the gross tumor volume (GTV). The target will generally be drawn using appropriate windowing based on location of the metastatic lesion(s). 4-dimensional CT image guided GTV delineation to take tumor motion into consideration will be allowed.

For treatment to the lung, the target will generally be drawn using CT pulmonary windows; however, soft tissue windows with contrast may be used to avoid inclusion of adjacent vessels, atelectasis, or mediastinal or chest wall structures within the GTV. This target will not be enlarged whatsoever for prophylactic treatment (including no “margin” for presumed microscopic extension); rather, include only abnormal CT signal consistent with gross tumor (i.e., the GTV and the clinical target volume [CTV] are identical). An additional 0.5 cm in the axial plane and 1.0 cm in the longitudinal plane (craniocaudal) will be added to the GTV to constitute the PTV.

For treatment to the liver, the following structures are contoured: entire liver, each individual liver gross tumor volume (GTV), each kidney, and the spinal cord. The planning target volume (PTV) is constructed to account for the positional uncertainty of the GTV during treatment. The PTV for each contoured GTV should be at least 5mm larger than the GTV in the axial plane and 1.0 cm larger than the GTV in the craniocaudal plane. Larger margins may be used in cases where greater motion of the hemidiaphragm is observed in simulation despite standard maneuvers to diminish motion.

Treatment to skeletal and paraspinous lesions may be accomplished with any 3D conformal radiotherapy or intensity-modulated radiotherapy technique suitable for this application with performance specifications adequate to provide proper tumor dose distribution and normal tissue sparing.

## Dosimetry

### 3D-Conformal Planning

Three-dimensional coplanar or non-coplanar beam arrangements will be custom designed for each case to deliver highly conformal prescription dose distributions. Non-opposing, non-coplanar beams are preferable. Generally, more beams are used for larger lesion sizes. For this protocol, the isocenter is defined as the common point of gantry and couch rotation for the treatment unit. Prescription lines covering the PTV will typically be the 60-90% line (rather than 95-100%); however, higher isodoses (hotspots) must be manipulated to occur within the target and not in adjacent normal tissue. The isocenter in stereotactic coordinates will be determined from system fiducials (or directly from the tumor in the case of volumetric imaging) and translated to the treatment record.

The treatment dose plan will be made up of multiple static beams or arcs as described above. The plan should be normalized to a defined point corresponding closely to the center of mass of the PTV (COMPTV). Typically, this point will be the isocenter of the beam rotation; however, it is not a protocol requirement for this point to be the isocenter. Regardless, the point identified as COMPTV must have defined stereotactic coordinates and receive 100% of the normalized dose. Because the beam apertures coincide nearly directly with the edge of the PTV (little or no added margin), the external border of the PTV will be covered by a lower isodose surface than usually used in conventional radiotherapy planning typically around 80% but ranging from 60-90%. The prescription dose will be delivered to the margin of the PTV. As such, a “hotspot” will exist within the PTV centrally at the COMPTV with a magnitude of prescribed dose times the reciprocal of the chosen prescription isodose line (i.e., 60-90%).

### Intensity Modulated Radiation Therapy (IMRT)

IMRT is allowed in this study. The use of IMRT in this study is at the discretion of the treating physician. However, IMRT should be considered only when target coverage, OAR dose limits, or dose spillage are not achievable with 3D conformal planning. In addition, IMRT plans should follow the same planning principles as discussed above for 3D conformal planning. The number of segments (control points) and the area of each segment should be optimized to ensure deliverability and avoid complex beam fluences. Ideally, the number of segments should be minimized (2-3 segments per beam should be adequate), and the area of each segment

should be maximized (the aperture of one segment from each beam should correspond to the projection of the PTV along a beam’s eye view).

### Dose Calculations

For purposes of dose planning and calculation of monitor units for actual treatment, this protocol will require tissue density heterogeneity correction.

Successful treatment planning will require accomplishment of all of the following criteria:

Maximum dose: The treatment plan should be created such that 100% corresponds to the maximum dose delivered to the patient. This point must exist within the PTV.

Prescription isodose: The prescription isodose surface must be ≥ 60% and < 90% of the maximum dose.

Prescription Isodose Surface Coverage**:** The prescription isodose surface will be

chosen such that 95% of the target volume (PTV) is conformally covered by the prescription isodose surface (PTV V95%RX = 100%) and 99% of the target volume (PTV) receives a minimum of 90% of the prescription dose (PTV V90%RX > 99%).

### Normal Tissue Dose Constraints

In accordance with the prior Phase I studies [7, 8], certain normal tissue dose constraints must be respected.

The possibility that SABR-induced fibrosis might cause occlusion of large central airways, thus impeding ventilation distal to the occlusion has been well considered. An adjustment to the fractionation scheme may be made if, in the opinion of the treating radiation oncologist, the following conditions apply: (1) the location of a lung lesion is close enough to a large proximal bronchial airway such that occlusion might occur, and (2) compromised ventilation to the segment(s) of lung potentially affected would cause clinically significant adverse consequences.

The same special condition applies in the setting of a patient whose primary lung disease has not been irradiated previously but is present as a PET-positive site of disease, often in proximity to mediastinal structures which is a dose-limiting concern. These patients will be considered by the PI on a case-by-case basis.

The following table lists the specific organ and dose fractionation constraints on normal tissues.

**One Fraction**

| **Serial Tissue** | **Volume** | **Volume Max (Gy)** | **Max Point Dose (Gy)**** | **Endpoint (≥Grade 3)** |
| --- | --- | --- | --- | --- |
| Spinal Cord and medulla | <0.35 cc  <1.2 cc | 10 Gy  8 Gy | 14 Gy | myelitis |
| Spinal Cord Subvolume (5-6 mm above and below level treated per Ryu) | <10% of subvolume | 10 Gy | 14 Gy | myelitis |
| Cauda Equina | <5 cc | 14 Gy | 16 Gy | neuritis |
| Sacral Plexus | <5 cc | 14.4 Gy | 16 Gy | neuropathy |
| Esophagus* | <5 cc | 11.9 Gy | 15.4 Gy | stenosis/fistula |
| Brachial Plexus | <3 cc | 13.6 Gy | 16.4 Gy | neuropathy |
| Heart/Pericardium | <15 cc | 16 Gy | 22 Gy | pericarditis |
| Great vessels | <10 cc | 31 Gy | 37 Gy | aneurysm |
| Trachea and Large Bronchus* | <4 cc | 17.4 Gy | 20.2 Gy | stenosis/fistula |
| Bronchus- smaller airways | <0.5 cc | 12.4 Gy | 13.3 Gy | stenosis with atelectasis |
| Rib | <5 cc | 28 Gy | 33 Gy | Pain or fracture |
| Skin | <10 cc | 25.5 Gy | 27.5 Gy | ulceration |
| Stomach | <5 cc | 17.4 Gy | 22 Gy | ulceration/fistula |
| Bile duct |  |  | 30 Gy | stenosis |
| Duodenum* | <5 cc  <10 cc | 11.2 Gy  9 Gy | 17 Gy | ulceration |
| Jejunum/Ileum* | <30 cc | 12.5 Gy | 22 Gy | enteritis/obstruction |
| Colon* | <20 cc | 18 Gy | 29.2 Gy | colitis/fistula |
| Rectum* | <3.5 cc  <20 cc | 39 Gy  22 Gy | 44.2 Gy | proctitis/fistula |
| Ureter |  |  | 35 Gy | stenosis |
| Bladder wall | <15 cc | 12 Gy | 25 Gy | cystitis/fistula |
| Penile bulb | <3 cc | 16 Gy |  | impotence |
| Femoral Heads | <10 cc | 15 Gy |  | necrosis |
| Renal hilum/vascular trunk | 15 cc | 14 Gy |  | malignant hypertension |
|  |  |  |  |  |
| **Parallel Tissue** | **Critical Volume (cc)** | **Critical Volume Dose Max (Gy)** |  | **Endpoint (≥Grade 3)** |
| Lung (Right & Left) | 1500 cc | 7 Gy |  | Basic Lung Function |
| Lung (Right & Left) | 1000 cc | 7.6 Gy | V-8Gy <37% | Pneumonitis |
| Liver | 700 cc | 11 Gy |  | Basic Liver Function |
| Renal cortex (Right & Left) | 200 cc | 9.5 Gy |  | Basic renal function |

***Avoid circumferential irradiation**

**** “point” defined as 0.035cc or less**

**Three Fractions**

| **Serial Tissue** | **Volume** | **Volume Max (Gy)** | **Max Point Dose (Gy)**** | **Endpoint (≥Grade 3)** |
| --- | --- | --- | --- | --- |
| Spinal Cord and medulla | <0.35 cc  <1.2 cc | 15.9 Gy  13 Gy | 22.5 Gy | myelitis |
| Spinal Cord Subvolume (5-6 mm above and below level treated per Ryu) | <10% of subvolume | 18 Gy | 22.5 Gy | myelitis |
| Cauda Equina | <5 cc | 21.9 Gy | 25.5 Gy | neuritis |
| Sacral Plexus | <5 cc | 22.5 Gy | 24 Gy | neuropathy |
| Esophagus* | <5 cc | 17.7 Gy | 25.2 Gy | stenosis/fistula |
| Brachial Plexus | <3 cc | 22 Gy | 26 Gy | neuropathy |
| Heart/Pericardium | <15 cc | 24 Gy | 30 Gy | pericarditis |
| Great vessels | <10 cc | 39 Gy | 45 Gy | aneurysm |
| Trachea and Large Bronchus* | <5 cc | 25.8 Gy | 30 Gy | stenosis/fistula |
| Bronchus- smaller airways | <0.5 cc | 18.9 Gy | 23.1 Gy | stenosis with atelectasis |
| Rib | <5 cc | 40 Gy | 50 Gy | Pain or fracture |
| Skin | <10 cc | 31 Gy | 33 Gy | ulceration |
| Stomach | <5 cc | 22.5 Gy | 30 Gy | ulceration/fistula |
| Bile duct |  |  | 36 Gy | stenosis |
| Duodenum* | <5 cc  <10 cc | 15.6 Gy  12.9 Gy | 22.2 Gy | ulceration |
| Jejunum/Ileum* | <30 cc | 17.4 Gy | 27 Gy | enteritis/obstruction |
| Colon* | <20 cc | 24 Gy | 34.5 Gy | colitis/fistula |
| Rectum* | <3.5 cc  <20 cc | 45 Gy  27.5 Gy | 49.5 Gy | proctitis/fistula |
| Ureter |  |  | 40 Gy | stenosis |
| Bladder wall | <15 cc | 17 Gy | 33 Gy | cystitis/fistula |
| Penile bulb | <3 cc | 25 Gy |  | impotence |
| Femoral Heads | <10 cc | 24 Gy |  | necrosis |
| Renal hilum/vascular trunk | 15 cc | 19.5 Gy |  | malignant hypertension |
|  |  |  |  |  |
| **Parallel Tissue** | **Critical Volume (cc)** | **Critical Volume Dose Max (Gy)** |  | **Endpoint (≥Grade 3)** |
| Lung (Right & Left) | 1500 cc | 10.5 Gy |  | Basic Lung Function |
| Lung (Right & Left) | 1000 cc | 11.4 Gy | V-11Gy<37% | Pneumonitis |
| Liver | 700 cc | 17.1 Gy |  | Basic Liver Function |
| Renal cortex (Right & Left) | 200 cc | 15 Gy |  | Basic renal function |

***Avoid circumferential irradiation**

**** “point” defined as 0.035cc or less**

**Five Fractions**

| **Serial Tissue** | **Volume** | **Volume Max (Gy)** | **Max Point Dose (Gy)**** | **Endpoint (≥Grade 3)** |
| --- | --- | --- | --- | --- |
| Spinal Cord and medulla | <0.35 cc  <1.2 cc | 22 Gy  15.6 Gy | 28 Gy | myelitis |
| Spinal Cord Subvolume (5-6 mm above and below level treated per Ryu) | <10% of subvolume | 22 Gy | 28 Gy | myelitis |
| Cauda Equina | <5 cc | 30 Gy | 31.5 Gy | neuritis |
| Sacral Plexus | <5 cc | 30 Gy | 32 Gy | neuropathy |
| Esophagus* | <5 cc | 19.5 Gy | 35 Gy | stenosis/fistula |
| Brachial Plexus | <3 cc | 27 Gy | 32.5 Gy | neuropathy |
| Heart/Pericardium | <15 cc | 32 Gy | 38 Gy | pericarditis |
| Great vessels | <10 cc | 47 Gy | 53 Gy | aneurysm |
| Trachea and Large Bronchus* | <5 cc | 32 Gy | 40 Gy | stenosis/fistula |
| Bronchus- smaller airways | <0.5 cc | 21 Gy | 33 Gy | stenosis with atelectasis |
| Rib | <5 cc | 45 Gy | 57 Gy | Pain or fracture |
| Skin | <10 cc | 36.5 Gy | 38.5 Gy | ulceration |
| Stomach | <5cc | 26.5 Gy | 35 Gy | ulceration/fistula |
| Bile duct |  |  | 41 Gy | stenosis |
| Duodenum* | <5 cc  <10 cc | 18.5 Gy  14.5 Gy | 26 Gy | ulceration |
| Jejunum/Ileum* | <30 cc | 20 Gy | 32 Gy | enteritis/obstruction |
| Colon* | <20 cc | 28.5 Gy | 40 Gy | colitis/fistula |
| Rectum* | <3.5 cc  <20 cc | 50 Gy  32.5 Gy | 55 Gy | proctitis/fistula |
| Ureter |  |  | 45 Gy | stenosis |
| Bladder wall | <15 cc | 20 Gy | 38 Gy | cystitis/fistula |
| Penile Bulb | <3 cc | 30 Gy |  | impotence |
| Femoral Heads | <10 cc | 30 Gy |  | necrosis |
| Renal hilum/vascular trunk | 15 cc | 23 Gy |  | malignant hypertension |
|  |  |  |  |  |
| **Parallel Tissue** | **Critical Volume (cc)** | **Critical Volume Dose Max (Gy)** |  | **Endpoint (≥Grade 3)** |
| Lung (Right & Left) | 1500 cc | 12.5 Gy |  | Basic Lung Function |
| Lung (Right & Left) | 1000 cc | 13.5 Gy | V-13.5Gy<37% | Pneumonitis |
| Liver | 700 cc | 21 Gy |  | Basic Liver Function |
| Renal cortex (Right & Left) | 200 cc | 18 Gy |  | Basic renal function |

***Avoid circumferential irradiation**

**** “point” defined as 0.035cc or less**

Exceeding these dose tolerances by more than 2.5% constitutes a minor protocol violation. Exceeding these dose tolerances by more than 5% constitutes a major protocol violation.

## Intracranial Stereotactic Radiosurgery Dose (SRS) and Technique

Patients with up to 10 brain metastases with the maximal size ≤ 4cm may be treated by SRS in a single fraction. If there are more than 10 intracranial lesions or the size of the largest lesion is >4cm, such patients will be ineligible.

### SRS Dose

All supratentorial lesions are treated to the prescribed dose below.

|  | **SRS Dose by lesion diameter** | | |
| --- | --- | --- | --- |
| Diameter (cm) | ≤ 2.0 cm | >2.0 - 3.0 cm | >3.0 - 4.0 cm |
| Dose (Gy) | 20-24 | 18 | 15 |

For lesions within the brainstem (pons, medulla, and midbrain), the prescription dose will be 10-13 Gy in a single fraction.

Multiple SRS sessions are allowed, as long as all intracranial lesions are treated within 14 days.

### SRS Technique, Pre-SRS Image acquisition and target planning with Conformality Index

Protocol SRS will be administered with a linear accelerator (LINAC), Vero ® (BrainLab, Feldkirchen, Germany), CyberKnife ® (Accuray, Sunnyvale, CA) or Gamma Knife® Perfexion (Elektra Instruments, Inc., Atlanta, GA). Fixed-frame stereotactic localization and planning will be used for SRS.

Pre-SRS Image Acquisition: MRI with gadolinium contrast is the recommended imaging method for stereotactic localization. In the case of LINAC, Vero or CyberKnife, MRI with contrast has to be obtained within 14 days of SRS with an acquisition of <= 1.5 mm slice thickness. The stereotactic target is the contrast-enhancing tumor as defined on MRI with contrast.

In patients who cannot tolerate the contrast, other MR sequences may be utilized without the contrast, as long as the target lesion can be clearly demarcated for treatment and follow up.

Conformality Index (CI) is derived from dividing the volume encompassed by the prescription isodose line by the target volume.

CI = Rx Isodose Vol / (Target Vol)

The target lesion in the SRS planning MRI will be contoured, and its volume will be calculated. Planning should achieve a conformity index specified below.

| Size of Target | Prescription Isodose | Conformality Index |
| --- | --- | --- |
| ≤1cm | 35-100% | < 2 |
| 1-2cm |  | < 2 |
| 2-3cm |  | < 1.5 |
| 3-4cm |  | < 1.25 |

At least 95% of the target volume must receive the prescription dose; the plan should be adjusted to achieve such coverage by the isodose line between 40-100%. No part of the target should receive less than 90% of the prescription dose.

### Normal Tissue Dose Constraints

The following table lists maximum dose limits, in one fraction, to a point or volume within several critical structures of the cranium. These are absolute limits, and treatment delivery that exceeds these limits will constitute a major protocol violation.

| **Critical Structure and margin** | **Volume** | **Volume Max (Gy)** | **Max Point Dose (Gy)*** | **Endpoint (≥Grade 3)** |
| --- | --- | --- | --- | --- |
| Optic Pathway | <0.2 cc | 8 Gy | 10 Gy | neuritis |
| Brainstem (not medulla) | <0.5 cc | 10 Gy | 15 Gy | cranial neuropathy |
| Spinal Cord and medulla | <0.35cc  <1.2 cc | 10 Gy  8 Gy | 14 Gy | myelitis |

* “point” defined as 0.035cc or less

Patients with lesions within or near the above structures and the dose constraints cannot be satisfied, will be deemed ineligible.

## Radiation Therapy Quality Assurance

The radiation oncologist will perform an RT Quality Assurance Review after complete data for the first half of cases (18) enrolled at the University of Texas Southwestern Medical Center. They will perform the final review after complete data for the subsequent 19 cases at the University of Texas Southwestern Medical Center are completed. These cases will be reviewed within 3 months after this study has reached the target accrual or as soon as complete data for all cases enrolled has been received, whichever occurs first.

# STATISTICAL CONSIDERATIONS

## Study Design/Study Endpoints

**Study Design:**

This is an, open-label, single arm protocol designed to evaluate the activity of targeted therapy and SABR in advanced *EGFR* mutant NSCLC who have not been treated previously with EGFR targeted therapy.

Osimertinib will be administered to the patient until disease progression by RECIST 1.1 (The investigator may continue osimertinib post-progression if deemed to still be clinically beneficial), unacceptable toxicity, withdrawal of consent, or discontinuation of the trial for any other reason including death.

**Purpose:** of this prospective, multi-center, single arm, non-randomized trial is to evaluate the impact of the combination of osimertinib and stereotactic radiation delivered to patients with *EGFR mutated* adenocarcinoma of the lung. The enhanced efficacy of this combination is to be evaluated specifically with regards to prolongation of clinical benefit of osimertinib. We will also evaluate the safety of this combination of targeted therapy and radiation.

For patients started on osimertinib and treated with SABR to persisting lesions:

PFS compared to published historical results with osimertinib alone PFS=19.3 months for no previous osimertinib therapy ***[6]*** PFS will be defined as time from first dose of osimertinib to date of objective progression (as determined by the investigator according to RECIST 1.1) or death (in the absence of progression).

As secondary objectives, please refer to section 2.2 we will report, duration of response (DoR), objective response rate (ORR), overall survival (OS), time to subsequent SABR or death, time to osimertinib discontinuation or death. We will also be describing toxicity and AEs and then evaluating them in the context of historical controls.

## Sample Size and Accrual

The results of a Phase I trial of osimertinib in advanced *EGFR* mutant NSCLC were presented at the ESMO 2016 conference by Ramalingam et al. Osimertinib demonstrated activity in patients with an overall response rate of 77% at doses of 80 mg or 160 mg daily. Median progression free survival (PFS) was 19.3 months for the 160 mg dose and was not reached for the 80 mg dose.

The sample size calculation is based on the primary endpoint, progression-free survival, and the assumption that patients are enrolled until the end of accrual.

A sample size of 43 patients achieves at least 80% power to detect the difference between the null hypothesis of median PFS of 19.3 months and the alternative hypothesis of median PFS of 30 months at one-sided significance level of 0.1. We assume the patient accrual period of 36 months and the follow-up period of 12 months. The assumption is that patients are enrolled until the end of accrual. The sample size was estimated using SWOG (https://www.swogstat.org/stat/public/one_survival.htm) sample size calculator.

## Data Analyses Plans

We will use Kaplan-Meier methods to estimate the progression-free survival, time to subsequent SABR and overall survival, clinical benefit rate, time to treatment discontinuation duration of response and time to new metastases.

Duration of response (defined in 6.1.5) duration will be summarized for patients who responded. Descriptive summary statistics like median, 25% and 75% percentiles will be used.

Toxicities are dichotomized as none versus any, or none and mild versus moderate to severe (based on CTCAE grades).

The rates of overall response, toxicity and adverse events as well as their 95% confidence intervals will be estimated using exact binomial method. We will compare the rates of toxicity and adverse events in this study with those of historical controls from the previously referenced trial by Ramalingam et al, by the Fisher's exact test.

## Analysis sets

### Full Analysis Set

The Full Analysis Set (FAS) comprises all enrolled patients.

### Safety Set

The Safety Set includes all patients who received at least one dose of osimertinib medication.

### Patient demographics/other baseline characteristics

Demographic, disease characteristics and other baseline data will be summarized descriptively for the FAS.

### Treatments (osimertinib treatment, concomitant therapies, compliance)

All analyses from this section will be performed on all patients from the safety set. Duration of osimertinib treatment exposure will be summarized.

### Handling of missing values/censoring/discontinuations

All attempts will be made to ensure that the database contains full information for the safety set.

### Safety objectives

The safety objective is to describe toxicities in patients treated with osimertinib.

### Analysis set and grouping for the analyses

For all safety analyses, the safety set will be used. Toxicities will be dichotomized as none versus any adverse event, or none and mild versus moderate to severe adverse event.

The safety summary tables will include assessments from the on-treatment period, unless otherwise specified.

All safety data collected in the study will be listed regardless of the study period with data collected during the pre-treatment and post-treatment period flagged.

### Adverse events (AEs)

Summary tables for adverse events (AEs) will include only AEs that started or worsened during the on-treatment period, the treatment-emergent AEs. However, all safety data (including those from the pre and post-treatment periods) will be listed and those collected during the pre-treatment and post-treatment period are to be flagged.

The incidence of treatment-emergent adverse events (new or worsening from baseline) will be summarized by system organ class and or preferred term, severity (based on CTCAE grades), type of adverse event, relation to study treatment by

Deaths reportable as SAEs and non-fatal serious adverse events will be listed by patient and tabulated by type of adverse event and.

Specific safety event categories (SEC) will be considered. Such categories consist of one or more well-defined safety events which are similar in nature and for which there is a specific clinical interest in connection with the study treatment(s).

For each specified category, number and percentage of patients with at least one event per category will be summarized.

### Other safety data

Other safety data (including ECGs, vital signs and weight) will be summarized and listed, notable values will be flagged, and any other information collected will be listed as appropriate.

### Treatment phase and duration of treatment

Patients eligible for treatment will receive 80 mg daily of osimertinib as part of a 28-day cycle. The first dose of each cycle will be administered after evaluation at the study center. Patients will remain on study and take osimertinib until there is evidence of disease progression by RECIST, unacceptable toxicity, withdrawal of consent, or discontinuation of the trial for any other reason.

Continuation of osimertinib will be permitted if the treating oncologist feels the patient would benefit. If there is oligoprogression amenable to SABR, resumption of osimertinib after SABR will be permitted also if the treating oncologist feels the patient would benefit.

## Definition of end of the Study

The study will be continued until all 43 patients have evidence of disease progression by RECIST not amenable to SABR requiring discontinuation of osimertinib, unacceptable toxicity, withdrawal of consent, or discontinuation of the trial for any other reason to a maximum of 10 years.

At the completion or discontinuation of study medication, all patients will be seen within 30 days for an end of therapy evaluation. This will include a safety assessment for AE’s SAE’s. Any unused medication will be returned. Patients will be followed post study to collect survival data every 3 months for a maximum of 10 years.

## Early Termination

Treatment on protocol may be terminated early if the Principal investigator or institution assess that the safety of the enrolled subjects will be compromised by continuation of the trial. The procedure followed will be that of the premature withdrawal patient and any patients on treatment will be seen as soon as possible.

## Data confidentiality

Information about protocol subjects will be kept confidential and managed under the applicable laws and regulations. Those regulations require a signed subject authorization informing the subject of the following:

- What protected health information (PHI) will be collected from subjects in this protocol

- Who will have access to that information and why

- Who will use or disclose that information

- The rights of a research subject to revoke their authorization for use of their PHI.

In the event that a subject revokes authorization to collect or use PHI, the investigator, by

regulation, retains the ability to use all information collected prior to the revocation of subject authorization. For subjects that have revoked authorization to collect or use PHI, attempts should be made to obtain permission to collect follow-up safety information (e.g. has the subject experienced any new or worsened AEs) at the end of their scheduled protocol treatment period.

# Ethical considerations and administrative procedures

## Regulatory and ethical compliance

This clinical study was designed, shall be implemented and reported in accordance with the ICH Harmonized Tripartite Guidelines for Good Clinical Practice, with applicable local regulations (including European Directive 2001/20/EC and US Code of Federal Regulations Title 21), and with the ethical principles laid down in the Declaration of Helsinki.

## Informed consent procedures

Eligible patients will only be included on this after providing written IRB/IEC/REB-approved informed consent.

Informed consent must be obtained before conducting any protocol-specific procedures (i.e. all of the procedures described in the protocol). However, the Investigator should not repeat procedures that are performed as part of standard of care (SOC), if they are within the screening window and are done prior to signing the ICF. The process of obtaining informed consent should be documented in the patient source documents. The date when a subject’s Informed Consent was actually obtained will be captured in the clinical trial record. The informed consent document will be approved by the IRB.

## Discontinuation of the study

This study will discontinue if terminated by the Institutional Review Board or at the discretion of the Principal Investigator.

## Publication of the study and results

The results of this study will be updated and posted per regulatory requirements, including (but not limited) to databases such as clinicaltrials.gov.

### Communication and Publication of Clinical Trial Results

All submitted manuscripts will comply with institutional guidelines and with authorship guidelines of the International Committee of Medical Journal Editors.

## Study documentation, record keeping and retention of documents

The investigator/institution should maintain the trial documents as specified in Essential Documents for the Conduct of a Clinical Trial (ICH E6 Section 8) and as required by applicable regulations and/or guidelines. The investigator/institution should take measures to prevent accidental or premature destruction of these documents.

## Confidentiality of study documents and patient records

The investigator must ensure anonymity of the patients; patients must not be identified by names in any trial documents. Signed informed consent forms and patient enrollment log must be kept strictly confidential to enable patient identification.

## Audits and inspections

Source data/documents must be available to inspections by Health Authorities.

# STUDY MANAGEMENT

## Conflict of Interest (COI)

Any investigator who has a conflict of interest (COI) with this study (patent ownership, royalties, or financial gain greater than the minimum allowable by their institution, etc.) must have the conflict reviewed by the UTSW COI Committee and IRB according to UTSW Policy on Conflicts of Interest. All investigators will follow the University conflict of interest policy.

## Institutional Review Board (IRB) Approval and Consent

It is expected that the IRB of record will have the proper representation and function in accordance with federally mandated regulations. The IRB of record must approve the consent form and protocol.

In obtaining and documenting informed consent, the investigator should comply with the applicable regulatory requirement(s), and should adhere to Good Clinical Practice (GCP) and to ethical principles that have their origin in the Declaration of Helsinki.

Before recruitment and enrollment onto this study, the subject will be given a full explanation of the study and will be given the opportunity to review the consent form. Each consent form must include all the relevant elements currently required by the FDA Regulations and local or state regulations. Once this essential information has been provided to the subject and the investigator is assured that the subject understands the implications of participating in the study, the subject will be asked to give consent to participate in the study by signing an IRB‑approved consent form.

Prior to a patient’s participation in the trial, the written informed consent form should be signed and personally dated by the subject and by the person who conducted the informed consent discussion.

**11.3 Required Documentation (for multi-site studies)**

Before the study can be initiated at any sub-site, the following documentation must be provided to the [RESEARCH OFFICE].

- A copy of the official IRB approval letter for the protocol and informed consent
- IRB membership list or Federal-wide Assurance letter
- CVs and medical licensure for the principal investigator and any associate investigators who will be involved in the study
- Form FDA 1572 appropriately filled out and signed with appropriate documentation (NOTE: this is required if UT Southwestern PI holds an IND. Otherwise, the sub-site Investigator’s signature on the protocol is sufficient to ensure compliance.)
- A copy of the IRB‑approved consent form
- CAP and CLIA Laboratory certification numbers and institution lab normal values
- Executed clinical research contract
- Signed Monitoring Plan

## Registration Procedures

For UT Southwestern Medical Center:

The coordinating center for the trial will be UT Southwestern. Patients who are candidates for registration into the study will be evaluated for eligibility by the Investigator once consent has been obtained to ensure that the inclusion and exclusion criteria have been satisfied and that the patient is eligible for participation in this clinical study. The site should maintain a log of all patients who are consented. This log will include patients who are enrolled and patients who are consented but do not qualify for the study or who do not receive study drug. The reason for disqualification should be documented on the log.

For Sub-Site Management:

The coordinating center for the trial will be UT Southwestern. Patients who are candidates for registration into the study will be approved by UTSW after confirmation of eligibility of the inclusion and exclusion criteria have been satisfied. UTSW will confirm eligibility for all patients prior to receipt of the first dose of study drug.

For patients who pass screening the following items will be required for registration:

- copy of the signed patient treatment consent
- completed patient eligibility verification registration form
- completed inclusion/exclusion checklist
- source documentation (which includes, but not limited to, pathology, labs, progress notes, etc) that confirms all eligibility items on the inclusion-exclusion checklist has been met

This information will be provided no later than 48 hours prior to the planned cycle 1 day 1 treatment to allow adequate time for review. These items can be faxed or emailed to:

**Jessica Saltarski**

**UT Southwestern**

**fax 214-648-1906**

or scanned then emailed to [**jessica.saltarski@utsouthwestern.edu**](mailto:jessica.saltarski@utsouthwestern.edu). The Lung DOT manager will complete the patient ID assignment on the patient eligibility verification registration form and provide this completed form back within 48 hours of receipt of the package.

The site should maintain a log of all patients who are consented but do not qualify for the study or who do not receive study drug. The reason for disqualification should be documented on the log.

## Data Management and Monitoring/Auditing

REDCap is the UTSW SCCC institutional choice for the electronic data capture of case report forms for this and all SCCC Investigator Initiated Trials. REDCap will be used for electronic case report forms in accordance with Simmons Comprehensive Cancer Center requirements, as appropriate for the project.

All sites are expected to refer to the electronic case report forms to ensure required study-specific data is collected and verifiable in appropriate source documents.

In order to facilitate remote source to case report form verification, the Simmons Comprehensive Cancer Center study team will require other institutions participating in this trial as sub-sites to enter data into the selected EDC system and upload selected de-identified source materials when instructed

Trial monitoring will be conducted no less than annually and refers to a regular interval review of trial related activity and documentation performed by the DOT and/or the CRO Multi-Center IIT Monitor. This review includes but is not limited to accuracy of case report forms, protocol compliance, timeless and accuracy of Velos entries and AE/SAE management and reporting. Documentation of trial monitoring will be maintained along with other protocol related documents and will be reviewed during internal audit.

**11.5.3 Trial Audits**

Toxicity, dose escalation, and safety lead-in audits will be performed by the SCCC DSMC at least monhtly

For further information, refer to the UTSW SCCC IIT Management Manual.

The UTSW Simmons Comprehensive Cancer Center (SCCC) Data Safety Monitoring Committee (DSMC) is responsible for monitoring data quality and patient safety for all UTSW SCCC clinical trials. As part of that responsibility, the DSMC reviews all local serious adverse events and UPIRSOs in real time as they are reported and reviews adverse events on a quarterly basis. The quality assurance activity for the Clinical Research Office provides for periodic auditing of clinical research documents to ensure data integrity and regulatory compliance. A copy of the DSMC plan is available upon request.

## The SCCC DSMC meets quarterly and conducts annual comprehensive reviews of ongoing clinical trials, for which it serves as the DSMC of record. The QAC works as part of the DSMC to conduct regular audits based on the level of risk. Audit findings are reviewed at the next available DSMC meeting. In this way, frequency of DSMC monitoring is dependent upon the level of risk. Risk level is determined by the DSMC Chairman and a number of factors such as the phase of the study; the type of investigational agent, device or intervention being studied; and monitoring required to ensure the safety of study subjects based on the associated risks of the study. Protocol-specific DSMC plans must be consistent with these principles.

## Adherence to the Protocol

Except for an emergency situation in which proper care for the protection, safety, and well-being of the study subject requires alternative treatment, the study shall be conducted exactly as described in the approved protocol.

Any deviation from the protocol requirements, whether pre-approved or unexpected, are to be recorded/logged as a protocol deviation and reported per institutional policy.

### Exceptions (also called single-subject exceptions or single-subject waivers): include any departure from IRB-approved research that is *not due to an emergency* and is:

- intentional on part of the investigator; and/or
- in the investigator’s control; and/or
- not intended as a systemic change (e.g., single-subject exceptions to eligibility [inclusion/exclusion] criteria)

⮚ **Reporting requirement**: Exceptions are non-emergency deviations that require ***prospective*** IRB approval before being implemented. Call the IRB if your request is urgent. If IRB approval is not obtained beforehand, this constitutes a major deviation.

- **Multicenter IIT Reporting requirement*:** Exceptions are non-emergency

Deviations that require prospective approval from the UTSW Sponsor-Investigator and IRB of record before being implemented. If approval is not obtained beforehand from both UTSW Sponsor-Investigator and IRB of record, this constitutes a major deviation. For eligibility waivers, studies which utilize the SCCC-DSMC as the DSMC of record must also obtain approval from the SCCC DSMC prior to submitting to the IRB of record for approval. For Non-emergency exceptions and eligibility waivers, also notify the IIT Multicenter Project Manager (or designee).

### Emergency Deviations: include any departure from IRB-approved research that is necessary to:

### avoid immediate apparent harm, or

### protect the life or physical well-being of subjects or others

⮚ **Reporting requirement**: Emergency deviations must be promptly reported

to the IRB within 5 working days of occurrence.

⮚ **Multicenter IIT Reporting requirement***: Emergency deviations must be

promptly reported to the UTSW Sponsor-Investigator, IIT Multicenter Project Manager (or designee), and IRB of record per institutional policy. For studies conducted at UTSW, report emergency deviations to the UTSW IRB within 5 working days of occurrence.

### Serious Noncompliance (formerly called major deviations or violations): include any departure from IRB-approved research that::

- Increase risk of harm to subjects; and/or adversely affects the rights, safety, or welfare of subjects (any of which may also be an unanticipated problem); and/or
- adversely affects the integrity of the data and research (i.e., substantially compromises the integrity, reliability, or validity of the research)

⮚ **Reporting requirement***: Serious Noncompliance must be promptly reported to the IRB within 5 working days of discovery.

**Multicenter IIT Reporting requirement***: Serious Noncompliance must be promptly reported to the UTSW Sponsor-Investigator, IIT Multicenter Project Manager (or designee), and IRB of record per institutional policy. For studies conducted at UTSW, report Serious Noncompliance to the UTSW IRB within 5 working days of discovery.

**Continuing Noncompliance:** includes a pattern of repeated noncompliance (in one or more protocols simultaneously, or over a period of time) which continues **after** initial discovery, including inadequate efforts to take or implement corrective or preventive action within a reasonable time frame.

⮚ **Reporting requirement***: Continuing Noncompliance must be promptly reported to the IRB within 5 working days of discovery.

⮚ **Multicenter IIT Reporting requirement***: Continuing Noncompliance must

be promptly reported to the UTSW Sponsor-Investigator, IIT Multicenter

Project Manager (or designee), and IRB of record per institutional policy. For

studies conducted at UTSW, report Continuing Noncompliance to the UTSW

IRB within 5 working days of discovery.

**Noncompliance (that is neither serious nor continuing; formerly called minor deviations) any departure from IRB-approved research that:**

- Does not meet the definition of serious noncompliance or continuing noncompliance

⮚ **Reporting requirement***: Noncompliance that is neither serious nor continuing should be tracked and summarized at the next IRB continuing review, or the notice of study closure, whichever comes first.

⮚ **Multicenter IIT Reporting requirement***: Noncompliance that is neither serious nor continuing should be reported to the IRB of record per institutional policy. For studies conducted at UTSW, report Noncompliance that is neither serious nor continuing at the next IRB continuing review, or the notice of study closure, whichever comes first.

## Amendments to the Protocol

Should amendments to the protocol be required, the amendments will be originated and documented by the Principal Investigator. A summary of changes document outlining proposed changes as well as rationale for changes, when appropriate, is highly recommended. When an amendment to the protocol substantially alters the study design or the potential risk to the patient, a revised consent form might be required.

The written amendment, and if required the amended consent form, must be sent to the IRB for approval prior to implementation.

## Record Retention

Study documentation includes all Case Report Forms, data correction forms or queries, source documents, Sponsor-Investigator correspondence, monitoring logs/letters, and regulatory and essential documents (e.g., protocol and amendments, IRB correspondence and approval, signed patient consent forms).

Source documents include all recordings of observations or notations of clinical activities and all reports and records necessary for the evaluation and reconstruction of the clinical research study.

Government agency regulations and directives require that the study investigator retain all study documentation pertaining to the conduct of a clinical trial. In the case of a study with a drug seeking regulatory approval and marketing, these documents shall be retained for at least two years after the last approval of marketing application in an International Conference on Harmonization (ICH) region. In all other cases, study documents should be kept on file until three years after the completion and final study report of this investigational study.

For multi-center studies, UTSW and all sub-sites are to obtain approval from the Sponsor-Investigator prior to destruction of any original study documentation or original source documents.

## Obligations of Investigators

The Principal Investigator is responsible for the conduct of the clinical trial at the site in accordance with Title 21 of the Code of Federal Regulations and/or the Declaration of Helsinki. The Principal Investigator is responsible for personally overseeing the treatment of all study patients. The Principal Investigator must assure that all study site personnel, including sub-investigators and other study staff members, adhere to the study protocol and all FDA/GCP/NCI regulations and guidelines regarding clinical trials both during and after study completion.

The Principal Investigator at each institution or site will be responsible for assuring that all the required data will be collected and entered onto the Case Report Forms. Periodically, monitoring visits may be conducted and the Principal Investigator will provide access to his/her original records to permit verification of proper entry of data. At the completion of the study, all case report forms will be reviewed by the Principal Investigator and will require his/her final signature to verify the accuracy of the data.

### Source data

Each participating site will maintain appropriate medical and research records for this trial, in compliance with Section 4.9 of ICH E6 GCP, and regulatory and institutional requirements for the protection of confidentiality of patients.

Source data include all information, original records of clinical findings, observations, or other activities in a clinical trial necessary for the reconstruction and evaluation of the trial. Examples of these original documents and data records include, but are not limited to, hospital records, clinical and office charts, laboratory notes, memoranda, evaluation checklists, pharmacy dispensing records, recorded data from automated instruments, copies or transcriptions certified after verification as being accurate and complete, microfiches, photographic negatives, microfilm or magnetic media, x-rays, and subject files and records kept at the pharmacy, at the laboratories, and medico-technical departments involved in the clinical trial.

Data collection is the responsibility of the clinical trial staff at the site under the supervision of the site Investigator. The study CRF is the primary data collection instrument for the study. The investigator should ensure the accuracy, completeness, and timeliness of the data reported in the CRFs and all other required reports. Data reported on the CRFs, which are derived from source documents, should be consistent with the source documents or the discrepancies should be explained. All data requested on the CRF must be recorded. Any missing data must be explained.

### Study agreements

Agreements between AstraZeneca and the Investigator Sponsor should be in place before any study-related procedures can take place, or subjects are enrolled.

# References (available upon request)

1. Ferlay, J., et al., *Estimates of worldwide burden of cancer in 2008: GLOBOCAN 2008.* Int J Cancer, 2010. **127**(12): p. 2893-917.

2. Siegel, R., D. Naishadham, and A. Jemal, *Cancer statistics for Hispanics/Latinos, 2012.* CA Cancer J Clin, 2012. **62**(5): p. 283-98.

3. Midha, A., S. Dearden, and R. McCormack, *EGFR mutation incidence in non-small-cell lung cancer of adenocarcinoma histology: a systematic review and global map by ethnicity (mutMapII).* Am J Cancer Res, 2015. **5**(9): p. 2892-911.

4. Rosell, R., et al., *Erlotinib versus standard chemotherapy as first-line treatment for European patients with advanced EGFR mutation-positive non-small-cell lung cancer (EURTAC): a multicentre, open-label, randomised phase 3 trial.* Lancet Oncol, 2012. **13**(3): p. 239-46.

5. Langer, C.J., *Epidermal growth factor receptor inhibition in mutation-positive non-small-cell lung cancer: is afatinib better or simply newer?* J Clin Oncol, 2013. **31**(27): p. 3303-6.

6. Ramalingam, S., et al., *LBA1_PR: Osimertinib as first-line treatment for EGFR mutation-positive advanced NSCLC: updated efficacy and safety results from two Phase I expansion cohorts.* J Thorac Oncol, 2016. **11**(4 Suppl): p. S152.

7. Tang, Z.H., et al., *Characterization of osimertinib (AZD9291)-resistant non-small cell lung cancer NCI-H1975/OSIR cell line.* Oncotarget, 2016. **7**(49): p. 81598-81610.

8. Zhang, X.Y., et al., *Osimertinib (AZD9291), a Mutant-Selective EGFR Inhibitor, Reverses ABCB1-Mediated Drug Resistance in Cancer Cells.* Molecules, 2016. **21**(9).

9. Chen, Z., et al., *Osimertinib (AZD9291) Enhanced the Efficacy of Chemotherapeutic Agents in ABCB1- and ABCG2-Overexpressing Cells In Vitro, In Vivo, and Ex Vivo.* Mol Cancer Ther, 2016. **15**(8): p. 1845-58.

10. Iyengar, P., et al., *Phase II trial of stereotactic body radiation therapy combined with erlotinib for patients with limited but progressive metastatic non-small-cell lung cancer.* J Clin Oncol, 2014. **32**(34): p. 3824-30.

11. Shepherd, F.A., et al., *Erlotinib in previously treated non-small-cell lung cancer.* N Engl J Med, 2005. **353**(2): p. 123-32.

12. Goldie, J.H. and A.J. Coldman, *The genetic origin of drug resistance in neoplasms: implications for systemic therapy.* Cancer Res, 1984. **44**(9): p. 3643-53.

13. Foo, J. and F. Michor, *Evolution of acquired resistance to anti-cancer therapy.* J Theor Biol, 2014. **355**: p. 10-20.

14. Rusthoven, K.E., et al., *Is there a role for consolidative stereotactic body radiation therapy following first-line systemic therapy for metastatic lung cancer? A patterns-of-failure analysis.* Acta Oncol, 2009. **48**(4): p. 578-83.

15. Janne, P.A., et al., *AZD9291 in EGFR inhibitor-resistant non-small-cell lung cancer.* N Engl J Med, 2015. **372**(18): p. 1689-99.

16. Cross, D.A., et al., *AZD9291, an irreversible EGFR TKI, overcomes T790M-mediated resistance to EGFR inhibitors in lung cancer.* Cancer Discov, 2014. **4**(9): p. 1046-61.

17. Hirano, T., et al., *In vitro modeling to determine mutation specificity of EGFR tyrosine kinase inhibitors against clinically relevant EGFR mutants in non-small-cell lung cancer.* Oncotarget, 2015. **6**(36): p. 38789-803.

18. Greig, S.L., *Osimertinib: First Global Approval.* Drugs, 2016. **76**(2): p. 263-73.

19. NCCN-NSCL;, Clinical Practice Guidelines, non small cell lung cancer 2015. [**http://www.nccn.org/professionals/physician_gls/pdf/nscl.pdf**](http://www.nccn.org/professionals/physician_gls/pdf/nscl.pdf).

20. Riely, G.J., et al., *Prospective assessment of discontinuation and reinitiation of erlotinib or gefitinib in patients with acquired resistance to erlotinib or gefitinib followed by the addition of everolimus.* Clin Cancer Res, 2007. **13**(17): p. 5150-5.

21. Nishie, K., et al., *Epidermal growth factor receptor tyrosine kinase inhibitors beyond progressive disease: a retrospective analysis for Japanese patients with activating EGFR mutations.* J Thorac Oncol, 2012. **7**(11): p. 1722-7.

22. Maruyama, R., et al., *Treatment after the failure of gefitinib in patients with advanced or recurrent non-small cell lung cancer.* Anticancer Res, 2009. **29**(10): p. 4217-21.

23. Asami, K., et al., *Continued treatment with gefitinib beyond progressive disease benefits patients with activating EGFR mutations.* Lung Cancer, 2013. **79**(3): p. 276-82.

24. Song, H.N., et al., *Acquired C797S Mutation upon Treatment with a T790M-Specific Third-Generation EGFR Inhibitor (HM61713) in Non-Small Cell Lung Cancer.* J Thorac Oncol, 2016. **11**(4): p. e45-7.

25. Planchard, D., et al., *EGFR-independent mechanisms of acquired resistance to AZD9291 in EGFR T790M-positive NSCLC patients.* Ann Oncol, 2015. **26**(10): p. 2073-8.

26. Potters, L., et al., *American Society for Therapeutic Radiology and Oncology (ASTRO) and American College of Radiology (ACR) practice guideline for the performance of stereotactic body radiation therapy.* Int J Radiat Oncol Biol Phys, 2010. **76**(2): p. 326-32.

27. Hasselle, M.D., et al., *Hypofractionated image-guided radiation therapy for patients with limited volume metastatic non-small cell lung cancer.* J Thorac Oncol, 2012. **7**(2): p. 376-81.

# Appendices

**Guidance regarding Potential Interactions With Concomitant Medications**

The use of any natural/herbal products or other “folk remedies” should be discouraged, but use of these products, as well as use of all vitamins, nutritional supplements, and all other concomitant medications must be recorded in the eCRF.

**Drugs inducing CYP3A4 metabolism that AstraZeneca strongly recommend are not combined with study treatment**

Osimertinib is metabolized by CYP3A4 and CYP3A5 enzymes.

A drug-drug interaction study of osimertinib evaluated in patients showed that there is potential for osimertinib being a victim when co-administered with strong inducers of CYP3A4 (osimertinib concentrations are decreased when co-dosed with rifampicin).

The following potent inducers of CYP3A4 should not be used during this study for any patient receiving study treatment.

| **Table 1 Drugs inducing CYP3A4** | |
| --- | --- |
| **Contraindicated drugs** | **Withdrawal period prior to Study treatment start** |
| Carbamazepine, phenobarbital, phenytoin, rifampicin, rifabutin, rifapentin  St John’s Wort | 3 weeks |
| Phenobarbitone | 5 weeks |

This list is not intended to be exhaustive, and a similar restriction will apply to other agents that are known to strongly modulate CYP3A4 activity. Appropriate medical judgment is required. Please contact AstraZeneca with any queries you have on this issue.

**Medicines whose exposures may be affected by osimertinib that AstraZeneca considers may be allowed with caution**

Osimertinib may increase the concentration of sensitive BCRP and Pgp substrates (concentration of the sensitive BCRP substrate, rosuvastatin and sensitive Pgp substrate, fexofenadine, are increased).

| **Table 2 Exposure, pharmacological action and toxicity may be increased by Osimertinib** | |
| --- | --- |
| **Warning of possible interaction** | **Advice** |
| Rosuvastatin | Drugs are permitted but caution should be exercised and patients monitored closely for possible drug interactions. Please refer to full prescribing information for all drugs prior to co-administration with Study treatment. |
| Sulfasalazine |  |
| Doxorubicin |  |
| Daunorubicin |  |
| Topotecan |  |
| Dabigatran |  |
| Aliskiren |  |
| Digoxin |  |

**Drugs that prolong QT interval**

The drugs listed in this section are taken from information provided by the Arizona Center for Education and Research on Therapeutics website: https://www.crediblemeds.org/. The website categorizes drugs based on the risk of inducing Torsade de Pointes (TdP).

During screening the drugs that patients are currently prescribed should be checked opposite the ArizonaCert website.

**Drugs with a known risk of Torsades de Pointes**

The following drugs prolong the QT interval and are clearly associated with a known risk of TdP, even when taken as recommended. These drugs must have been discontinued prior to the start of administration of study treatment in accordance with guidance provided in Table 3 and should not be co-administered with study treatment (osimertinib) and for a period of two weeks after discontinuing study treatment. **The list of drugs may not be exhaustive and is subject to change as new information becomes available. As such investigators are recommended to search the website to provide the most up to date information**.

| **Table 3 Drugs with a known risk of TdP** | |
| --- | --- |
| **Drug name** | **Withdrawal period prior to study treatment start** |
| Aclarubicin, Anagrelide, Ciprofloxacin, Clarithromycin, Cocaine, Droperidol, Erythromycin, Levofloxacin, Ondansetron, Papaverine hydrochloride, Procainamide, Sulpiride, Sultopride, Terfenadine Terlipressin | 2 days |
| Cilostazol, Cisapride, Disopyramide, Dofetilide, Domperidone, Flecainide, Gatifloxacin, Grepafloxacin, Ibutilide, Moxifloxacin, Oxaliplatin, Propofol, Quinidine, Roxithromycin, Sevoflurane, Sotalol, Sparfloxacin, Thioridazine | 7 days |
| Azithromycin, Bepridil, Citalopram, Chlorpromazine, Dronedarone, escitalopram, Fluconazole, Halofantrine, Haloperidol, Levomepromazine, Levosulpiride, Mesoridazine | 14 days |
| Donepezil, Terodiline | 3 weeks |
| Levomethadyl, Methadone, Pimozide | 4 weeks |
| Arsenic trioxide*, Ibogaine | 6 weeks |
| Pentamidine | 8 weeks |
| Astemizole, Probucol, Vandetanib | 4 months |
| Amiodarone, Chloroquine | 1 year |

* Estimated value as pharmacokinetics of arsenic trioxide has not been studied

**Other TdP risk Categories**

Patients receiving drugs that prolong QT interval or may increase the risk of TdP from other TdP risk categories can be enrolled, notwithstanding other exclusions and restrictions, if these drugs are considered essential for patient management and the patient has been stable on therapy. Close monitoring with ECGs and electrolytes is recommended.

Patients with congenital long QT syndrome (CLQTS) are excluded from this study.

**Guidance regardless of TdP risk category**

Following study treatment initiation if it is considered essential for patient management to give drugs known to prolong QTc interval, **regardless of TdP risk category**, close monitoring with ECGs and electrolytes is recommended.

**Appendix C Actions Required in Cases of Increases in Liver Biochemistry and Evaluation of Hy’s Law**

1 Introduction

This Appendix describes the process to be followed in order to identify and appropriately report cases of Hy’s Law. It is not intended to be a comprehensive guide to the management of elevated liver biochemistries.

During the course of the study the Investigator will remain vigilant for increases in liver biochemistry. The Investigator is responsible for determining whether a patient meets potential Hy’s Law (PHL) criteria at any point during the study.

The Investigator participates, together with Sponsor clinical project representatives, in review and assessment of cases meeting PHL criteria to agree whether Hy’s Law (HL) criteria are met. HL criteria are met if there is no alternative explanation for the elevations in liver biochemistry other than Drug Induced Liver Injury (DILI) caused by the Investigational Medicinal Product (IMP).

The Investigator is responsible for recording data pertaining to PHL/HL cases and for reporting Adverse Events (AE) and Serious Adverse Events (SAE) according to the outcome of the review and assessment in line with standard safety reporting processes.

2 Definitions

2A Potential Hy’s Law (PHL)

Aspartate Aminotransferase (AST) or Alanine Aminotransferase (ALT) ≥ 3x Upper Limit of Normal (ULN) **together with** total bilirubin (TBL) ≥ 2xULN at any point during the study following the start of study medication irrespective of an increase in Alkaline Phosphatase (ALP).

2B Hy’s Law (HL)

AST or ALT ≥ 3x ULN **together with** TBL ≥ 2xULN, where no other reason, other than the IMP, can be found to explain the combination of increases, eg, elevated ALP indicating cholestasis, viral hepatitis, another drug.

For PHL and HL the elevation in transaminases must precede or be coincident with (i.e. on the same day) the elevation in TBL, but there is no specified timeframe within which the elevations in transaminases and TBL must occur.

3 Identification of Potential Hy’s Law Cases

In order to identify cases of PHL it is important to perform a comprehensive review of laboratory data for any patient who meets any of the following identification criteria in isolation or in combination:

- ALT ≥ 3xULN
- AST ≥ 3xULN
- TBL ≥ 2xULN

The Investigator will remain vigilant for any local laboratory reports where the identification criteria are met; where this is the case the Investigator will:

- Notify the Sponsor representative
- Request a repeat of the test (new blood draw) by the local laboratory
- Complete the appropriate unscheduled laboratory CRF module(s) with the original local laboratory test result

When the identification criteria are met from local laboratory results the Investigator will without delay:

- Determine whether the patient meets PHL criteria (see 2 Definitions within this Appendix for definition) by reviewing laboratory reports from all previous visits.

The Investigator will without delay review each new laboratory report and if the identification criteria are met will:

- Notify the Sponsor representative
- Determine whether the patient meets PHL criteria (see 2 Definitions within this Appendix for definition) by reviewing laboratory reports from all previous visits
- Promptly enter the laboratory data into the laboratory CRF

4 Follow-up

4A Potential Hy’s Law Criteria not met

If the patient does not meet PHL criteria the Investigator will:

- Inform the Sponsor representative that the patient has not met PHL criteria.
- Perform follow-up on subsequent laboratory results according to the guidance provided in the Clinical Study Protocol.

4B Potential Hy’s Law Criteria met

If the patient does meet PHL criteria the Investigator will:

- Determine whether PHL criteria were met at any study visit prior to starting study treatment (See 6 Actions Required When Potential Hy’s Law Criteria are Met Before and After Starting Study Treatment)
- Notify the Sponsor/Company

The Sponsor contacts the Investigator, to provide guidance, discuss and agree on approach for the study patients’ follow-up and the continuous review of data. Subsequent to this contact the Investigator will:

- Monitor the patient until liver biochemistry parameters and appropriate clinical symptoms and signs return to normal or baseline levels, or as long as medically indicated
- Investigate the etiology of the event and perform diagnostic investigations as discussed with the Company.
- Complete the three Liver CRF Modules as information becomes available
- If at any time (in consultation with the Sponsor) the PHL case meets serious criteria, report it as an SAE using standard reporting procedures

5 Review and Assessment of Potential Hy’s Law Cases

The instructions in this Section should be followed for all cases where PHL criteria are met.

No later than 3 weeks after the biochemistry abnormality was initially detected, the Company contacts the Investigator in order to review available data and agree on whether there is an alternative explanation for meeting PHL criteria other than DILI caused by the IMP. The Sponsor Physician will also be involved in this review together with other subject matter experts as appropriate.

According to the outcome of the review and assessment, the Investigator will follow the instructions below.

If there is an agreed alternative explanation for the ALT or AST and TBL elevations, a determination of whether the alternative explanation is an AE will be made and subsequently whether the AE meets the criteria for a SAE:

- If the alternative explanation is **not** an AE, record the alternative explanation on the appropriate CRF
- If the alternative explanation is an AE/SAE, record the AE /SAE in the CRF accordingly

If it is agreed that there is **no** explanation that would explain the ALT or AST and TBL elevations other than the IMP:

- Report an SAE (report term ‘Hy’s Law’) according to standard processes.
- The ‘Medically Important’ serious criterion should be used if no other serious criteria apply
- As there is no alternative explanation for the HL case, a causality assessment of ‘related’ should be assigned.

If, there is an unavoidable delay, of over 3 weeks, in obtaining the information necessary to assess whether or not the case meets the criteria for HL, then it is assumed that there is no alternative explanation until such time as an informed decision can be made:

- Report an SAE (report term ‘Potential Hy’s Law’) applying serious criteria and causality assessment as per above
- Continue follow-up and review according to agreed plan. Once the necessary supplementary information is obtained, repeat the review and assessment to determine whether HL criteria are met. Update the SAE report according to the outcome of the review, amending the reported term if an alternative explanation for the liver biochemistry elevations is determined

6 Actions Required When Potential Hy’s Law Criteria are Met Before and After Starting Study Treatment

This section is applicable to patients << *with liver metastases*>> who meet PHL criteria on study treatment having previously met PHL criteria at a study visit prior to starting study treatment.

At the first on study treatment occurrence of PHL criteria being met the Investigator will:

- Determine if there has been a significant change in the patients’ condition^#^ compared with the last visit where PHL criteria were met^#^
- If there is no significant change no action is required
- If there is a significant change notify the Sponsor representative, who will inform the Company, then follow the subsequent process described in 4B Potential Hy’s Law Criteria met of this Appendix

^#^ A ‘significant’ change in the patient’s condition refers to a clinically relevant change in any of the individual liver biochemistry parameters (ALT, AST or total bilirubin) in isolation or in combination, or a clinically relevant change in associated symptoms. The determination of whether there has been a significant change will be at the discretion of the Investigator, this may be in consultation with the Company if there is any uncertainty.

Actions Required for Repeat Episodes of Potential Hy’s Law

This section is applicable when a patient meets PHL criteria on study treatment and has already met PHL criteria at a previous on study treatment visit.

The requirement to conduct follow-up, review and assessment of a repeat occurrence(s) of PHL is based on the nature of the alternative cause identified for the previous occurrence.

The Investigator should determine the cause for the previous occurrence of PHL criteria being met and answer the following question:

- Was the alternative cause for the previous occurrence of PHL criteria being met found to be the disease under study e.g. chronic or progressing malignant disease, severe infection or liver disease, << or did the patient meet PHL criteria prior to starting study treatment and at their first on study treatment visit as described in 6 Actions Required When Potential Hy’s Law Criteria are Met Before and After Starting Study Treatment >>?

If No: follow the process described in 4B Potential Hy’s Law Criteria met of this Appendix

If Yes:

Determine if there has been a significant change in the patient’s condition# compared with when PHL criteria were previously met

- If there is no significant change no action is required
- If there is a significant change follow the process described in Section 4.2 of this Appendix

A ‘significant’ change in the patient’s condition refers to a clinically relevant change in any of the individual liver biochemistry parameters (ALT, AST or total bilirubin) in isolation or in combination, or a clinically relevant change in associated symptoms. The determination of whether there has been a significant change will be at the discretion of the Investigator; this may be in consultation with the Company if there is any uncertainty.

References

FDA Guidance for Industry (issued July 2009) ‘Drug-induced liver injury: Premarketing clinical evaluation’: <http://www.fda.gov/downloads/Drugs/GuidanceComplianceRegulatoryInformation/Guidances/UCM174090.pdf>

Appendix D Definition of Women of Childbearing Potential and Acceptable Contraceptive Methods

1 Definition of Women of Childbearing Potential

Women of Childbearing Potential (WoCBP):

Women between menarche and menopause who have not been permanently or surgically sterilised and are capable of procreation.

Women NOT of Childbearing Potential:

Women who are permanently or surgically sterilised or postmenopausal (definitions below):

Permanent sterilisation includes hysterectomy and/or bilateral oophorectomy and/or bilateral salpingectomy but excludes bilateral tubal occlusion. Tubal occlusion is considered a highly effective method of birth control but does not absolutely exclude possibility of pregnancy. (The term occlusion refers to both occluding and ligating techniques that do not physically remove the oviducts).

- Women who have undergone tubal occlusion should be managed on trials as if they are of WoCBP (e.g. undergo pregnancy testing etc., as required by the study protocol)
- Women will be considered postmenopausal if they are amenorrhoeic for 12 months without an alternative medical cause. The following age-specific requirements apply:
- Women under 50 years old will be considered postmenopausal if they have been amenorrhoeic for 12 months or more following cessation of exogenous hormonal treatments and with luteinizing hormone and follicle-stimulating hormone levels in the postmenopausal range
- Women over 50 years of age will be considered postmenopausal if they have been amenorrhoeic for 12 months or more following cessation of all exogenous hormonal treatments

2 Acceptable Contraception Methods

Highly effective method of birth control is defined in Note 3 in International Conference on Harmonisation Guidance M3 (Nonclinical Safety Studies for the Conduct of Human Clinical Trials for Pharmaceuticals) as one that results in a low failure rate (e.g., less than 1 percent per year) when used consistently and correctly.

Note that women should have been stable on their chosen method of birth control for a minimum of 2 weeks before entering the trial. Generic names and examples of trade names are given. As trade names may vary, Investigators should check the generic name of any contraception to ensure suitability.

Acceptable contraception methods are:

- Total sexual abstinence (abstinence must be for the total duration of the trial and the follow-up period)
- Vasectomised sexual partner plus male condom (with participant assurance that partner received post-vasectomy confirmation of azoospermia)
- Tubal occlusion plus male condom
- Intra-uterine device – provided coils are copper-banded, plus male condom
- Intra-uterine system (IUS) levonorgestrel IUS (e.g., Mirena), plus male condom
- Medroxyprogesterone injections (Depo-Provera) plus male condom
- Etonogestrel implants (e.g., Implanon, Norplan) plus male condom
- Normal and low dose combined oral contraceptive pills, plus male condom
- Norelgestromin / ethinylestradiol transdermal system plus male condom
- Intravaginal device (e.g., ethinylestradiol and etonogestrel) plus male condom
- Cerazette (desogestrel) plus male condom (Cerazette is currently the only highly efficacious progesterone based pill)

3 Unacceptable Contraception Methods

The following methods are considered not to be highly effective and are therefore not acceptable contraceptive methods in AstraZeneca clinical trials:

- Triphasic combined oral contraceptives
- All progesterone only pills except, Cerazette
- All barrier methods, if intended to be used alone
- Non-copper containing intra-uterine devices
- Fertility awareness methods
- Coitus interruptus
