# Supplementary material for: Osimertinib plus consolidative radiotherapy for advanced EGFR mutant non–small cell lung cancer: a multicentre, single-arm, phase 2 trial
Source: eClinicalMedicine. 2025 Aug 26;87:103435. doi: 10.1016/j.eclinm.2025.103435 (PMC12496182; doi:10.1016/j.eclinm.2025.103435)
Supplement: SBRT + EGFR_ECM Cover Letter [file mmc3.pdf]

UTSouthwestern
Medical Center

David E. Gerber, M.D.
Professor of Internal Medicine, and Peter O'Donnell Jr. School of Public Health
David J. Bruton, Jr. Professor in Clinical Cancer Research
Harold C. Simmons Comprehensive Cancer Center

Division of Hematology and Oncology

April 23, 2025

Claudia Schafer, PhD
Editor-in-Chief, *eClinicalMedicine*

Dear Dr. Schafer:

On behalf of my co-authors, it is my pleasure to submit the manuscript **"Multi-center phase 2 trial of osimertinib plus consolidative radiotherapy for advanced *EGFR* mutant non–small cell lung cancer"** for consideration for publication as a **Research Article** in *eClinicalMedicine.*

Despite high response rates to late-generation epidermal growth factor receptor (EGFR) inhibitors, patients with advanced *EGFR* mutant non-small cell lung cancer (NSCLC) generally experience disease progression within two years. Multiple strategies to prolong the clinical benefit of EGFR inhibitors have been explored. Combination regimens including erlotinib (a first-generation EGFR inhibitor) with antiangiogenic agents, and osimertinib (a third-generation EGFR inhibitor) plus platinum-pemetrexed chemotherapy, have demonstrated improved clinical outcomes compared to single-agent EGFR inhibition. Treatment with amivantamab (an EGFR-MET bispecific antibody) and lazertinib (a third-generation EGFR inhibitor) also results in improved PFS compared to osimertinib monotherapy. Benefits of these multi-agent treatments come at a price, however, as they convey increased complexity, cost, and toxicity. Given these considerations, expert guidelines continue to recommend single-agent osimertinib as an important therapeutic option.

At the same time, local therapy such as stereotactic radiation therapy to growing lesions has emerged as an effective and widely employed approach at the time of oligoprogression for *EGFR* mutant and other NSCLC populations. Despite this growing literature, the use of stereotactic radiation or other local treatment to *prevent progression* remains understudied. The few trials investigating variable timing of local interventions in this population have employed first-generation EGFR inhibitors, a systemic therapy with markedly different efficacy and toxicity profiles than the third-generation drugs in common use for the past decade.

To address this critical question, we conducted a phase 2 trial of osimertinib plus radiation therapy at the time of expected best response. This strategy reflects (1) recognition that 8-10 weeks of systemic EGFR inhibitor results in substantial tumor shrinkage in most cases, and (2) the hypothesis that early eradication of resistant clones would synergize with ongoing systemic target inhibition.

Key study features include the following:

5323 Harry Hines Blvd., Dallas, Texas 75390-8852 Phone: 214-648-4180 Fax: 214-648-1955
david.gerber@utsouthwestern.edu utsouthwestern.edu

- Because the trial placed no restrictions on the number or location of disease sites at enrollment, about 20% of patients had 4 or more sites of metastatic disease, two-thirds had bone metastases, and half had brain metastases
- All patients received third-generation EGFR inhibitor osimertinib, with radiation therapy administered to lesions persisting after 8 weeks of osimertinib, followed by continued osimertinib until progression

Key study findings include the following:

- Among 42 enrolled patients, more than 75% received consolidative radiation therapy, primarily stereotactic radiation; primary reasons radiation was not administered were insufficient residual disease (10%) and inadequate response (5%)
- Median progression-free survival was 32 months; median overall survival was 45 months
- The toxicity profile resembled that of osimertinib monotherapy; one patient developed grade 4 pneumonitis
- Although the trial permitted both stereotactic and hypofractionated radiation therapy regimens, only about 10% of patients receiving RT received hypofractionated treatment, in all cases to the thorax

We believe that these findings will be of considerable interest to the *eClinicalMedicine* readership because of the following: (1) the unique trial design; (2) the enrollment of a broad, representative population; (3) the ease and tolerability of therapy; (4) efficacy results that compare favorably with osimertinib monotherapy and all intensified first-line regimens studied to date, including osimertinib plus chemotherapy, osimertinib plus bevacizumab, and amivantamab plus lazertinib; (5) this trial's building upon the 2024 *eClinicalMedicine* manuscript "Safety and efficacy of consolidative stereotactic radiotherapy for oligo-residual EGFR-mutant non-small cell lung cancer after first-line third-generation EGFR-tyrosine kinase inhibitors: a single-arm, phase 2 trial").

Suggested reviewers for this manuscript include the following:

Prof. Dr. Makoto Maemondo
Division of Pulmonary Medicine
Department of Internal Medicine
Iwate Medical University School of Medicine
Yahaba, JAPAN
E-mail: maemondo-ma693@aioros.ocn.ne.jp
Reason: published expertise in the treatment of advanced *EGFR* mutant non-small cell lung cancer, including intensified first-line regimens

Dr. David Planchard
Department of Medical Oncology
Institut Gustave Roussy
Paris, FRANCE
E-mail: david.planchard@gustaveroussy.fr
Reason: published expertise in the treatment of advanced *EGFR* mutant non-small cell lung cancer, including intensified first-line regimens

Ming Zeng, MD PhD
Cancer Center
Sichuan Provincial People's Hospital
University of Electronic Science and Technology of China
Sichuan, CHINA
E-mail: miller2002@yahoo.com
Reason: published expertise in the incorporation of radiation therapy in the treatment of advanced *EGFR* mutant non-small cell lung cancer

Jianjiao Ni
Department of Radiation Oncology
Fudan University Shanghai Cancer Center
Shanghai, CHINA
E-mail: fuscczzf@fudan.edu.cn
Reason: published expertise in the incorporation of radiation therapy in the treatment of oligometastatic *EGFR* mutant non-small cell lung cancer

Edward S. Kim, MD
Department of Medical Oncology & Therapeutics Research
City of Hope Cancer Center
E-mail: edwkim@coh.org
Reason: published expertise on systemic and radiation therapy for *EGFR* mutant non-small cell lung cancer

There are no excluded reviewers.

All authors had full access to the data and accept responsibility to submit for publication. The reported clinical trial conforms to CONSORT 2010 guidelines. The clinical trial has been registered in clinicaltrials.gov and was conducted in accordance with the precepts of the Helsinki Declaration. Thank you for considering our work for publication in *eClinicalMedicine*.

Respectfully,

David Gerber

David E. Gerber, M.D.

5323 Harry Hines Blvd., Dallas, Texas 75390-8852 Phone: 214-648-4180 Fax: 214-648-1955
david.gerber@utsouthwestern.edu utsouthwestern.edu
